# Cyan-Emitting Cu(I) Complexes and Their Luminescent Metallopolymers

**DOI:** 10.3390/molecules26092567

**Published:** 2021-04-28

**Authors:** Federico Ferrari, Jonas Braun, Christopher E. Anson, Bodo D. Wilts, Dafni Moatsou, Claudia Bizzarri

**Affiliations:** 1 Karlsruhe Institute of Technology, Institute of Organic Chemistry, Fritz-Haber-Weg 6, 76131 Karlsruhe, Germany; federico.ferrari@kit.edu; 2 Karlsruhe Institute of Technology, Institute of Inorganic Chemistry, Engesserstrasse 15, 76131 Karlsruhe, Germany; jonas.braun2@kit.edu (J.B.); christopher.anson@kit.edu (C.E.A.); 3 Adolphe Merkle Institute, University of Fribourg, Chemin des Verdiers 4, 1700 Fribourg, Switzerland; bodo.wilts@unifr.ch

**Keywords:** heteroleptic copper(I) complexes, functional copolymers, light-emitting metallopolymers, RuAAC

## Abstract

Copper complexes have shown great versatility and a wide application range across the natural and life sciences, with a particular promise as organic light-emitting diodes. In this work, four novel heteroleptic Cu(I) complexes were designed in order to allow their integration in advanced materials such as metallopolymers. We herein present the synthesis and the electrochemical and photophysical characterisation of these Cu(I) complexes, in combination with ab initio calculations. The complexes present a bright cyan emission (λ_em_ ~ 505 nm) in their solid state, both as powder and as blends in a polymer matrix. The successful synthesis of metallopolymers embedding two of the novel complexes is shown. These copolymers were also found to be luminescent and their photophysical properties were compared to those of their polymer blends. The chemical nature of the polymer backbone contributes significantly to the photoluminescence quantum yield, paving a route for the strategic design of novel luminescent Cu(I)-based polymeric materials.

## 1. Introduction

Luminescent transition metal complexes represent a significant class of materials that is exploited in a vast range of applications, from optoelectronic technologies, such as organic light-emitting diodes (OLEDs) [1,2], to bioimaging and sensors [3,4] or in photo(redox) catalysis [5,6,7]. Copper (I) complexes are a desirable cost-effective choice for these applications, particularly in contrast to well established light-emitting materials based on noble metals, like iridium, platinum or ruthenium [8,9]. Their high availability paired with the significantly lower price could enable the implementation of Cu(I) complexes into everyday life applications, such as electronic devices. Cu(I) complexes present tuneable emission at room temperature in solid state and solution. However, the photoluminescence quantum yield in solution often suffers from the Jahn–Teller distortion that these complexes undergo in their excited state [10,11,12]. This distortion is a consequence of the population of a metal-to-ligand charge-transfer state (MLCT), where the metal centre formally loses one electron that goes to an empty π* orbital of the coordinated ligand. Thus, the valence state of the Cu centre in its excited state can be considered +2. The structural change might activate non-radiative pathways, thereby decreasing the photoluminescence quantum yield of these complexes.

A strategy to reduce the disadvantages of the population of the MLCT, minimising the Jahn–Teller distortion, is to functionalise the coordinated ligands with bulky substituents [13,14] or increase the rigidity of ancillary ligands, such as chelating diphosphines [15,16,17]. In any case, this inconvenience is moderate in solid state. Thus, Cu(I) complexes have been indicated as promising emitters in OLED technologies [9,18,19,20].

Polymers are often used as scaffolds onto which functional moieties are immobilised to improve the stability and solubility of these functional groups [21]. The so-formed functional polymers combine the components’ properties; for example, hybrid materials that combine the properties of a coordination metal complex with those of an organic polymer have been previously reported [22]. Indeed, the covalent attachment of metal–organic complexes to polymer chains has been shown to prevent phase separation and to enhance the properties of the functional groups (e.g., in catalysis). At the same time, unique properties such as unimolecular micellization have also been reported [23,24,25]. Furthermore, such hybrids improve processability and give the metal complex increased stability [26]. Two approaches are commonly pursued: the functional group is either attached to the polymer in a post-polymerisation reaction or through direct copolymerisation, which first requires introducing a polymerisable moiety onto the functional group [21]. Both approaches have been reported for a range of polymerisation techniques. However, one commonly used approach is radical (co)polymerisation, as it is a robust reaction that is generally tolerant towards a range of functionalities. Despite the increasing number of highly emissive Cu(I) complexes in the scientific literature, very few works report luminescent metallopolymers based on copper [27,28,29,30,31].

**Figure 1:**
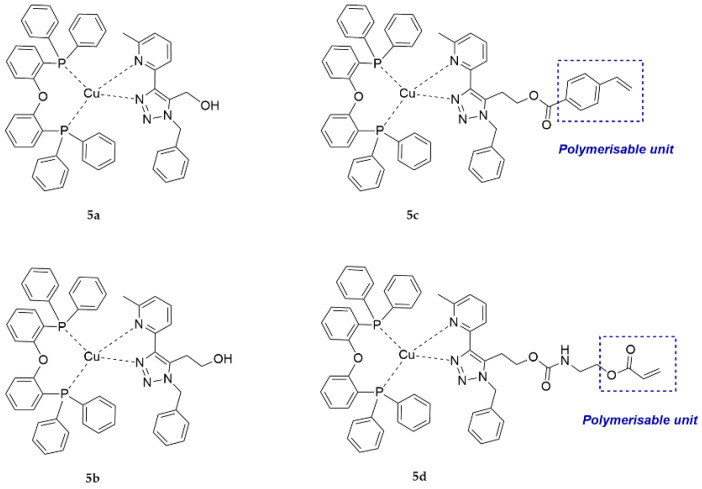
Structures of the copper(I) complexes that were investigated in this work. The structures can be classified as those bearing a hydroxyl group (**5a**, **5b**) or a polymerisable unit (**5c**, **5d**).

In this work, four novel heteroleptic copper (I) complexes giving a bright cyan emission in their solid state were synthesised (Figure 1). The coordination of Cu(I) to the diphosphine bis[(2-diphenylphosphino)phenyl]ether (DPEPhos) gave more stability to the final complex since the steric hindrance of this chelating diphosphine is elevated. The chelating diimines, whose electronic properties determine the emission of the complex, are based on pyridinyl-1,2,3-triazole. Two of those diimines carry a hydroxyl group (**5a** and **5b**), which can be used in carbamate or esterification reactions. Further functionalisation of the diimine ligand with vinyl derivatives gave access to polymerisable complexes (**5c** and **5d**). Herein, we present the electrochemical and the photophysical characterisation together with computational studies of the new Cu(I) complexes. Radical copolymerisation of the polymerisable ligands with suitable comonomers yielded functional polymers with controlled loadings. The properties of the copper complexes in bulk were directly compared to those of their respective copolymers. Moreover, polymer blends of the complexes **5c** and **5d** were also investigated to compare the metal complex-functional repeat unit of the corresponding copolymer.

## 2. Results and Discussion

### 2.1. Synthesis

#### 2.1.1. Ligand Synthesis

**Scheme 1:**
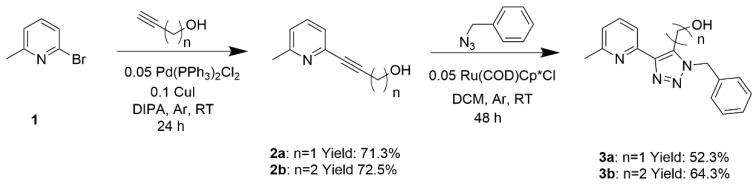
Synthetic procedure for the preparation of the ligands **3a** and **3b**.

The chelating diimine ligands were synthesised in two steps, as shown in Scheme 1. First, a Sonogashira cross-coupling reaction [32,33] was carried out on the substrate 2-bromo-6-methylpyridine, **1**, with one equivalent of alkynyl alcohols, propargyl alcohol or 3-butynol, to give the products **2a** and **2b**, respectively. The following step was forming the 1,2,3-triazoles **3a** and **3b**, obtained from a cyclisation reaction between **2a** or **2b** and benzyl azide via a ruthenium–alkyne–azide-cycloaddition (RuAAC) [34]. A Cu-catalysed cycloaddition [35] was not possible because the triple bonds of **2a** and **2b** are not terminal. As all organic azides, benzyl azide is a hazardous and potentially explosive material. Therefore, particular care is needed [36]. These ligands differ in the number of methylene units that separate the triazole ring from the hydroxyl group, which was subsequently found to drastically affect the reactivity of the latter, e.g., in esterification reactions. 

**Figure 2:**
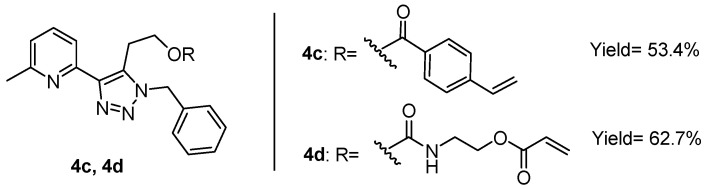
Structure of the ligands bearing a polymerisable unit. Ligand **4c** has a styrenic functionality, while **4d** has an acrylate unit.

The alcohol function of **3a** and **3b** was used to introduce a polymerisable unit, an acrylate or styrene, via carbamate formation or a Steglich esterification [37]. These two functionalities were chosen as they can readily undergo radical polymerisation [38]. The acrylate was particularly interesting as its corresponding polymer does not absorb in the near UV. This UV transparency was anticipated to prevent the undesirable quenching of the complex fluorescence. In contrast, styrene and its polymers absorb at 295 nm, which was envisioned to interfere with the photophysical properties of the metal complex. The modification of **3a** was found to suffer from poor yields and the subsequent polymerisation of the product was unsuccessful. For the styrenic monomer, vinyl benzoic acid was used to perform a Steglich esterification with triazole **3b** to yield monomer **4c** (Figure 2). To obtain the acrylate monomer (**4d**), triazole **3b** was reacted with 2-isocyanatoethyl acrylate yielding the corresponding urethane in reasonable yield and purity (see Figure 2).

#### 2.1.2. Copper(I) Complexes Synthesis

**Scheme 2:**
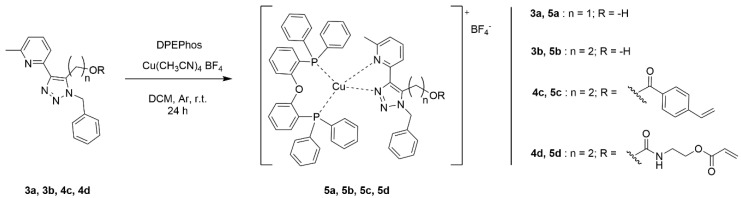
Synthetic procedure for the synthesis of the Cu(I) complexes.

Ligands **3a** and **3b**, as well as monomer-ligands **4c** and **4d**, were used to obtain the respective heteroleptic copper complexes. The synthesis of all the complexes started from the formation of the diphosphine complex by mixing bis[(2-diphenylphosphino)phenyl]ether (DPEPhos) and a copper precursor tetrakis(acetonitrile) copper(I) tetrafluoroborate [39]. Subsequently, the chelating ligands (**3a**, **3b**, **4c**, and **4d**) were added to the stirring mixture, thus allowing the ligand exchange with one of the DPEPhos and resulting in the formation of the respective heteroleptic complexes (**5a**, **5b**, **5c** and **5d**) that are shown in Scheme 2. The evaporation of the solvent gave pale yellow powders, which were found to exhibit luminescence when placed under UV light (350 nm). Purification was done by recrystallisation.

**Figure 3:**
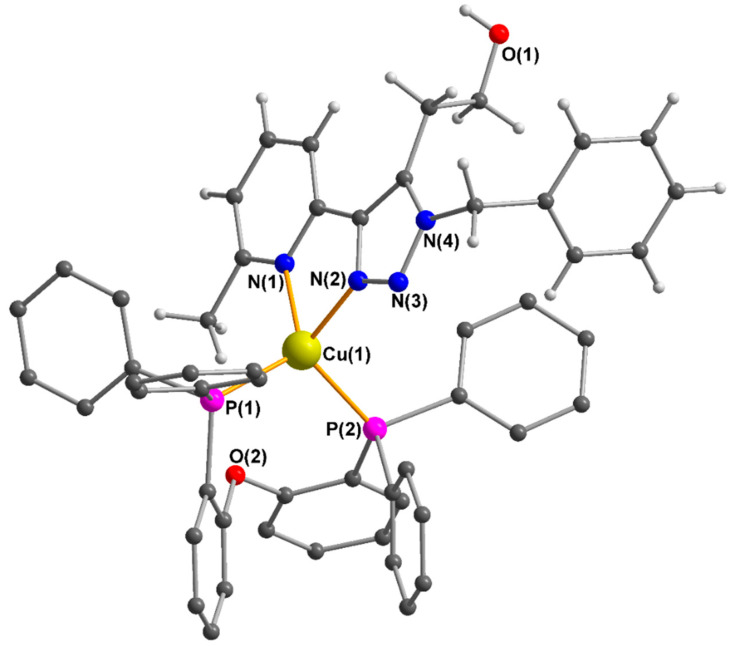
Molecular structure of the Cu(I) complex in **5b** (counteranion, lattice solvent and hydrogen atoms of the DPEPhos ligand omitted for clarity). Selected bond lengths (Å) and angle (°): Cu(1)-N(1) 2.112(3), Cu(1)-N(2) 2.048(4), Cu(1)-P(1) 2.2371(12), Cu(1)-P(2) 2.2459(12), N(1)-Cu(1)-N(2) 79.56(14).

Needle-shaped crystals of **5b** were obtained by the slow diffusion of cyclohexane into a concentrated solution in dichloromethane. The molecular structure of complex **5b** was determined by X-ray diffraction, shown in Figure 3, and agrees with heteroleptic Cu(I) complexes with similar ligands [40,41]. Bond valence sum analysis for Cu(1) in **5b** gave a calculated oxidation state of 1.007 [42]. Further parameters are described in the experimental section.

### 2.2. Synthesis and Structural Characterisation of Metallopolymers with Cu(I) Complexes

In order to access functional polymeric materials with different physical properties, but also to take advantage of the different photophysical properties of the complexes (*vide infra*), styrene (**St**) and methyl acrylate (**MA**) were copolymerised with the corresponding styrenic (**4c**) and acrylate-functional (**4d**) ligands, respectively, to yield two copolymers: poly(**St**-*co*-**4c**) (**6c**) and poly(**MA**-*co*-**4d**) (**6d**) (molecular weights are reported in Table 1). These were subsequently used to form the heteroleptic Cu(I) complexes, similarly to **5c** and **5d**, by the addition of DPEPhos and Cu(CH_3_CN)_4_BF_4_. Consequently, the polymer-supported complexes were obtained, namely **7c** and **7d**, with a targeted complex loading of 10 mol%. It is noted that the direct copolymerisation of the corresponding complexes (**5c** and **5d**) was unsuccessful, presumably due to the copper interfering with the radical process [43].

**Figure 4:**
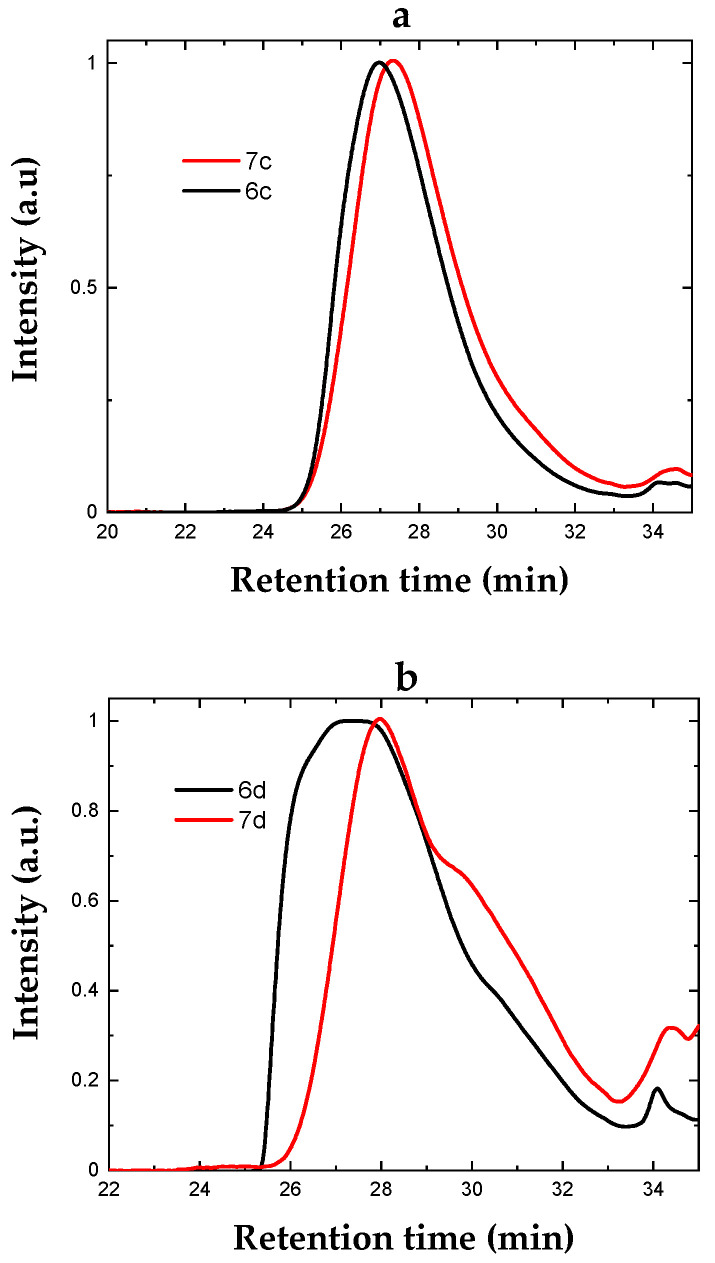
(**a**,**b**) SEC of the styrenic and acrylate copolymers before (**6c**, **6d**) and after complexation (**7c**, **7d**), respectively.

The copolymerisation of the styrenic ligand **5c** with styrene yielded a copolymer with a single molecular weight distribution in the size exclusion chromatogram (SEC, Figure 4), indicating the reasonable control of the reaction, in contrast to the copolymerisation of the acrylate ligand **5d** with methyl acrylate that resulted in a broad and multimodal distribution. It is noted that higher molecular weights are attainable by reducing the ratio of the initiator (AIBN) equivalents to the monomers. Nonetheless, the NMR analysis of both copolymers indicated ligand loading in reasonable agreement with the targeted value, namely 8 mol% for **6c** and 11 mol% for **6d** (Supporting Information, Figures S14 and S15). Subsequent complexation, however, was found highly efficient for the styrenic copolymer with an overall complex loading of 7 mol% for **7c,** whereas, for the acrylate copolymer **7d**, it was 5 mol% (Supporting Information, Figures S16 and S17). This was attributed to the dispersity of copolymer **6d** as we hypothesised that the uncontrolled polymerisation reaction might have resulted in the poor distribution of the ligand along the polymer chain. The SEC data of both complexed copolymers indicated a decrease in the apparent molecular weight, attributed to the interaction of the newly introduced species with the column material. 

In order to assess the effect of the covalent immobilisation of the metal complexes onto the polymers, the respective blends were also prepared by simply mixing in solution and subsequently allowing the drying of poly(styrene) (PS) (*M*_n_ = 12.9 kg/mol, *Ð* = 1.50) with **5c**, yielding PS/**5c**, and poly(methyl acrylate) (PMA) (*M*_n_ = 17.0 kg/mol, *Ð* = 1.68) with **5d**, yielding PMA/**5d**, targeting in both cases an average loading comparable to that of the ligand-copolymers (10 mol%).

### 2.3. Photophysical Characterisation of the Cu(I) Complexes

#### 2.3.1. In Solution

**Figure 5:**
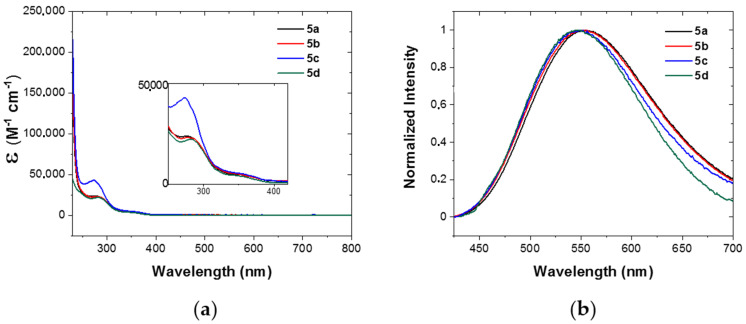
UV–vis spectroscopy of complexes **5a** (black curves); **5b** (red curves); **5c** (blue curves); and **5d** (green curves) in DCM solutions at r.t. (**a**) Molar extinction coefficients (ε) in the UV–visible range of the electromagnetic spectrum—a zoom-in is shown in the inset; (**b**) emission of Ar-saturated solutions (concentration ~μM; λ_exc_ = 370 nm).

The electronic absorption spectroscopy of the four new Cu(I) complexes was investigated in dichloromethane (DCM) solutions at room temperature. The molar extinction coefficients are reported in Table 2 and shown in Figure 5a. As expected from the strong similarity of their chemical structures, we can identify many correspondences in their UV–vis absorption spectra. All the complexes present very intense extinction coefficients below 300 nm. These are attributed to spin-allowed π–π* transitions, i.e., ligand-centred transitions (^1^LC), mainly located on the chelating phosphine DPEPhos and the diimine ligand [15]. The additional contribution of the vinyl-phenyl moiety on the ligand of complex **5c** is highlighted by the absorption band with a maximum at 273 nm. A low-intensity shoulder is present in all the complexes, ranging from 320 nm to 400 nm. This shoulder was assigned to the electronic transitions from the copper centre to the π* orbital of the diimine ligand, populating a singlet metal-to-ligand charge-transfer state (^1^MLCT), typical for Cu(I) complexes [12,14].

All the new complexes are luminescent at room temperature in Ar-saturated solutions (Figure 5b). In particular, when excited at their MLCT band, they show an approximately 180 nm-broad structureless emission band centred at about 550 nm, with only slight differences among the complexes (Table 2). This emission profile is typical for Cu(I) complexes of type Cu(PP)(NN), and it is assigned to the excited MLCT state. The Stokes shifts are substantial (1.33 eV for **5a** and **5b**, 1.24 eV for **5c** and 1.30 eV for **5d**), which, together with the observation that the luminescence is quenched in air-equilibrated solutions, are firm evidence of a triplet nature of the excited state. However, as several Cu(I) complexes have been demonstrated to show thermally activated fluorescence (TADF), [19,44,45,46,47,48,49,50,51,52,53,54,55,56,57,58] this cannot be excluded at this stage. The excited state lifetimes in solution are in the order of one microsecond, in addition to complex **5c** which shows a lifetime of 83 ns. Further analyses are ongoing to investigate the processes involved in the excited state in more detail and whether these complexes show TADF.

The photoluminescence quantum yields (Φ) of compound **5b** and its acrylate derivative **5d** are very similar (3.7% and 3.5% for **5b** and **5d**, respectively). The low emission of Cu(I) complexes even in non-coordinating solvents, such as DCM, is attributed to the Jahn–Teller distortion that the complex undergoes upon excitation. The presence of the methyl substituent α to the pyridine might have impeded the distortion to some extent, as this was also demonstrated in other studies [40,59,60,61]. Complex **5c** presents a Φ of only 0.9%. The presence of the styrene moiety on the diimine is likely responsible for the lower quantum yield with respect to the one of the precursor complex **5b**. This effect was also observed with other Cu(I) complexes containing a styrene moiety [27]. Moreover, ab initio computational studies confirm that styrene is involved in the lowest unoccupied molecular orbital (LUMO) of complex **5c** (vide infra; see Section 2.4 and the Supporting Information, Figures S44–S46). 

**Table 2:** Photophysical properties of Cu(I) complexes **5a**, **5b**, **5c** and **5d** in solution at room temperature.

Sample	λ_abs_ (nm)	ε ×10^−3^ (M^−1^ cm^−1^)	λ_em_^a^ (nm)	Φ ^b^	τ (ns) ^c^
**5a**	347 276 250	4.3 23.5 27.3	552	n.a.	850
**5b**	347 276 250	4.4 22.7 28.5	552	0.037	1050
**5c**	354 273 250	4.6 43.0 38.6	548	0.009	83
**5d**	347 281 240	3.6 22.0 30.7	545	0.035	1500

^a^ in Ar-saturated DCM; ^b^ photoluminescence quantum yields were measured with the relative method using Ru(bpy)_3_Cl_2_ in aerated water solution as standard (Φ = 0.040) [62]; ^c^ lifetimes were measured with TCSPC and NanoLED excitation at 366 nm.

#### 2.3.2. In Solid State

We carried out the photoluminescence quantum yield measurements from the samples in bulk in order to assess the photophysical properties of the complexes and their metallopolymers in the solid state (Table 3). For the sake of accuracy, the homopolymers of PMA and PS were also measured and exhibited no luminescence, as expected. Initially, the photoluminescence quantum yields of the crystalline powders of the four Cu(I) complexes were estimated. The highest quantum yield of 17% was obtained for the complex bearing the acrylate functionality (**5d**) (Figures S52–S55). Overall, the trend of data was in agreement with the measurements carried out in solution.

Furthermore, drop-casted films of the metallopolymers **7c** and **7d** were evaluated and compared with those films of polymeric blends with complexes **5c** and **5d** (a photograph of drop-casted PMA/**5d** is shown in Figure S75). The emission colour of these films is cyano-green, as the chromaticity diagram CIE 1931 reports (Figure S66). The polymeric blends were obtained dispersing **5c** or **5d** in both homopolymers, PS and PMA, at comparable loading (10%). Neither drop-casted film of blends PS/**5c** nor PMA/**5c** exhibited luminescence when excited at 350 nm, while the metallopolymer **7c** (loading 8%) presents an emission with Φ of 10% (Figures S56–S61). This points to the significant effect of the conjugation in the styrene moiety of the free **5c**, which appears to be responsible for the low or absent photoluminescence. This hypothesis was validated by computational analysis (vide infra). On the contrary, the emission of the metallopolymer **7c** is encouraging and further supports the development of metallopolymers as materials with advanced properties.

In contrast, the double bond of the acrylate unit of complex **5d** does not have an unfavourable effect on its emission. In fact, both the PS/**5d** and the PMA/**5d** blends were emissive. In particular, PMA/**5d** exhibited a Φ of 21%, which was almost twice the Φ of PS/**5d** (Φ = 13%). This difference was attributed to the different chemical environment provided by the respective polymers. Thus, PS induces nonradiative pathways of **5d** and this quenching may be due to π-stacking between the aryl groups of the complex interacting with the pendant phenyl rings of the PS backbone. Alike the metallopolymer **7c**, the film of **7d** was luminescent and presented a Φ of 10%. It must be remarked that the complex loading of **7d** was only 5%, due to the poor yield of the DPEPhosCu^+^ coordination onto the copolymer **6d** (see Section 2.2). Thus, the metallopolymer **7d** also shows promising emissive properties in line with the respective blend in PMA. 

### 2.4. Computational Studies of Cu(I) Complexes

To gain further insight into the photophysical and electronic properties of the new copper complexes, theoretical ab initio quantum chemical calculations were performed. We omitted the investigation of **5a** since this complex was not further functionalised with a monomeric unit (e.g., an acrylate or a styrene moiety) and therefore was not part of the final metallopolymers studied in this work. Moreover, **5a** differs from complex **5b** only by a -CH_2_- unit, having in position 5 of the triazole ring a hydroxymethyl substituent, while **5b** has a hydroxyethyl at the same position. Therefore, we expect **5a** to have comparable properties to **5b**. The geometry of complexes **5b**, **5c** and **5d** was then optimised at the density functional theory (DFT) level (PBE0/6-31G**/LANL2DZ, in vacuum) using the QChem package [64].

**Figure 6:**
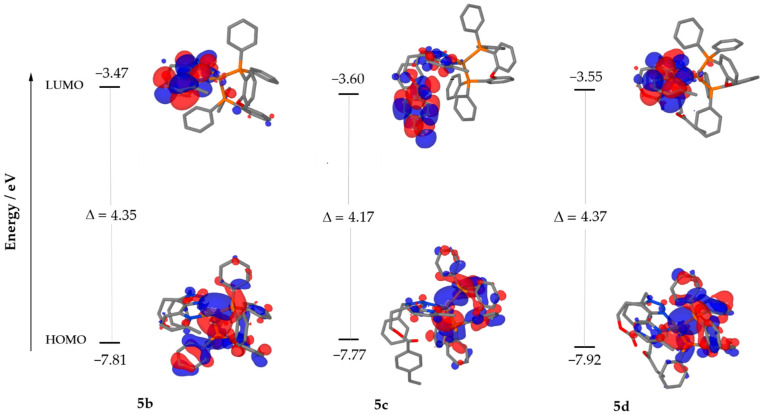
Isovalue contours calculated for the HOMO and LUMO of **5b** (**left**), **5c** (**centre**) and **5d** (**right**). Hydrogen atoms are omitted for clarity. Red indicates the negative and blue the positive sign of the wave-function.

The optimised geometries were used to predict absorption spectra via time-dependent DFT (TDDFT), from where the energies and molecular orbital compositions of triplet and singlet states were obtained. The calculations indicate that the transition to the first excited singlet is described by the electronic transition from the highest occupied molecular orbital (HOMO) to the LUMO. As shown in Figure 6, the HOMO of the three complexes is mainly located on the Cu centre with some contribution from the chelating phosphine, whilst in contrast, the LUMO is completely delocalised over the diimine ligand. Thus, upon HOMO → LUMO excitation, the charge is transferred from Cu(PP) to the pyridinyl-triazole (NN) ligand, indicating an MLCT character of the excited state. The convoluted data, obtained from the predicted vertical transitions, are in accordance with the experimental electronic absorption spectra. This agreement is presented in Figures S44–S46 for **5b**, **5c** and **5d**.

Interestingly, in complex **5c**, the LUMO is strongly localised on the styrene unit appended to the diimine ligand through the ester functionality, showing less contribution of the pyridinyl-triazole moiety directly coordinating the metal centre. This result justifies the experimental observation of the lower luminescence of **5c** when compared to **5b** and **5d**. In other words, the styrene unit is responsible for a significant electronic involvement with the consequent deactivation of the excited state. Furthermore, the acrylate unit of **5d** does not participate in the electronic transitions, explaining why the photoluminescence quantum yield of **5d** is virtually identical to **5b**. The HOMO and LUMO values are reported in Table 4. Furthermore, the trend of electronic band gaps obtained from theoretical calculations is in agreement with the electrochemical data presented subsequently.

### 2.5. Electrochemical Properties of Cu(I) Complexes

**Figure 7:**
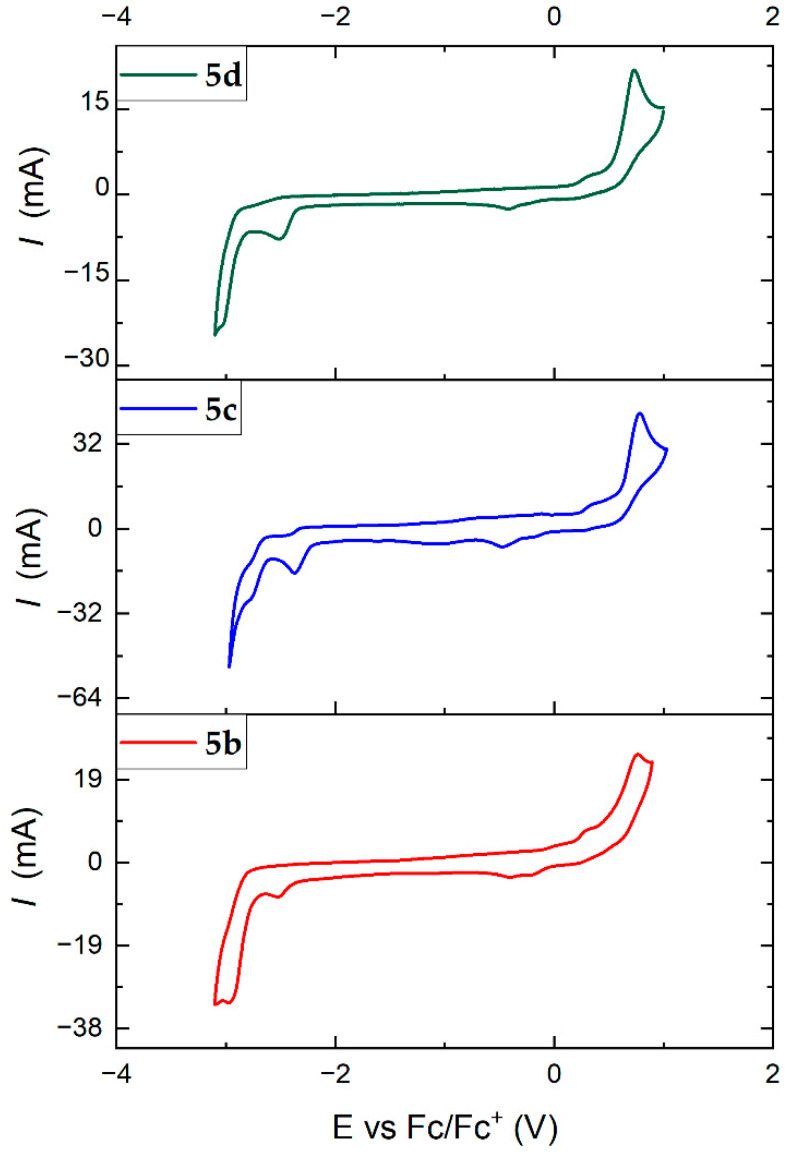
Cyclic voltammetry of Cu(I) complexes **5b** (**bottom**), **5c** (**centre**), **5d** (**top**) in *N*,*N*-dimethylformamide (0.1 M TBAPF_6_) at a scan rate of 100 mV/s.

The electrochemical behaviour of the new Cu(I) complexes was investigated by cyclic voltammetry (CV), which was performed in DCM (Figure S47) and in *N*,*N*-dimethylformamide (DMF) solutions with 0.1 M tetrabutylammonium hexafluorophosphate (TBAPF_6_) as the supporting electrolyte. The three-electrode cell was equipped with glassy-carbon as the working electrode, and a Pt wire was used as the auxiliary electrode. Since Ag wire was used as a quasi-reference electrode, we reported all the values versus the ferrocene/ferricinium (Fc/Fc^+^) redox couple used as the internal standard. The obtained results are displayed in Figure 7, and the derived values are reported in Table 4. We observe an irreversible oxidation process around 0.6 V for the three investigated complexes, attributed to the oxidation of Cu(I) to Cu(II). Note that this oxidation process becomes reversible when the measurement is performed in dichloromethane. Therefore, although the electrochemical window of DCM is too small in reduction, a more detailed study of the oxidation process was carried out in this solvent for all the complexes. As is displayed in the Supporting Information (Figures S47–S51), we performed the electrochemical measurement at different scan rates. The peak intensity of the anodic process was plotted versus the square root of the scan-rate. An excellent linear fit confirms that the oxidation processes of all four complexes are diffusion-controlled (Figures S48–S51), as the anodic peak currents of their oxidation process follow the Randles–Sevcik equation [65]. In the reduction, two irreversible processes are observed for the complexes **5b**, **5c** and **5d**, which are attributed to the reduction in the respective diimine chelating ligand. In particular, the first reduction can be associated with the LUMO of the complex. Thus, the electrochemical band gaps were estimated to be 3.15 V, 3.01 V and 3.12 V for complexes **5b**, **5c** and **5d**, respectively. Although these band gaps deviate considerably from those calculated from the computational studies, there is a correlation, revealing that the electrochemical and theoretical HOMO–LUMO gaps are consistent.

## 3. Materials and Methods

### 3.1. Materials

Solvents of p.a. quality (per analysis) were commercially acquired from Sigma Aldrich, Carl Roth, or Acros Fisher Scientific and unless otherwise stated, used without further purification. Anhydrous solvents were purchased from Carl Roth, Acros, or Sigma Aldrich (less than 50 ppm of H_2_O, kept over molecular sieves). 2-Bromo-6-methylpyridine (98%) and benzyl azide (94%) were bought from Alfa Aesar. Propargyl alcohol (98%), butynyl alcohol (97%), bis(triphenylphosphine)palladium(II) dichloride (98%), and chloro­(pentamethylcyclopentadienyl)(cyclooctadiene)ruthenium(II) (99.5%) were purchased from Sigma Aldrich.

Reaction mixtures were purified by flash chromatography. For the stationary phase of the column, silica gel, produced by Merck (silica gel 60, 0.040 × 0.063 mm, 260–400 mesh ASTM) and sea sand by Riedel de Haën (baked out and washed with hydrochloric acid) were used. 

Air- and moisture-sensitive reactions were carried out under argon atmosphere in previously baked out apparatuses with standard Schlenk techniques. Liquid reagents and solvents were injected with syringes and stainless-steel cannulas of different sizes. 

### 3.2. Synthetic Procedures

#### 3.2.1. 2-(2-Propyn-1-ol)-6-methylpyridine (**2a**) and 2-(3-Butynyl-1-ol)-6-methylpyridine (**2b**)

In a typical procedure, into a flame-dried 50 mL two-neck round bottom flask, 25 mL of dry diisopropylamine (DIPA) was added under argon, followed by the addition of 1-bromo-6-methylpyridine **1** (1.99 mL, 17.44 mmol, 3.00 g, 1 equiv.), the alcohol (propargyl alcohol **8a** or 3-butynol **8b**) (17.44 mmol, 1 equiv.), copper(I) iodide (1.74 mmol, 0.33 g, 0.1 equiv.) and bis(triphenylphosphine)palladium(II) dichloride (0.872 mmol, 0.61 g, 0.05 equiv.). The reaction mixture was allowed to stir at room temperature for 24 h. After the removal of the solvent under reduced pressure, the residue was dissolved in 5 mL of DCM and cyclohexane was added dropwise. The biphasic solution obtained was then left in the fridge for 48 h until a precipitated was formed. The pure product was isolated as a brown solid. Yields are shown in Table 5.

**2a:**

**^1^H-NMR** (CDCl_3_, 400 MHz) *δ* (ppm): 7.50 (1H, t, ^3^*J* = 7.7 Hz, -N=C-CH=C**H**-), 7.22 (1H, d, ^3^*J* = 7.7 Hz, CH_3_-C=C**H**-), 7.07 (1H, d, ^3^*J* = 7.7 Hz, -N=C-C**H**=CH-*),* 4.53 (2H, s, CH_2_), 2.51 (3H, s, CH_3_). 

**^13^C-NMR** (CDCl_3_, 101 MHz) *δ* (ppm): 158.78, 142.04, 136.78, 124.39, 88.58, 84.25, 50.99, 24.24.

**ESI-MS** *m*/*z* [M+H]^+^ calculated for C_9_H_9_NO: 148.08, found: 148.08.

**IR** *ν* (cm^−1^) I3186.1 (m), 3056.6 (w), 2955.8 (w), 2916.7 (w), 2852.9 (w), 1590.0 (m), 1569.4 (m), 1460.4 (s), 1439.8 (s), 1376.1 (m), 1355.5 (s), 1289.7 (w), 1236.2 (m), 1168.3 (s), 1094.3 (w), 1042.9 (s), 1018.2 (s), 997.6 (vs), 962.6 (m), 892.7 (w), 851.6 (w), 796.0 (vs), 746.7 (w), 695.2 (vs), 602.7 (w), 575.9 (vs), 541.0 (m), 532.7 (m), 440.2 (w).

^1^H-NMR, ^13^C-NMR, IR spectra are shown in the Supporting Information: Figures S2, S18, S28, respectively. 

**2b:**

**^1^H-NMR** (CDCl_3_, 400 MHz) *δ* (ppm): 7.50 (1H, t, ^3^*J* = 7.7 Hz,-N=C-CH=C**H**-), 7.20 (1H, d, *^3^J* = 7.7 Hz, CH_3_-C=C**H**-), 7.06 (1H, d, ^3^*J* = 7.7 Hz, -N-C-C**H**=CH-), 3.86 (2H, t, ^3^*J* = 6.6 Hz, -CH_2_-C**H**_2_-OH), 3.41 (1H, br, -OH), 2.71 (2H, t, ^3^*J* = 6.6 Hz, -C**H**_2_-CH_2_-OH), 2.52 (3H, s, CH_3_).

**^13^C-NMR** (CDCl_3_, 101 MHz) *δ* (ppm): 158.74, 142.70, 136.74, 122.57, 88.58, 84.25, 50.99, 24.24.

**ESI-MS** *m*/*z* [M+H]^+^ calculated for C_10_H_11_NO: 162.08, found: 162.08.

**IR** *ν* (cm^−1^) 3186.1 (w), 3182.0 (w), 3073.0 (w), 3058.6 (w), 2935.2 (w), 2910.5 (w), 2867.3 (w), 2840.6 (w), 2231.7 (w), 1631.1 (vw), 1585.9 (s), 1571.5 (s), 1503.6 (w), 1456.3 (vs), 1427.5 (s), 1376.1 (m), 1300.0 (w), 1289.7 (w), 1256.8 (w), 1236.2 (m), 1180.7 (w), 1166.3 (m), 1119.0 (w), 1100.4 (w), 1059.3 (vs), 1044.9 (vs), 1012.0 (s), 999.7 (s), 915.3 (w), 847.4 (w), 796.0 (vs), 775.5 (m), 752.8 (m), 719.9 (s), 695.2 (vs), 600.6 (m), 573.9 (w), 553.3 (m), 541.0 (s), 487.5 (w), 471.0 (w), 442.2 (s), 419.6 (s).

^1^H-NMR, ^13^C-NMR, IR spectra are shown in the Supporting Information: Figures S3, S19 and S29, respectively. 

#### 3.2.2. (1-Benzyl-4-(6-methylpyridin-2-yl)-1H-1,2,3-triazol-5yl)methanol (**3a**) and 2-(1-Benzyl-5-(6-methylpyridin-2-yl)-1H-1,2,3-triazol-4-yl)ethan-1-ol (**3b**)

In a typical procedure, in a flame-dried 50 mL two-neck round bottom flask, 25 mL of dichloromethane (DCM) was added under argon, followed by the addition of the corresponding alkyne (**2a** or **2b**) (0.67 mmol, 1 equiv.), benzyl azide (**9**) (0.30 mL, 1.34 mmol, 0.31 g, 2.0 equiv.), and chloro(pentamethylcyclopentadienyl)(cyclooctadiene)ruthenium(II) (Ru(COD)Cp*Cl) (0.03 mmol, 0.013 g, 0.05 equiv.). The reaction mixture was allowed to stir at room temperature for 48 h. After the removal of the solvent under reduced pressure, the residue was purified by column chromatography on silica gel using a mixture of dichloromethane and methanol and the desired isomer was isolated (*R*_f,**3a**_ = 0.20, *R*_f,**3b**_ = 0.22, *R*_f,**3e**_ = 0.46, *R*_f, **3f**_ = 0.33 in 5% v/v of methanol), yields are shown in Table 6. 

**3a:**

**^1^H-NMR** (CDCl_3_, 400 MHz) *δ* (ppm): 7.70 (1H, t, ^3^*J* = 7.8 Hz, *para*-pyridine), 7.47 (1H, d, ^3^*J* = 7.8 Hz, *ortho*-pyridine), 7.30–7.10 (6H, m, Ar), 5.91 (2H, s, -N-C**H**_2_-), 5.01 (1H, br, -OH), 4.81 (2H, app s, -C**H**_2_-OH), 2.63 (3H, s, CH_3_).

**^13^C-NMR** (CDCl_3_, 101 MHz) *δ* 158.83, 146.54, 145.80, 137.64, 135.59, 134.59, 128.71, 128.01, 127.29, 123.32, 121.33, 56.02, 52.95, 24.37.

**ESI-MS** *m*/*z* [M+H]^+^ calculated for C_16_H_16_N_4_O: 281.14, found: 281.14.

**IR** *ν* (cm^−1^) 2844.7 (w), 1604.4 (m), 1577.6 (m), 1550.9 (w), 1495.4 (w), 1474.8 (m), 1454.2 (s), 1446.0 (s), 1376.1 (w), 1339.0 (w), 1316.4 (w), 1295.9 (w), 1281.5 (w), 1252.7 (m), 1242.4 (m), 1203.3 (w), 1158.0 (m), 1088.1 (w), 1071.6 (w), 1055.2 (m), 1038.7 (vs), 1009.9 (m), 985.3 (w), 970.9 (w), 935.9 (w), 909.2 (w), 855.7 (w), 808.4 (vs), 756.9 (s), 740.5 (s), 715.8 (vs), 707.6 (vs), 695.2 (s), 680.8 (m), 647.9 (m), 617.1 (w), 588.3 (w), 578.0 (m), 559.5 (w), 538.9 (vw), 512.2 (w), 469.0 (w).

^1^H-NMR, ^13^C-NMR, IR spectra are shown in the Supporting Information: Figures S4, S20 and S30, respectively. 

**3b:**

**^1^H-NMR** (CDCl_3_, 400 MHz) *δ* (ppm): 7.99 (1H, d, ^3^*J* = 7.8 Hz, *ortho*-pyridine), 7.71 (1H, t, ^3^*J* = 7.8 Hz, *para*-pyridine) 7.39–7.29 (m, 3H, *m*- and *p*-benzyl), 7.22–7.16 (m, 2H, *o*-benzyl), 7.11 (1H, d, ^3^*J* = 7.8 Hz, *ortho*-pyridine), 6.68 (1H, br, -CH_2_-O**H**), 5.59 (2H, s, -CH_2_), 3.68 (2H, t, ^3^*J* = 8.8 Hz, -C**H**_2_-OH), 3.16 (2H, t, ^3^*J* = 8.8 Hz, -C**H**_2_-CH_2_-OH), 2.54 (3H, s, -CH_3_).

**^13^C-NMR** (CDCl_3_, 101 MHz) δ 157.25, 149.89, 145.48, 138.01, 135.01, 133.78, 129.20, 128.58, 127.17, 122.57, 119.13, 61.33, 52.15, 26.51, 23.70.

**ESI-MS** [M+H]^+^ *m*/*z* calculated for C_17_H_18_N_4_O: 295.15, found: 295.15.

**IR** *ν* (cm^−1^) 3200.5 (w), 3089.5 (w), 3058.6 (w), 3033.9 (w), 3005.1 (w), 2953.7 (w), 2924.9 (w), 2855.0 (w), 1602.3 (m), 1575.6 (s), 1497.4 (w), 1478.9 (m), 1450.1 (vs), 1376.1 (m), 1355.5 (m), 1334.9 (w), 1289.7 (w), 1238.3 (m), 1205.3 (w), 1182.7 (w), 1158.0 (m), 1108.7 (w), 1092.2 (w), 1059.3 (vs), 1047.0 (vs), 1016.1 (s), 997.6 (s), 962.6 (w), 905.0 (w), 892.7 (w), 866.0 (m), 851.6 (w), 796.0 (vs), 748.7 (s), 724.0 (vs), 695.2 (vs), 619.1 (m), 602.7 (m), 575.9 (s), 541.0 (m), 530.7 (m), 491.6 (m), 458.7 (m), 440.2 (m), 417.6 (w), 407.3 (w).

^1^H-NMR, ^13^C-NMR, IR spectra are shown in the Supporting Information: Figures S5, S21 and S31, respectively. 

#### 3.2.3. 2-(1-Benzyl-4-(6-methylpyridin-2-yl)-1H-1,2,3-triazol-5-yl)ethyl 4-vinylbenzoate (**4c**)

In a flame-dried 50 mL two-neck round bottom flask, 25 mL of DCM was added under argon, followed by the addition of **3b** (0.67 mmol, 0.2 g, 1 equiv.), 4-vinylbenzoic acid (1.02 mmol, 151.0 mg, 1.5 equiv.), *N*-(3-di­methyl­amino­propyl)-*N*′-ethyl carbodiimide (EDCI, 0.240 mL, 1.36 mmol, 210.96 mg, 2 equiv.) and 4-(dimethylamino)pyridine (DMAP, 0.136 mmol, 16.6 mg, 0.2 equiv.). The suspension was allowed to stir at room temperature for 48 h. After the removal of the solvent under reduced pressure, the residue was dissolved in 20 mL of DCM and washed twice with 20 mL of NaOH solution (1.0 M) and twice with brine. The solution was dried over anhydrous magnesium sulphate and after filtration, the solvent was removed under reduced pressure. The product was isolated as a yellow viscous liquid with a yield of 53.4%

**^1^H-NMR** (CDCl_3_, 400 MHz) *δ* (ppm): 7.99 (1H, d, ^3^*J* = 7.8 Hz, *ortho*-pyridine ), 7.81 (2H, d, ^3^*J* = 8.2 Hz , *meta*-styrene), 7.57 (1H, t, ^3^*J* = 7.8 Hz, *para*-pyridine ), 7.38 (2H, d, ^3^*J* = 8.2 Hz, *ortho*-styrene ), 7.31-7.16 (m, 5H, benzyl), 6.96 (1H, d, ^3^*J* = 7.8 Hz, *ortho*-pyridine *),* 6.70 (1H, dd, ^3^*J* = 17.6 Hz, 10.9 Hz, CH_2_=C**H**-)*,* 5.82 (1H, app d, ^3^*J* = 17.6 Hz C**H**_2_=CH-)*,* 5.61 (2H, s, -N-C**H_2_**-C-), 5.34 (1H, app d, ^3^*J* = 10.9 Hz, C**H**_2_=CH-), 4.49 (2H, t, ^3^*J* = 6.6 Hz, -CH_2_-C**H**_2_-O-), 3.53 (2H, t, ^3^*J* = 6.6 Hz, -C**H**_2_-CH_2_-O-), 2.46 (3H, s, -N=C-C**H**_3_).

**^13^C-NMR** (CDCl_3_, 101 MHz) *δ* 165.97, 157.55, 150.62, 144.49, 141.92, 136.64, 135.75, 134.81, 132.30, 129.85, 129.70, 129.60, 128.90, 128.85, 128.81, 128.31, 128.28, 128.22, 127.02, 126.99, 125.95, 121.41, 121.35, 117.60, 116.50, 63.12, 62.68, 51.76, 24.24, 23.60.

**ESI-MS** [M+H]^+^ *m*/*z* calculated for C_26_H_24_N_4_O_2_: 425.20, found: 425.20.

**IR** *ν* (cm^−1^) 2957.8 (vw), 2920.8 (vw), 1711.3 (vs), 1651.7 (w), 1629.1 (w), 1604.4 (m), 1575.6 (m), 1497.4 (w), 1481.0 (w), 1448.1 (m), 1404.9 (w), 1374.0 (w), 1357.6 (w), 1334.9 (w), 1310.2 (w), 1267.1 (vs), 1203.3 (w), 1178.6 (s), 1162.2 (w), 1102.5 (vs), 1073.7 (m), 1036.7 (m), 1016.1 (m), 995.5 (m), 917.4 (w), 859.8 (s), 802.2 (m), 781.6 (s), 746.7 (m), 724.0 (vs), 713.7 (vs), 695.2 (s), 635.6 (w), 619.1 (w), 580.0 (w), 555.4 (w), 541.0 (m), 508.1 (w), 493.7 (w), 483.4 (w), 456.6 (m), 401.1 (w).

^1^H-NMR, ^13^C-NMR, IR spectra are shown in the Supporting Information: Figures S6, S22, S32, respectively. 

#### 3.2.4. 2-(((2-(1-benzyl-4-(6-methylpyridin-2-yl)-1H-1,2,3-triazol-5-yl)ethoxy)carbonyl)­amino)ethyl acrylate (**4d**)

In a flame-dried 50 mL two-neck round bottom flask, 25 mL of DCM was added under argon, followed by the addition of **3b** (1.02 mmol, 0.3 g, 1 equiv.), slow addition of 2-isocyanatoethyl acrylate (0.20 mL, 1.53 mmol, 215.8 mg, 1.5 equiv.) and one drop of dibutyltin dilaurate (DBTDL). The reaction mixture was allowed to stir at room temperature for 48 h. After the removal of the solvent under reduced pressure, the residue was purified by column chromatography on silica gel using a mixture of DCM and methanol (*R*_f_ = 0.35 in 5% v/v of methanol). The product was isolated as a colourless viscous liquid with a yield of 62.7%.

**^1^H-NMR** (CDCl_3_, 400 MHz) *δ* (ppm): 8.03 (1H, d, ^3^*J* = 7.8 Hz, *ortho*-pyridine), 7.64 (1H, t, ^3^*J* = 7.8 Hz, *para*-pyridine), 7.38–7.30 (3H, m, -*meta*- and *para*-benzyl), 7.27–7.23 (2H, m, *ortho*-benzyl), 7.04 (1H, d, ^3^*J* = 7.8 Hz, CH_3_-C=C**H**-), 6.43 (1H, dd, ^3^*J* = 17.3 Hz, ^2^*J* = 1.4 Hz O=C-CH=C**H**_2_), 6.13 (1H, dd, ^3^*J* = 17.3 Hz, ^2^*J* =10.4 Hz, O=C-C**H**=CH_2_), 5.88 (1H, dd, ^3^*J* = 10.4 Hz, ^2^*J* = 1.4 Hz O=C-CH=C**H**_2_), 5.62 (2H, s, -N-C**H**_2_-), 4.55 (1H, br, -N**H**-), 4.33 (2H, ^3^*J* = 6.5 Hz, -O-C**H_2_**-C**H**_2_-C-), 4.19 (2H, t, ^3^*J* = 5.3 Hz, -O-C**H**_2_-CH_2_-NH-), 3.42 (2H, m, -O-CH_2_-C**H**_2_-NH- and -O-CH_2_-C**H**_2_-C-), 2.51 (2H, s, -N=C-C**H**_3_). 

**^13^C-NMR** (CDCl_3_, 101 MHz) *δ* 166.07, 157.75, 156.25, 150.80, 144.29, 136.89, 135.40, 133.01, 131.46, 128.95, 128.21, 128.00, 127.26, 121.56, 117.77, 63.42, 63.20, 51.66, 40.12, 24.46, 24.20.

**ESI-MS** [M+H]^+^ *m*/*z* calculated for C_23_H_25_N_5_O_4_: 436.20, found: 436.20.

**IR** *ν* (cm^−1^) 3346.6 (vw), 2959.9 (w), 2927.0 (w), 1715.5 (vs), 1602.3 (m), 1575.6 (m), 1530.3 (m), 1497.4 (w), 1483.0 (m), 1450.1 (s), 1406.9 (s), 1374.0 (w), 1359.6 (w), 1332.9 (w), 1267.1 (vs), 1180.7 (vs), 1156.0 (s), 1102.5 (s), 1071.6 (s), 1044.9 (s), 1016.1 (m), 985.3 (s), 919.4 (w), 859.8 (m), 804.3 (s), 779.6 (m), 746.7 (m), 724.0 (vs), 695.2 (s), 674.7 (m), 617.1 (w), 578.0 (m), 555.4 (m), 541.0 (m), 483.4 (m), 456.6 (m), 417.6 (w), 409.3 (w).

^1^H-NMR, ^13^C-NMR, IR spectra are shown in the Supporting Information: Figures S7, S23, S33, respectively. 

#### 3.2.5. Heteroleptic Cu(I) Complexes **5a**, **5b**, **5c**, **5d**

In a typical procedure, into a flame-dried 50 mL two-neck round bottom flask, 25 mL of DCM was added under argon, followed by the addition of tetrakis(acetonitrile)copper(I) tetrafluoroborate (0.04 mmol, 12.6 mg, 1 equiv.) (synthesised according to the literature [66]) and diphenyl phosphine ether (0.04 mmol, 21.5 mg, 1 equiv.). After 30 min under stirring, the ligands **3a, 3b, 4c, or 4d** (0.04 mmol, 1 equiv.) were added. The reaction mixture was allowed to stir at room temperature for 48 h. After the removal of the solvent under reduced pressure, the residue was dissolved in 1 mL of DCM and cyclohexane was added dropwise. The biphasic solution obtained was then left in the fridge for 48 h until a precipitated was formed. The precipitate was then collected by filtration and washed with cold cyclohexane. The product was isolated as a white (**5d**) yellowish (**5b** and **5c**) or brown solid (**5a**), yields are reported in Table 7.

**5a:**

**^1^H-NMR** (CD_2_Cl_2_, 400 MHz) *δ* 7.89 (2H, m, *para-,ortho*-pyridine ), 7.49–6.63 (34H, m, Ar), 5.74 (2H, s, N-C**H**_2_-C-), 4.79 (2H, s, -C-C**H**_2_-OH), 3.76 (1H, br, -N-CH_2_-O**H**), 2.04 (3H, s, -N=C-C**H**_3_).

**^13^C-NMR** (CDCl_3_, 101 MHz) *δ* 158.97, 158.55, 158.49, 158.43, 146.85, 143.78, 143.76, 139.08, 138.80, 135.46, 134.53, 134.44, 131.85, 130.92, 130.35, 129.73, 129.04, 128.53, 128.00, 127.79, 125.00, 124.98, 124.95, 124.52, 124.37, 124.23, 120.37, 120.06, 77.48, 77.16, 76.84, 52.93, 51.90, 25.29.

**ESI** [M]^+^ *m*/*z* calculated for C_52_H_44_CuN_4_O_2_P_2_: 881.22, found: 881.22 .

**IR** *ν* (cm^−1^) 3346.6 (vw), 2959.9 (w), 2927.0 (w), 1715.5 (vs), 1602.3 (m), 1575.6 (m), 1530.3 (m), 1497.4 (w), 1483.0 (m), 1450.1 (s), 1406.9 (s), 1374.0 (w), 1359.6 (w), 1332.9 (w), 1267.1 (vs), 1180.7 (vs), 1156.0 (s), 1102.5 (s), 1071.6 (s), 1044.9 (s), 1016.1 (m), 985.3 (s), 919.4 (w), 859.8 (m), 804.3 (s), 779.6 (m), 746.7 (m), 724.0 (vs), 695.2 (s), 674.7 (m), 617.1 (w), 578.0 (m), 555.4 (m), 541.0 (m), 483.4 (m), 456.6 (m), 417.6 (w), 409.3 (w) 

**^19^F NMR** (CDCl_3_, 377 MHz) *δ* −153.15 

**^31^P NMR** (CDCl_3_, 162 MHz) *δ* −13.90

**^11^B NMR** (CDCl_3_, 128 MHz) *δ* −0.81

^1^H-NMR, ^13^C-NMR, IR spectra are shown in the Supporting Information: Figures S8, S24, S34, respectively. 

Emission and excitation spectra in Ar-saturated DCM are shown in Figure S40.

**5b:**

**^1^H-NMR** (CDCl_3_, 400 MHz) *δ* 7.94 (1H, t, ^3^*J* = 7.8 Hz, -CH=C**H**-CH-, *para* pyridine), 7.76 (1H, d, ^3^*J* = 7.8 Hz, -C**H**=C-CH_3_, *meta*-pyridine), 7.49 (4H, br, *para*-phenyl), 7.41–7.01 (21H, br, *meta*-phenyl, *ortho*-phenyl*, para*-benzyl), 7.01–6.91 (4H, br, *meta*- and *ortho*-benzyl), 6.81–6.66 (5H, br, -N=C-C**H**, -O-C-C**H**=CH-), 5.66 (2H, s, -C-C**H**_2_-N), 3.66 (2H, m, -CH_2_-C**H**_2_-OH), 3.50 (1H, br, -CH_2_-O**H)** 3.15 (2H, t, ^3^*J* = 6.9 Hz, -C**H**_2_-CH_2_-OH), 2.02 (3H, s, N=C-C**H_3_**).

**^13^C-NMR** (CDCl_3_, 101 MHz) *δ* 159.05, 158.64, 158.58, 158.52, 147.11, 142.84, 139.43, 134.67, 134.60, 134.32, 131.83, 129.13, 128.59, 127.67, 124.99, 124.65, 124.51, 124.38, 120.40, 118.76, 77.48, 77.16, 76.84, 59.91, 52.65, 27.05, 25.42.

**IR** *ν* (cm^−1^) 3533.8 (vw), 3060.7 (vw), 2922.9 (w), 2859.1 (w), 1631.1 (vw), 1608.5 (w), 1573.5 (w), 1483.0 (w), 1458.3 (m), 1433.7 (vs), 1258.8 (w), 1215.6 (s), 1094.3 (s), 1053.1 (vs), 997.6 (s), 878.3 (w), 804.3 (m), 744.6 (vs), 724.0 (s), 693.2 (vs), 520.4 (s), 508.1 (vs), 483.4 (s), 475.1 (vs), 466.9 (s), 419.6 (s), 411.4 (s) 

**ESI** [M]^+^ *m*/*z* calculated for C_53_H_46_CuN_4_O_2_P_2_: 895.24 , found: 895.24 .

**^19^F NMR** (CDCl_3_, 377 MHz) *δ* −153.16

**^31^P NMR** (CDCl_3_, 162 MHz) *δ* −13.91

**^11^B NMR** (CDCl_3_, 128 MHz) *δ* −0.81

**Crystal data** for **5b:** C_55_H_50_BCl_4_CuF_4_N_4_O_2_P_2_, 1153.08 g mol^−1^, orthorhombic, *Pbca, a* = 26.5017(15), *b* = 12.1623(4), *c* = 33.0708(11) Å, *Z* = 8, *V* = 10659.4(8) Å^3^, T = 100(2) K, d_calc_ = 1.437 g cm^-3^, *F*(000) = 4736, μ(Mo-*K*α) = 0.730 mm^−1^. Colourless rod, 0.42×0.17×0.05 mm^3^, 24885 data, 2θ_max_ = 52.74°, 10557 unique (*R*_int_ = 0.0524), 660 parameters, final *wR*_2_ = 0.2082, *S* = 1.008 (all data), *R*_1_ (6502 data with I > 2σ(I)) = 0.0686, max. diff. peak/hole +0.93/-0.64 eÅ^-3^. Full crystallographic data for the structure have been deposited with the Cambridge Crystallographic Data Centre as CCDC 2067971 (copies available free of charge from https://www.ccdc.cam.ac.uk/structures/, accessed on 1 March 2021)

^1^H-NMR, ^13^C-NMR, IR spectra are shown in the Supporting Information: Figures S9, S25 and S35, respectively.

Emission and excitation spectra in Ar-saturated DCM are shown in Figure S41.

**5c:**

**^1^H-NMR** (CDCl_3_, 400 MHz) *δ* (ppm): 7.96 (1H, d, ^3^*J* = 7.8 Hz, *ortho*-pyridine), 7.89 (1H, d, ^3^*J* = 7.8 Hz, *para*-pyridine), 7.74 (2H, d, ^3^*J* = 8.1 Hz, *meta*-styrene), 7.58-6.89 (29 H, br, phenyl, *ortho- and meta*-benzyl, *ortho*-styrene, *ortho*-O-Ar), 6.80-6.63 (8H, br, *ortho*-pyridine, *meta*-O-Ar, *para*-O-Ar*, *-C**H**=CH_2_) 5.80 (1H, app d, ^3^*J* = 17.6 Hz C**H**_2_=CH-), 5.73 (2H, s, -N-C**H_2_**-C-), 5.36 (1H, app d, ^3^*J* = 10.9 Hz C**H**_2_=CH-), 4.25 (2H, t, ^3^*J* = 6.9 Hz, -O-**CH_2_**-CH_2_-), 3.47 (2H, t, ^3^*J* = 6.9 Hz, CH_2_-C**H_2_**_-_C), 1.99 (3H, s, N-C-C**H_3_**).

**^13^C-NMR** (CDCl_3_, 101 MHz) *δ* 166.15, 159.45, 146.66, 142.32, 139.37, 135.95, 134.53, 131.90, 130.04, 129.28, 128.82, 127.53, 126.24, 125.04, 124.77, 118.65, 61.37, 52.86, 25.41, 22.77. 

**IR** *ν* (cm^−1^) 3398.0 (vw), 3048.3 (vw), 2961.9 (vw), 2927.0 (vw), 2855.0 (vw), 1717.5 (w), 1604.4 (w), 1565.3 (w), 1478.9 (w), 1460.4 (m), 1433.7 (vs), 1380.2 (w), 1267.1 (m), 1215.6 (m), 1180.7 (w), 1160.1 (w), 1094.3 (s), 1053.1 (vs), 1034.6 (vs), 997.6 (s), 870.1 (w), 802.2 (m), 744.6 (vs), 724.0 (s), 693.2 (vs), 619.1 (w), 580.0 (w), 543.0 (m), 510.1 (vs), 485.4 (s), 475.1 (s), 466.9 (s), 450.5 (m), 438.1 (m), 419.6 (s) cm-1

**ESI** [M]^+^ *m*/*z* calculated for C_62_H_52_CuN_4_O_3_P_2_ : 1025.28, found: 1025.28.

**^19^F NMR** (CDCl_3_, 377 MHz) *δ* −153.04

**^31^P NMR** (CDCl_3_, 162 MHz) *δ* −13.93

**^11^B NMR** (CDCl_3_, 128 MHz) *δ* −1.11

^1^H-NMR, ^13^C-NMR, IR spectra are shown in the Supporting Information: Figures S10, S26 and S36, respectively.

Emission and excitation spectra in Ar-saturated DCM are shown in Figure S42.

**5d:**

**^1^H-NMR** (CDCl_3_, 400 MHz) *δ* (ppm): 7.92 (1H, t ^3^*J* = 7.8 Hz, *para*-pyridine), 7.70 (1H, d, ^3^*J* = 7.8 Hz, *ortho*-pyridine), 7.58–6.93 (29H, br, phenyl*,* benzyl, *meta*-O-Ar, *para*-O-Ar), 6.85–6.74 (3H, br, *ortho*-O-Ar, *ortho*-pyridine) 6.74–6.68 (2H, br, *meta*-O-Ar) 6.37 (1H, dd, ^3^*J* = 17.2 Hz, ^2^*J* = 1.4 Hz C**H**_2_=CH-), 6.11 (1H, app d, ^3^*J* = 17.2 Hz, ^2^*J* = 10.4 Hz CH_2_=C**H**-), 5.82 (1H, dd, ^3^*J* = 10.4 Hz, ^2^*J* = 1.4 Hz C**H**_2_=CH-) 5.69 (2H, s, -N-C**H_2_**-C-), 5.25 (1H, br, C-N**H**-CH_2_-), 4.15 (2H, t, ^3^*J* = 6.9 Hz, -O-**CH_2_**-CH_2_-NH-), 3.97 (2H, t, ^3^*J* = 6.9 Hz, -C-CH_2_-C**H_2_**_-_O-), 3.37 (2H, m, -O-**C**H**_2_**-C**H**_2_-NH-), 3.21 (2H, t, ^3^*J* = 6.9 Hz, -C-C**H**_2_-CH**_2_**_-_O-), 2.06 (3H, s, N-C-C**H_3_**).

**^13^C-NMR** (CD_2_Cl_2_, 101 MHz) *δ* 166.49, 160.31, 159.03, 158.97, 158.91, 156.40, 147.07, 139.46, 134.98, 134.93, 134.65, 134.16, 133.24, 132.44, 131.38, 129.68, 128.53, 127.89, 125.44, 125.42, 125.29, 124.71, 124.57, 124.42, 120.91, 118.61, 63.73, 61.61, 54.54, 54.27, 54.00, 53.73, 53.46, 53.29, 40.63, 27.47, 25.78.

**IR** *ν* (cm^−1^) 3398.0 (vw), 3048.3 (vw), 2961.9 (vw), 2927.0 (vw), 2855.0 (vw), 1717.5 (w), 1604.4 (w), 1565.3 (w), 1478.9 (w), 1460.4 (m), 1433.7 (vs), 1380.2 (w), 1267.1 (m), 1215.6 (m), 1180.7 (w), 1160.1 (w), 1094.3 (s), 1053.1 (vs), 1034.6 (vs), 997.6 (s), 870.1 (w), 802.2 (m), 744.6 (vs), 724.0 (s), 693.2 (vs), 619.1 (w), 580.0 (w), 543.0 (m), 510.1 (vs), 485.4 (s), 475.1 (s), 466.9 (s), 450.5 (m), 438.1 (m), 419.6 (s) 

**ESI** [M]^+^ *m*/*z* calculated for C_59_H_53_CuN_5_O_5_P_2_: 1036.28, found: 1036.28 .

**^19^F NMR** (CDCl_3_, 377 MHz) *δ* –153.20

**^31^P NMR** (CDCl_3_, 162 MHz) *δ* –13.84

**^11^B NMR** (CDCl_3_, 128 MHz) *δ* –0.79

^1^H-NMR, ^13^C-NMR, IR spectra are shown in the Supporting Information: Figures S11, S27, S37, respectively.

Emission and excitation spectra in Ar-saturated DCM are shown in Figure S43.

#### 3.2.6. Synthesis of Poly(methyl acrylate) Homopolymer PMA

In a flame-dried 25 mL two-neck round bottom flask, 5 mL of toluene was added followed by the addition of methyl acrylate (MA, 0.50 mL, 5.51 mmol, 0.475 g, 100 equiv.), and 2,2′-azobis(2-methylpropionitrile) (AIBN, 0.055 mmol, 9.05 mg, 1 equiv.). Argon was bubbled through the solution for 5 min and then the reaction mixture was allowed to stir at 70 °C for 4 h under an argon blanket. The reaction was then cooled and quenched by being opened to air and the polymer was then precipitated by dropwise addition into 15 mL of stirring cold hexane. The product was dried and a transparent solid was obtained with a yield of 83.4% (*M*_n_ = 11.71 kg/mol, *Ð* = 2.46).

^1^H-NMR spectrum and SEC chromatogram are shown in the Supporting Information: Figures S12, S38, respectively.

#### 3.2.7. Synthesis of Poly(styrene) Homopolymer PS

In a flame-dried 25 mL two-neck round bottom flask, 5 mL of toluene was added followed by the addition of styrene (Sty, 0.57 g, 0.63 mL, 5.51 mmol, 100 equiv.), and 2,2′-azobis(2-methylpropionitrile) (AIBN, 0.055 mmol, 9.05 mg, 1 equiv.). Argon was bubbled through the solution for 5 min and then the reaction mixture was allowed to stir at 70 °C for 4 h under an argon blanket. The reaction was then cooled and quenched by opening it to air and the polymer was then precipitated by dropwise addition into 15 mL of stirring cold hexane. The product was dried and a transparent solid was obtained with a yield of 57.8% (*M*_n_ = 14.5 kg/mol, *Ð* = 1.44).

^1^H-NMR spectrum and SEC chromatogram are shown in the Supporting Information: Figures S13, S39, respectively.

#### 3.2.8. Synthesis of Poly(Sty-co-4c): Copolymer **6c**

In a flame-dried 25 mL two-neck round bottom flask, 430 µL of toluene was added, followed by the addition of styrene (St, 52.63 µL, 0.46 mmol, 47.8 mg, 10 equiv.), M2 (20.0 mg, 0.046 mmol, 1 equiv.) and AIBN (0.003 mmol, 0.42 mg, 0.07 equiv.). Argon was bubbled through the solution for 5 min and then the reaction mixture was allowed to stir at 70 °C for 24 h under an argon blanket. The polymer was then precipitated in 1.5 mL of cold hexane. The product was isolated as a yellowish solid at a yield of 64.4% (M_n_ = 13.5 g/mol, Ð = 1.65). The monomer ratio in the copolymer was determined by ^1^H-NMR spectroscopy and found to correspond to 8% **4c**. 

^1^H-NMR spectrum shown in the Supporting Information: Figure S14.

#### 3.2.9. Synthesis of Poly(MA-co-4d): Copolymer **6d**

In a flame-dried 25 mL two-neck round bottom flask, 403 µL of toluene was added, followed by the addition of MA (39.83 µL, 0.44 mmol, 37.8 mg, 10 equiv.), **4d** (20.0 mg, 0.044 mmol, 1 equiv.) and AIBN (0.002 mmol, 0.40 mg, 0.06 equiv.). Argon was bubbled through the solution for 5 min and then the reaction mixture was allowed to stir at 70 °C for 24 h. The polymer was then precipitated in 1.5 mL of cold hexane. The product was isolated as a transparent solid at a yield of 83.4% (*M*_n_ = 9.54 kg/mol, *Ð* = 1.92). The monomer ratio in the copolymer was determined by ^1^H-NMR spectroscopy and found to correspond to 11% **4c**.

^1^H-NMR spectrum is shown in the Supporting Information: Figure S15.

#### 3.2.10. Post-Polymerisation Complexation to Obtain Functional Copolymers **7c** and **7d**

In a typical complexation, in a flame-dried 25 mL two-neck round bottom flask, 10 mL of DCM was added under argon, followed by the addition of tetrakis(acetonitrile)copper(I) tetrafluoroborate (0.09 mmol, 0.048 g, 1 equiv. with respect to the functional groups in the polymer) and DPEPhos (0.09 mmol, 0.073 g, 1 equiv. with respect to the functional groups in the polymer). After 30 min under stirring, **6** (0.14 mmol, 1 equiv. with respect to the functional groups in the polymer) was added. The reaction mixture was allowed to stir at room temperature for 72 h. After removal of the solvent under reduced pressure, the residue was dissolved in 2 mL of THF and precipitated in 10 mL of cold n-hexane. After filtration, the product was obtained as a white (**7c**) or colourless (**7d**) solid.

^1^H-NMR spectra are shown in the Supporting Information: Figures S16 and S17, respectively.

## 4. Conclusions

In this work, four novel heteroleptic copper (I) complexes were successfully synthesised, two of which bear polymerisable groups: a styrenic or an acrylate moiety. The electrochemical and the photophysical investigation of the complexes were performed and showed a good correlation with the TD-DFT calculations. Overall, the complexes present reasonable quantum yields in solution and in solid state, emitting a bright cyan colour. The copolymerisation of the diimine ligands bearing the polymerisable groups and the corresponding monomers, followed by the coordination of the Cu(I)-diphosphine moiety, gave two luminescent metallopolymers. The emission quantum yields of these copolymers were compared to the corresponding polymer blends, showing the significance of the chemical environment and the final structure of the emitter. Especially the copolymer made with the acrylate unit presents the same emission behaviour as the corresponding blend. On the contrary, the copolymer made with the less emissive Cu(I) complex, the one bearing the styrenic unit, presented a notable improvement in respect to the corresponding blend. This effect was attributed to the loss of the significant electronic involvement of the double bond in the styrene, thanks to the polymerisation. 

## Figures and Tables

**Table 1:** Number average molecular weight (*M*_n_) and dispersity (*Ð*) of the copolymers obtained, determined by SEC in tetrahydrofuran (THF).

Polymer	*M*_n_ (kg/mol)	*Đ*
**6c**	13.5	1.65
**7c**	11.8	1.63
**6d**	9.54	1.92
**7d**	7.26	1.62

**Table 3:** Quantum yield of Cu(I) complexes, metallopolymers and blends in solid state at room temperature.

Sample	λ_em_ (nm)	Φ ^a^
**5a**	511	0.06 (±0.02)
**5b**	504	0.12 (±0.02)
**5c**	501	0.09 ^b^ (±0.12)
**5d**	503	0.17 (±0.01)
**7c**	508	0.09 (±0.01)
**7d**	503	0.10 (±0.01)
PS/**5c**	n.d.	n.d.
PMA/**5c**	n.d.	n.d.
PS/**5d**	502	0.13 (±0.02)
PMA/**5d**	507	0.21 (±0.01)

^a^ determined with an integrating sphere for λ_ex_ = 350 nm; ^b^ a statistical error associated with these measurements was attributed to the granular form of the sample [63].

**Table 4:** Electrochemical properties of complexes reported versus Fc/Fc+ ^a^.

Sample	E_ox_/V	E_red_1/V	E_red_2/V	Δ_HOMO-LUMO_/eV	Δ_HOMO-LUMO_^c^/eV
**5a**	n.a.; 0.84 ^b^	n.a.	n.a.	n.a.	n.a.
**5b**	0.68; 0.82 ^b^	−2.47	−2.84	3.15	4.35
**5c**	0.68; 0.84 ^b^	−2.33	−2.70	3.01	4.17
**5d**	0.64; 0.88 ^b^	−2.48	−2.94	3.12	4.37

^a^ in DMF (0.1 M TBAPF_6_) unless otherwise noted; ^b^ in DCM (0.1 M TBAPF_6_); ^c^ HOMO and LUMO energies of complexes optimised at PBE0/6-31G**/LANL2DZ/Grimme dispersion level (vacuum).

**Table 5:** Quantities of reagents used for the synthesis of **2a** and **2b** and yields.

Compound	Alcohol	Quantity (g)	Yield (%)
**2a**	**8a**	0.98	71.3
**2b**	**8b**	1.22	72.5

**Table 6:** Synthetic yields of **3a** and **3b**.

Compound	Yield (%)
**3a**	52.3
**3b**	64.3

**Table 7:** Synthetic yields of **5a**, **5b**, **5c** and **5d**.

Product	Yield (%)
**5a**	63.6
**5b**	68.3
**5c**	59.2
**5d**	65.7

## Data Availability

Datasets related to this article can be provided upon request.

## Supplementary Materials

The following are available online. Details regarding the instrumentation used in this work and supporting data, and cif. and checkcif. Files.
