# Supplementary material for: Cyan-Emitting Cu(I) Complexes and Their Luminescent Metallopolymers
Source: Molecules. 2021 Apr 28;26(9):2567. doi: 10.3390/molecules26092567 (PMC8125312; doi:10.3390/molecules26092567)
Supplement: Supplementary file 1 [file molecules-26-02567-s001.zip › molecules-1170601-supplementary.pdf]

molecules

SUPPORTING INFORMATION

Cyan-emitting Cu(I) complexes and their luminescent metallopolymers

F. Ferrari[1], J. Braun[2], C. E. Anson[2], B. D. Wilts[3], D. Moatsou[1*], C. Bizzarri[1*]

[1] Karlsruhe Institute of Technology, Institute of Organic Chemistry, Fritz-Haber-Weg 6, 76131, Karlsruhe, Germany
[2] Karlsruhe Institute of Technology, Institute of Inorganic Chemistry, Engesserstrasse 15, 76131, Karlsruhe, Germany
[3] University of Fribourg, Adolphe Merkle Institute, Chemin des Verdiers 4, 1700, Fribourg, Switzerland.
* Correspondence: dafni.moatsou@kit.edu (DM), claudia.bizzarri@kit.edu (CB)

Contents

Instrumentation 2
Nuclear Magnetic Resonance (NMR) Spectroscopy 2
Mass Spectrometry (MS) 2
Size exclusion chromatography 2
Photophysics 3
Solid state quantum yield measurements 3
Electrochemistry 5
Time-Dependent Density Fourier Transform 5
X-ray Crystallography 5
Atomic Force Microscopy (AFM) 5
Optical Microscopy 5
Supporting data 6
^{1}H NMR Spectra 6
^{13}C NMR spectra 17
FTIR spectra 23
SEC Chromatograms 29
Photophysical data 30
Computational data 32
Electrochemical data 33
Photophysical analysis in solid state 36
Atomic Force Microscopy measurements 43
Optical microscopy 45

Instrumentation

Nuclear Magnetic Resonance (NMR) Spectroscopy

NMR spectra of compounds were recorded on a Bruker Avance 400 NMR instrument at 400 MHz for ^{1}H NMR and 101 MHz for ^{13}C NMR. All NMR spectra were obtained at room temperature. The chemical shift is displayed in parts per million (ppm) using the residual solvent peak for reference: in deuterated chloroform 7.26 ppm for ^{1}H and 77.0 ppm for ^{13}C. When reporting couplings, the following abbreviations are used: s = singlet, d = doublet, t = triplet, m = multiplet, dd = doublet of doublet, ddd = doublet of doublet of doublet, app d= apparent doublet, br = broad. Coupling constants, *J*, are given in Hertz (Hz) with the largest value first. Couplings are given with their respective number of bindings and binding partners, as far as they could be determined, written as index of the coupling constants.

Mass Spectrometry (MS)

Electrospray ionization (ESI) mass spectra were recorded on a Q-Tof Premier TM spectrometer from Waters-Micromass. The mass to charge ratio (*m*/*z*) was plotted against the relative intensity, with the base peak set to 100%.

Size exclusion chromatography

An Agilent 1200 system, consisting of an autosampler, a PLgel 5 μm bead size guard column (50 × 7.5 mm), one PLgel 5 μm Mixed E column (300 × 7.5 mm), three PLgel 5 μm Mixed C columns (300 × 7.5 mm), a differential

refractive index detector and a UV detector was used for size exclusion chromatography (SEC) measurements. THF was employed as eluent at 35 °C with a flow rate of 1 mL min^{-1}. The SEC system was calibrated using a "readyCal-Kit Poly(styrene)" ranging from 370 to 2,520,000 Da. In a typical procedure, 100 µL of a 2.0 mg mL^{-1} polymer solution was injected into the system.

Fourier transform infrared spectroscopy (FTIR)

Infrared spectra of all samples were recorded on a Bruker alpha-p instrument in a frequency range of 3,997.41 to 373.828 cm^{-1} using ATR technology.

Photophysics

The instrument used was a Lambda 750 double-beam UV/Vis-NIR spectrometer. Emission and excitation spectra were recorded with a Fluoromax 4 from Horiba Jobin. All the solutions were deareated by bubbling argon inside the cuvettes, closed with a septum, for 10 minutes at least.

Lifetime experiments were performed by time-correlated single photon counting method (TCSPC) with a DeltaTime kit for Delta-Diode source on FluoroMax systems, including DeltaHub and DeltaDiode controller. NanoLED 370 was used as the excitation source (λ = 366 nm).

Solid state quantum yield measurements

The quantum yield of the solid samples was measured from thin films cast on glass substrates or powders pressed between two glass slides, using an integrating sphere (AvaSphere-50; Avantes, Apeldoorn, the Netherlands) in transmission configuration. Incident light from a high-power Xenon light source (HPX2000; OceanInsight, Orlando, FL, USA) coupled into an optical fibre was filtered through a 350 nm bandpass filter (FB350-10; Thorlabs, Newton, NJ, USA) and focussed onto the sample using a collimating lens (74-UV; OceanInsight). Light transmitted from the sample was extracted from the integrating sphere *via* an optical fibre and analysed using a diode spectrometer (Maya2000, OceanInsight). By integrating the transmission peak of an empty glass sample (S_{glass}) around the excitation wavelength (350 nm) as well as integrating the direct transmission peak (S_{sample}) and the emission spectra ($S_{emission}$) from different compounds, the quantum yield of the compounds was then derived as $\Phi = \frac{S_{emission}}{S_{glass} - S_{sample}}$ (Figure S1).

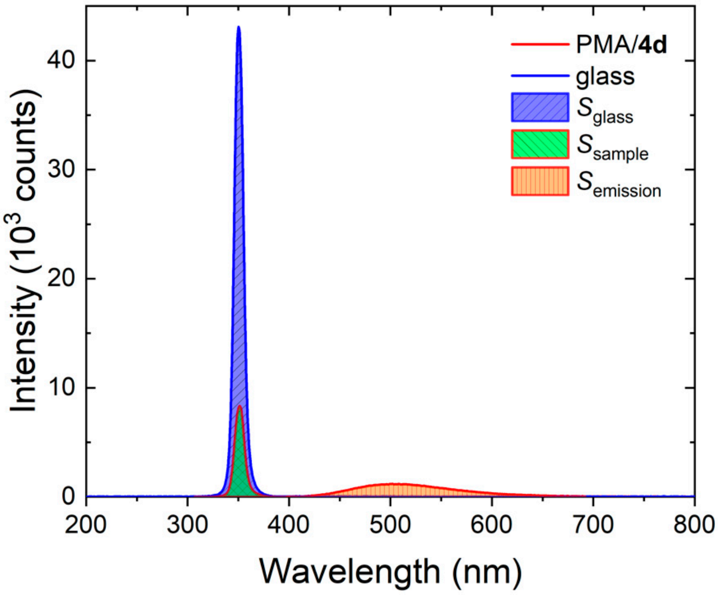

Figure S1. Combined transmission and emission spectra of PMA/**5d** and glass indicating the areas that were used for the calculation of the quantum yield Φ.

Electrochemistry

Cyclic voltammetry experiments were performed with a Gamry Interface 1010B in a three electrodes electrochemical cell. The electrochemical cell was equipped with a Glassy Carbon Disc (GCD) working electrode, Ag wire as quasi reference electrode and a Pt wire as the auxiliary electrode. All the experiments were performed in *N,N*-dimethylformamide (0.1m $TBAPF_6$) solution, under Ar atmosphere, at a sample concentration of 2mM. Ferrocene (Fc) was added after each experiment as an internal standard, according to IUPAC recommendation. The redox properties are reported versus Fc/Fc^+ couple.

Time-Dependent Density Fourier Transform

Quantum chemical calculations were performed on commission by Dr. R. Q. Albuquerque from Computational Chemistry Solutions (web: https://www.ccsol.de). A conformational analysis was carried out using the Amber force field to determine low energy conformations to be used in the quantum chemistry calculations. The geometry of complexes **5b**, **5c** and **5d** was then optimized at the Density Functional Theory (DFT) level (PBE0/6-31G**/LANL2DZ, in vacuum) using the QChem package [Y. Shao et al., Mol. Phys. 113, 184-215 (2015), DOI: 10.1080/00268976.2014.952696]. Dispersion interactions were taken into account via the Grimme dispersion correction. The optimised geometries were then used to predict absorption spectra via Time-Dependent DFT (TDDFT), from where the energy and molecular orbital compositions of triplet and singlet states were obtained.

Further details: the PBE0 is the Perdew-Burke-Ernzerhof-Adamo functional (see references : Perdew *et al. Phys. Rev. Lett.* **1996**, *77*, 3865; *Phys. Rev. Lett.* **1997**, *78*, 1396; C. Adamo *et al. J. Chem. Phys.* **1999**, *110*, 6158). This functional works very good for metal complexes and gives comparable results to get the final optimized geometry as the functional B3LYP, which is slower to converge. A direct comparison of the two functionals can be found for Ir complexes (see T. B. de Queiroz *et al. J. Phys. Chem. C* **2013**, *117*, 2966). Additionally, two basis sets have to be used. LAN2LDZ is used for the metal core, in particular treating only the valence electrons (see: P.J. Hay *et al. J. Chem. Phys* **1985**, *82*, 270). For the non-metallic atoms, the 6-31G** basis set is used (G. A. Petersson *et al. J. Chem. Phys.* **1991**, *94*, 6081).

X-ray Crystallography

A suitable crystal was coated in perfluorinated polyether oil, mounted on a Mitegen loop, and transferred to a Stoe StadiVari diffractometer where it was kept at 100 K using an Oxford Cryostream 700 cooling system. The structure was solved using SHELXT [1] and refined using SHELXL-2018[2]. Full crystallographic details for the structure have been deposited with the Cambridge Crystallographic Data Centre as supplementary publication CCDC 2067971; copies of the data can be obtained from https://www.ccdc.cam.ac.uk/structures/

[1]:G.M. Sheldrick, Acta Cryst. A71, 3-8 (2015);

[2]: G.M. Sheldrick, Acta Cryst. C71, 3-8 (2015).

Atomic Force Microscopy (AFM)

A Park NX10 atomic force microscope was used in non-contact mode (tapping) to characterise the topography of the samples. Aluminium-coated silicon probes (NanoAndMore) with a nominal force constant of 40 N m-1, a resonance frequency of 300 kHz, and a tip radius <10 nm were employed. Samples were dissolved in chloroform at 15 mg/mL and drop-cast onto glass slides. After evaporation of the solvent under ambient conditions, the surface morphology was observed in non-contact mode. One sample was scratched (**7d**) in order to determine the typical film thickness, which was found to be 1.1 μm.

Optical Microscopy

The optical microscopy images of samples were measured using a ZEISS Axio Scope.A1 optical microscope (Germany), with a Xenon lamp (SLS401, Thorlabs) and a 10× EC Epilan-Apochromat objective (Zeiss, NA = 0.3). Images were recorded with a Grasshopper 3 camera (FLIR).

Supporting data

^{1}H NMR Spectra

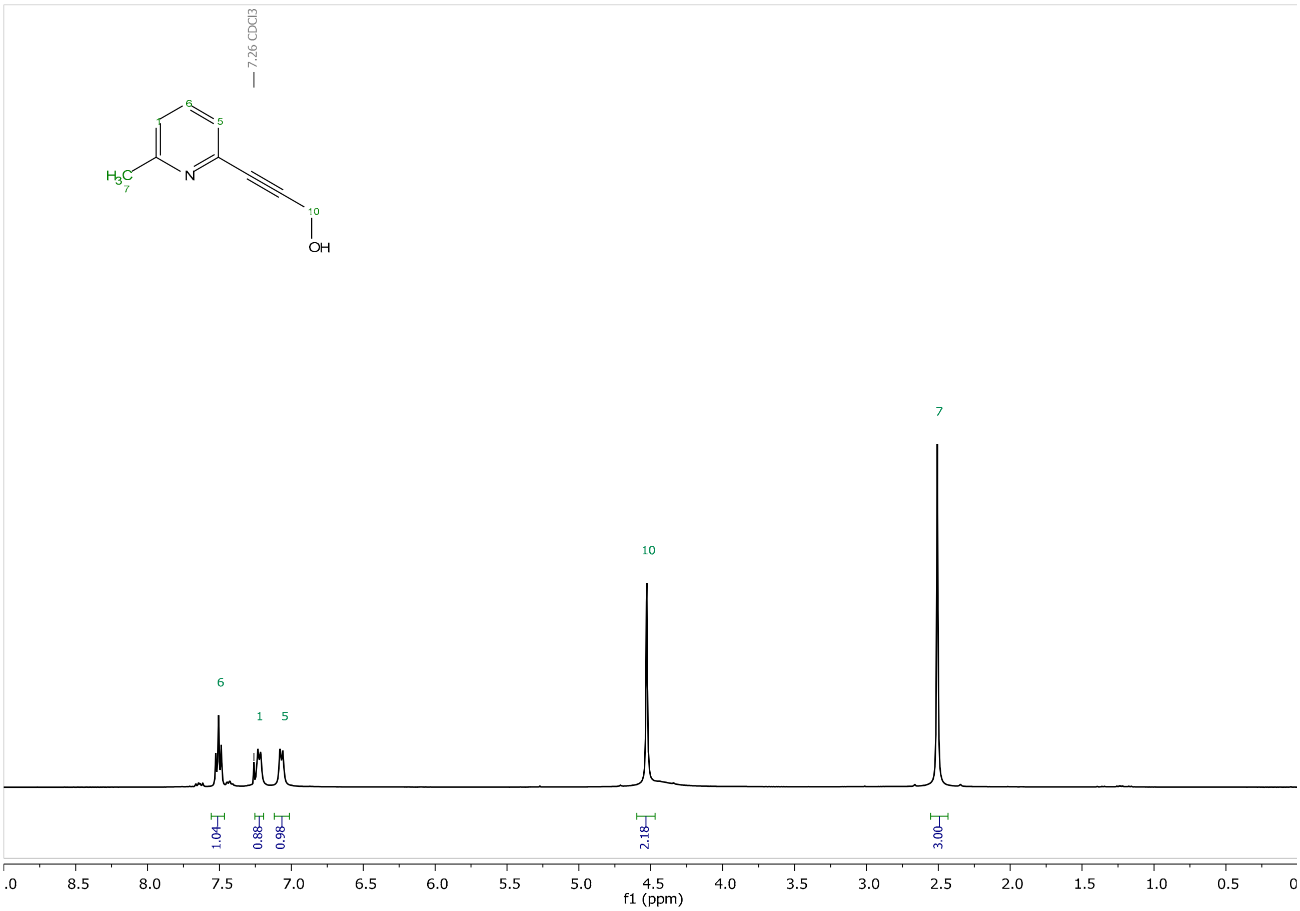

Figure S2. ^{1}H NMR spectrum of **2a** in $CDCl_3$ (400 MHz).

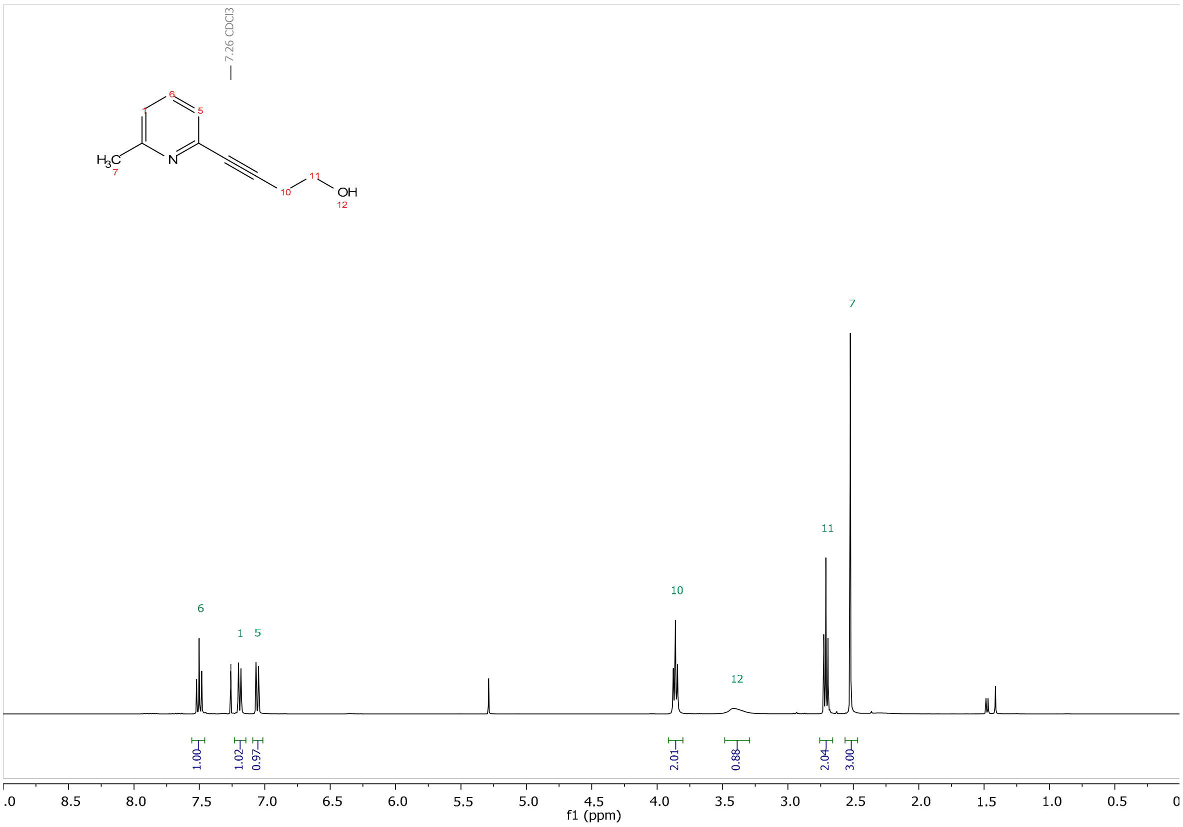

Figure S3. ^{1}H NMR spectrum of **2b** in $CDCl_3$ (400 MHz).

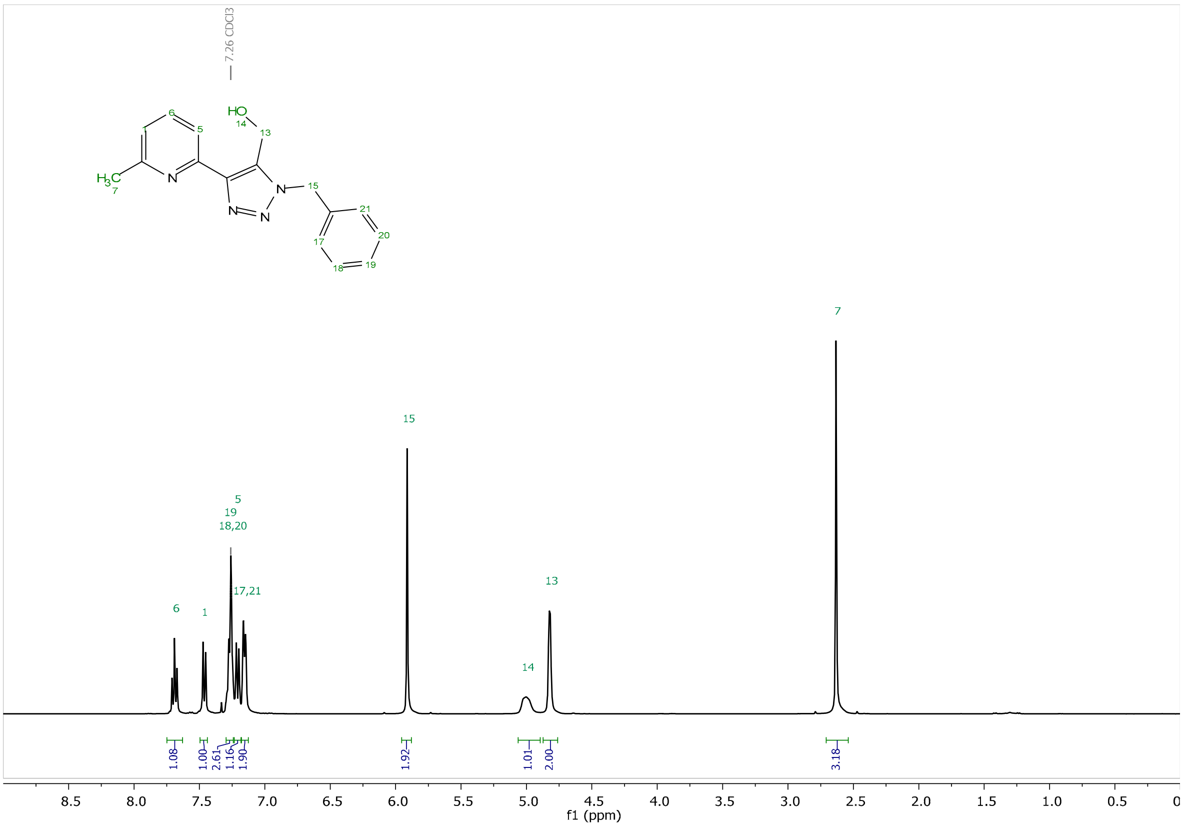

Figure S4. ^{1}H NMR spectrum of **3a** in $CDCl_3$ (400 MHz).

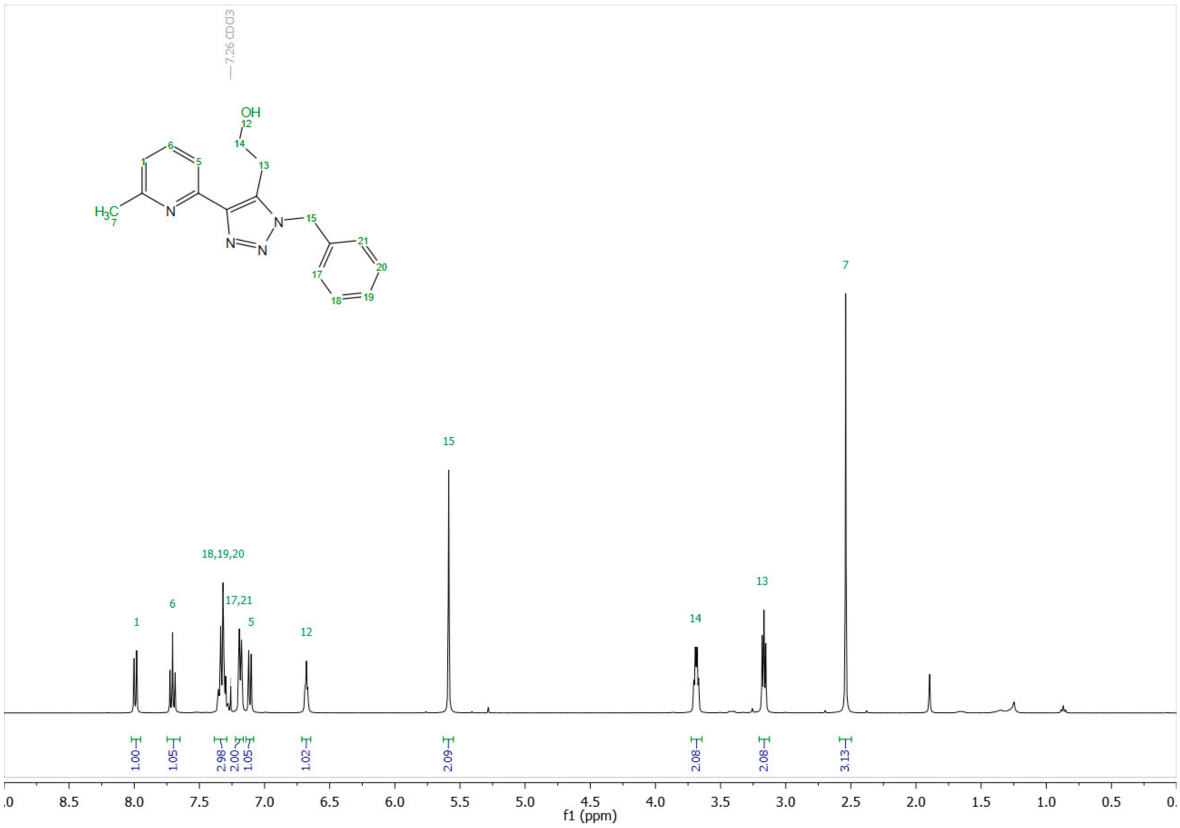

Figure S5. ^1H NMR spectrum of **3b** in $CDCl_3$ (400 MHz).

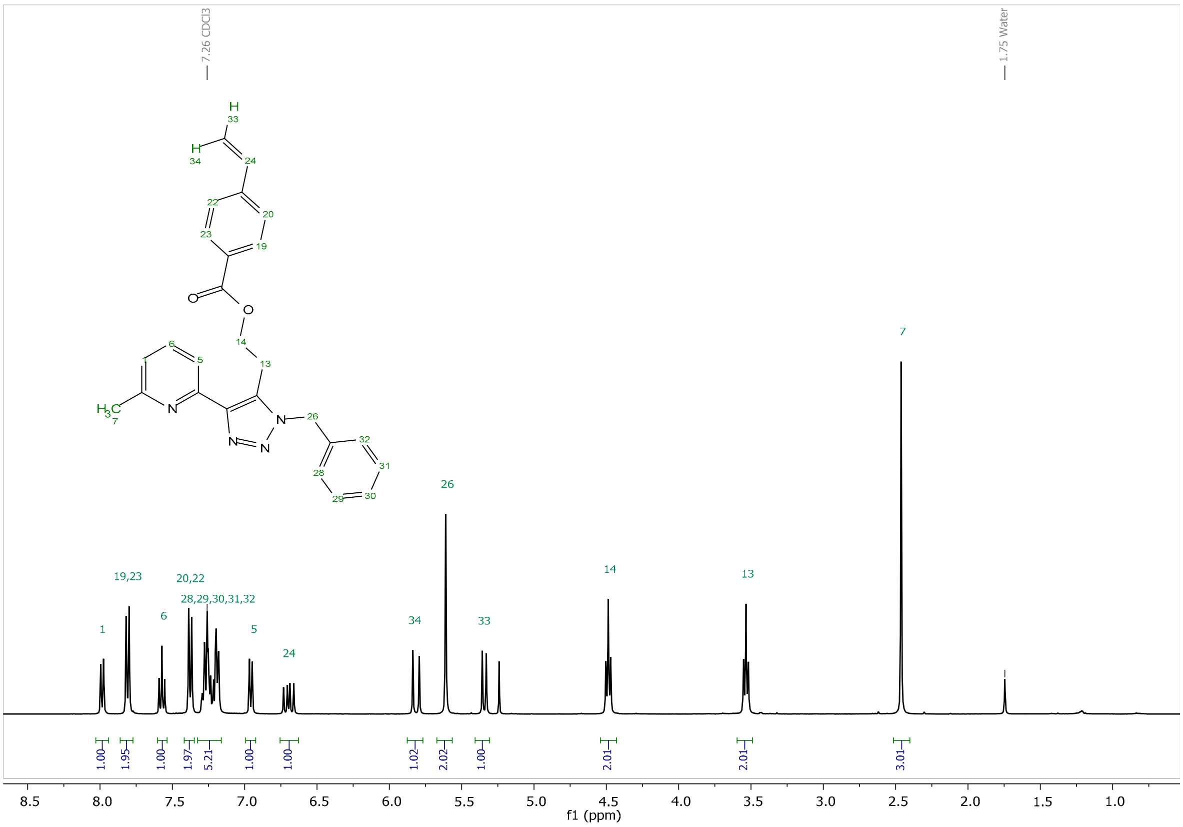

Figure S6. ^1H NMR spectrum of **4c** in $CDCl_3$ (400 MHz).

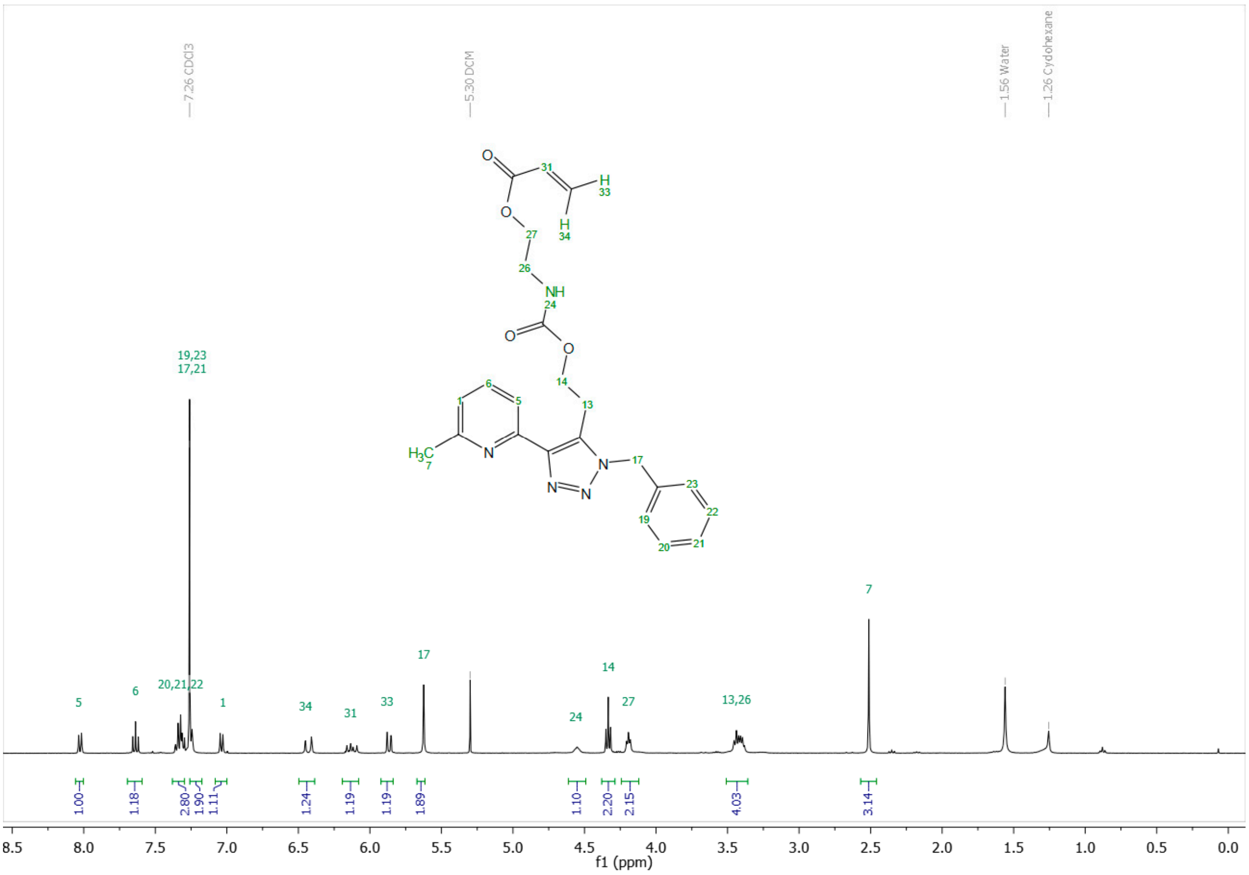

Figure S7. ^{1}H NMR spectrum of **4d** in $CDCl_3$ (400 MHz).

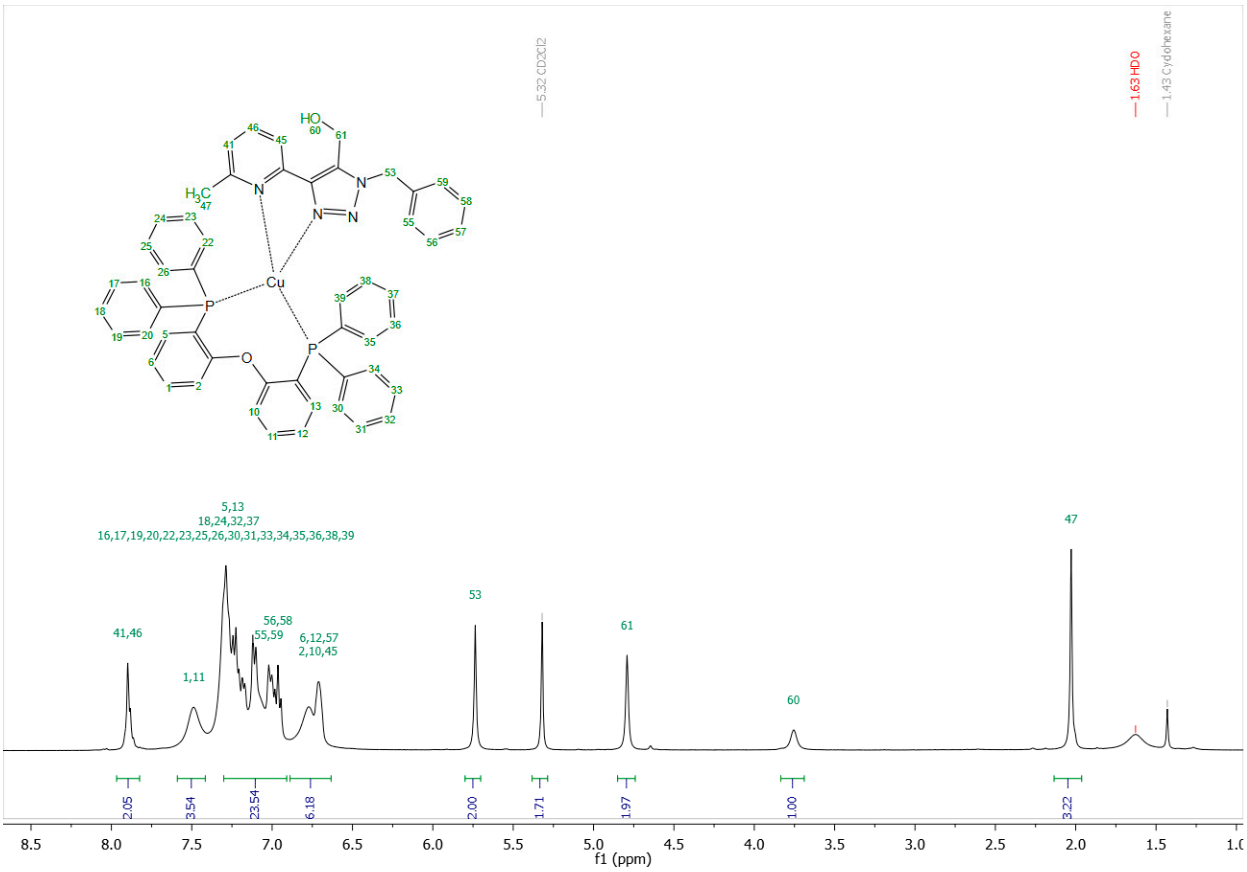

Figure S8. ^{1}H NMR spectrum of **5a** in CD_2Cl_2 (400 MHz).

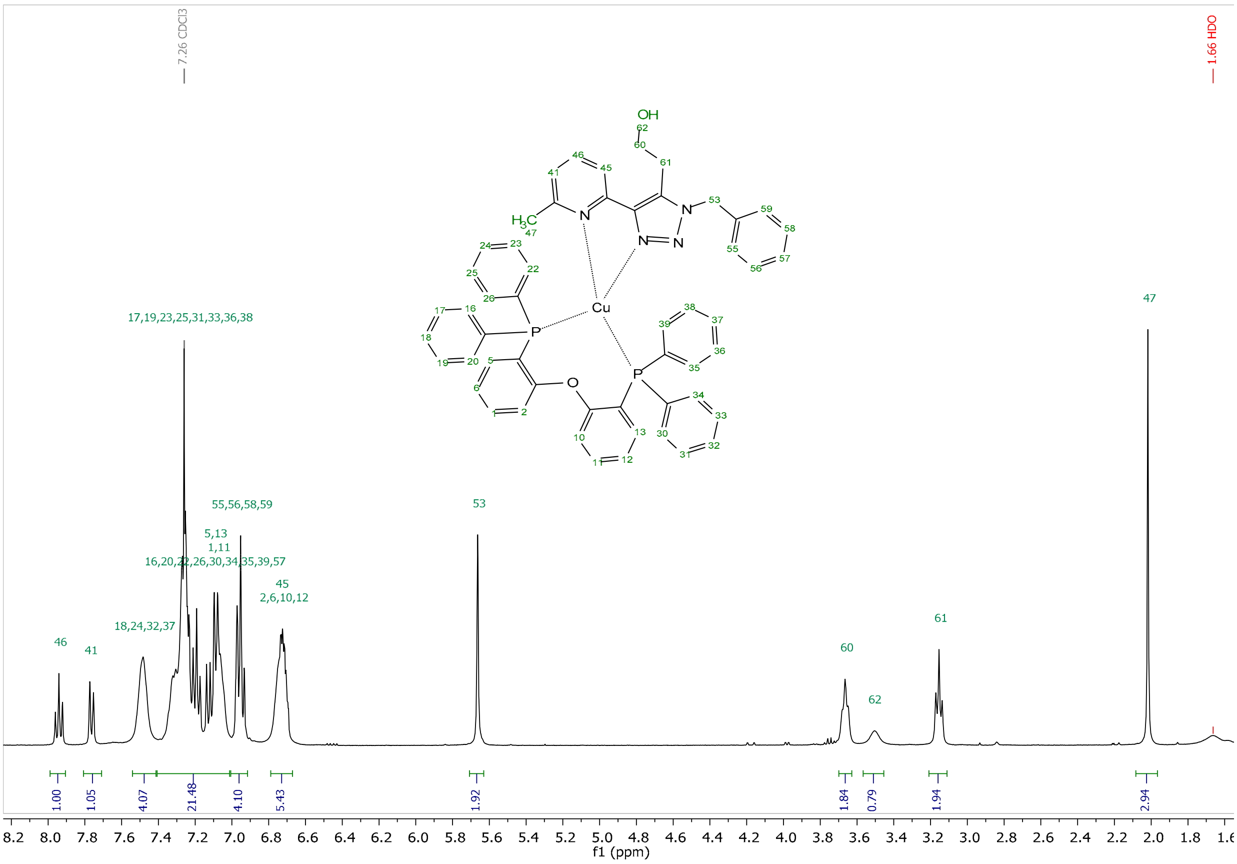

Figure S9. ^{1}H NMR spectrum of **5b** in $CDCl_3$ (400 MHz).

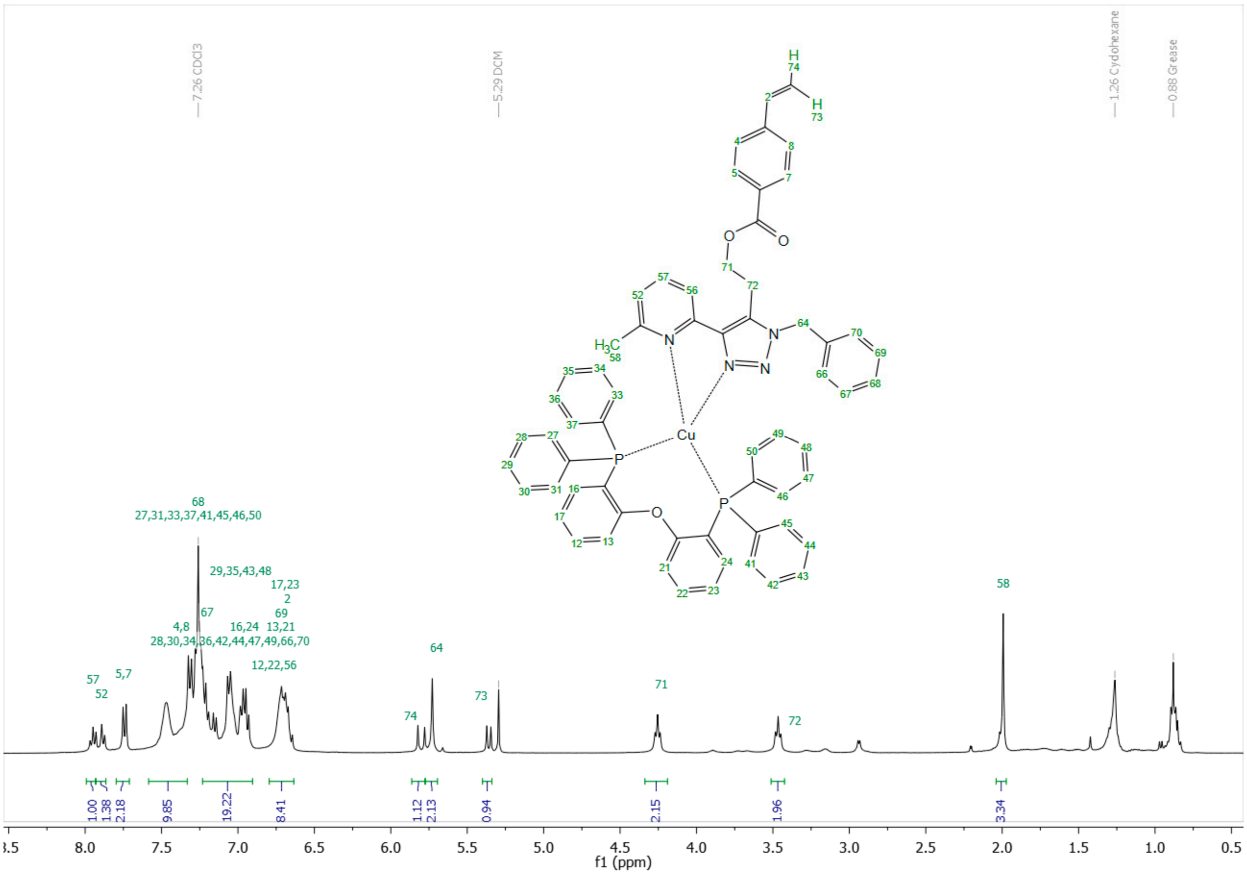

Figure S10. ^{1}H NMR spectrum of **5c** in $CDCl_3$ (400 MHz).

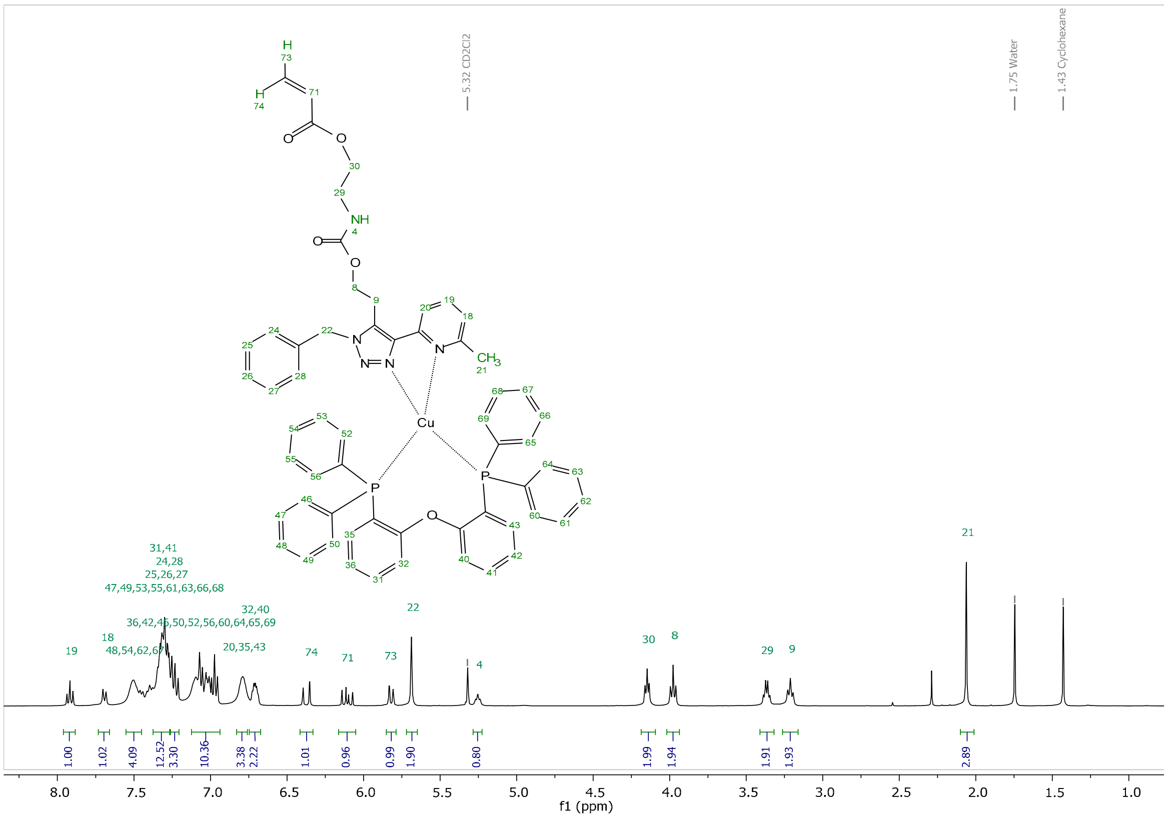

Figure S11. ^{1}H NMR spectrum of **5d** in CD_2Cl_2 (400 MHz).

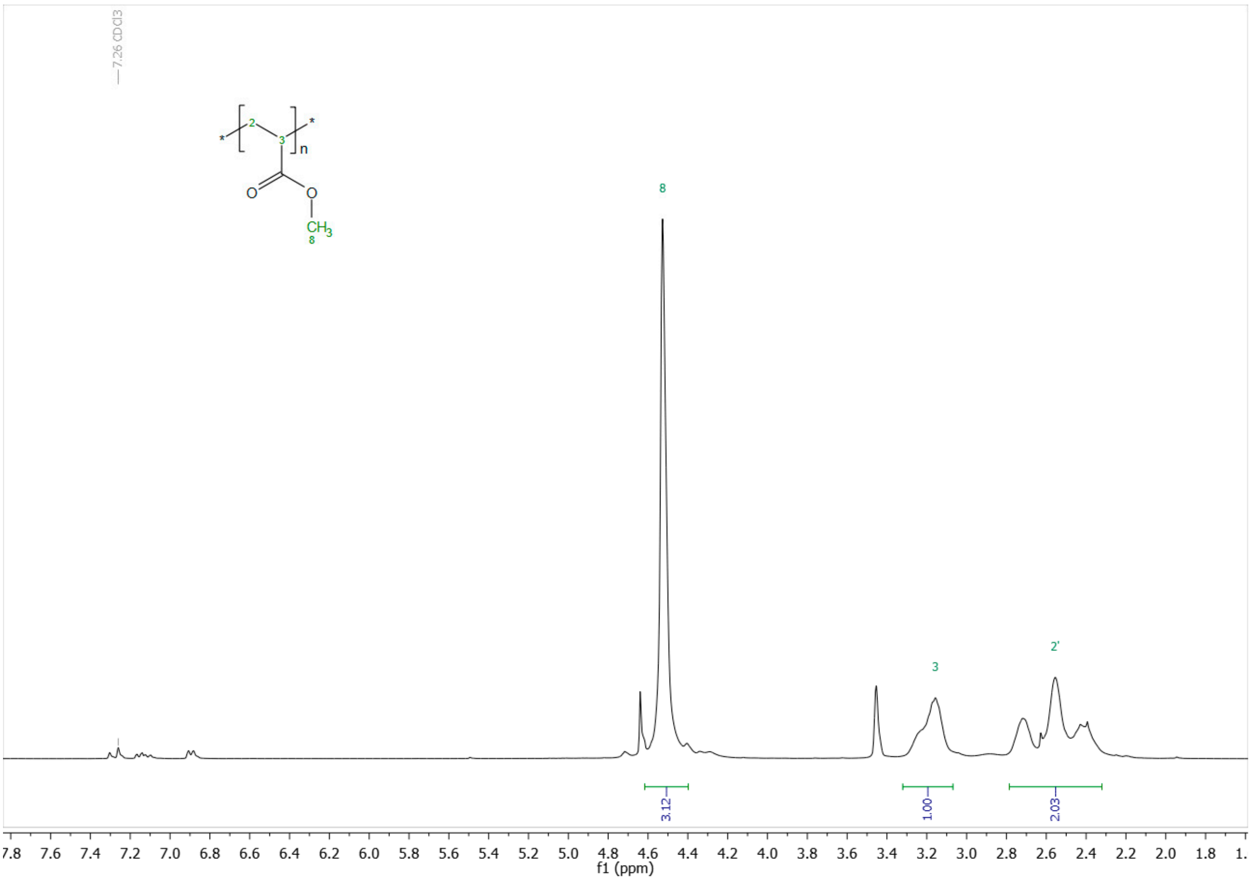

Figure S12. ^{1}H NMR spectrum of **PMA** in $CDCl_3$ (400 MHz).

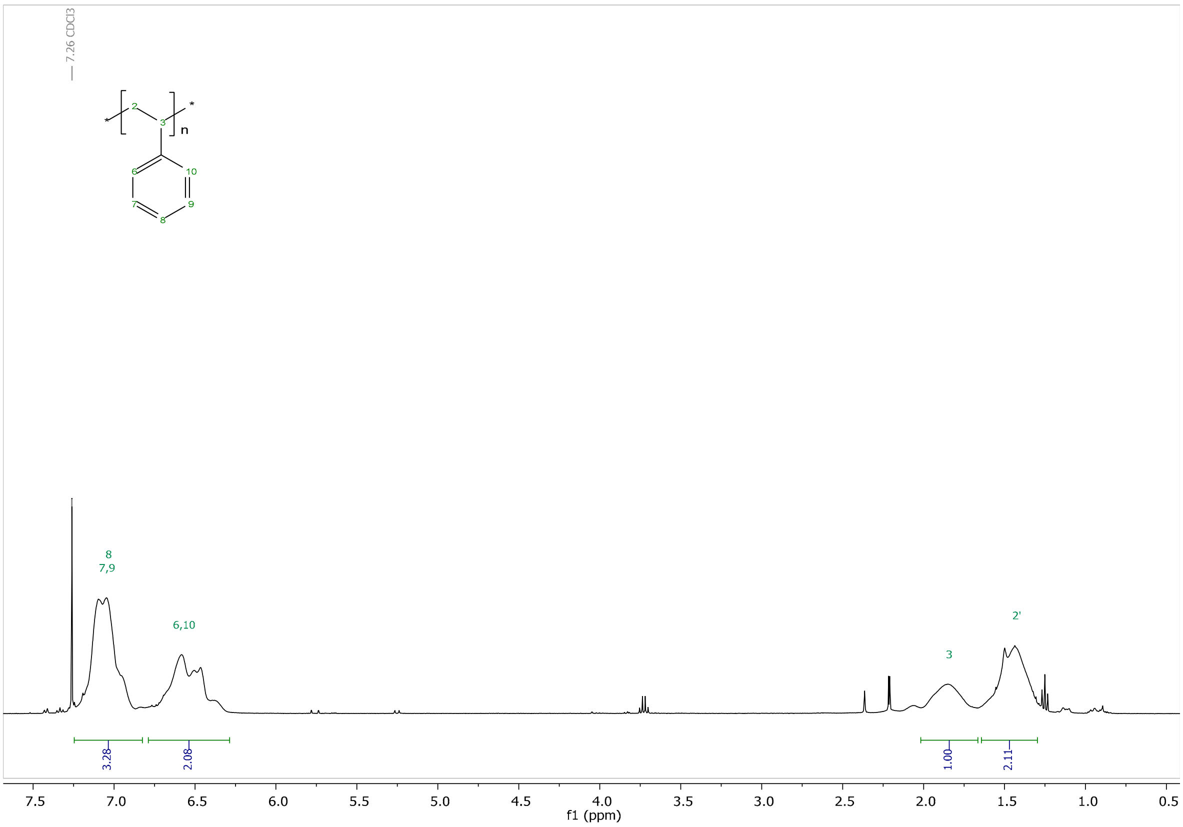

Figure S13. ^{1}H NMR spectrum of **PS** in $CDCl_3$ (400 MHz).

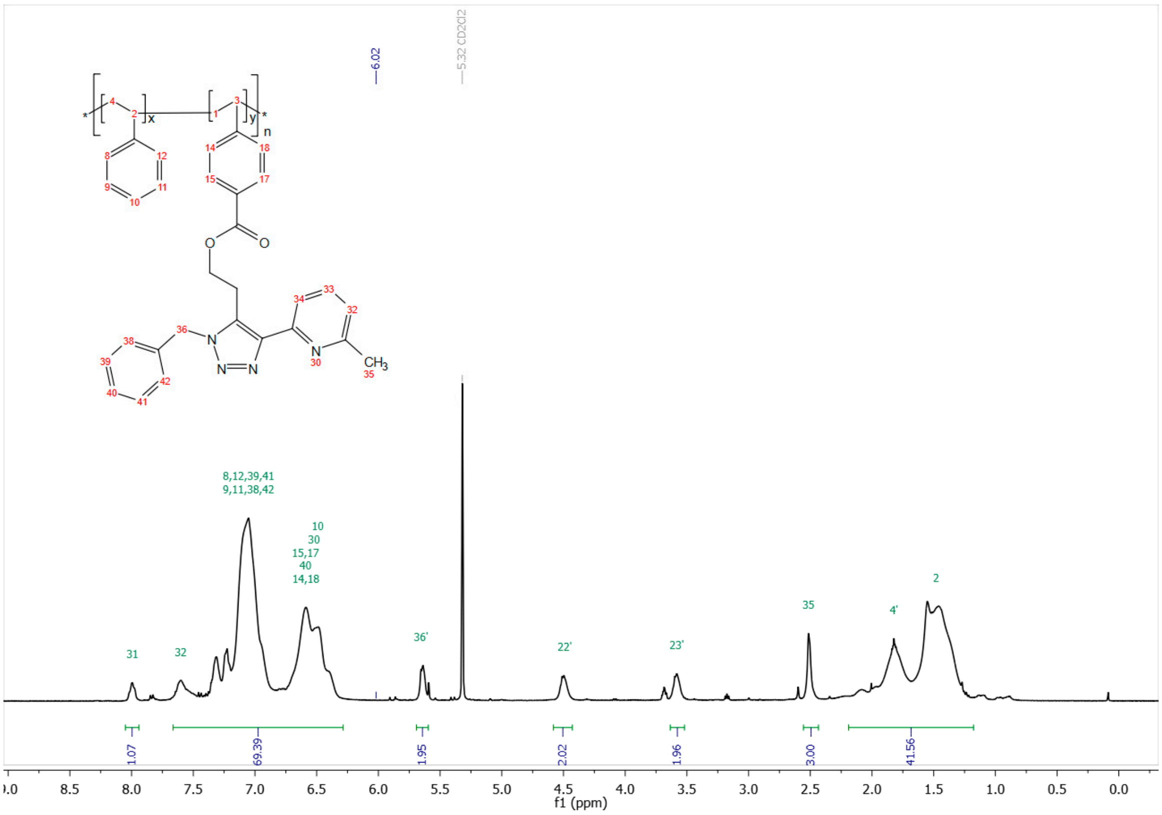

Figure S14. ^{1}H NMR spectrum of **6c** in CD_2Cl_2 (400 MHz).

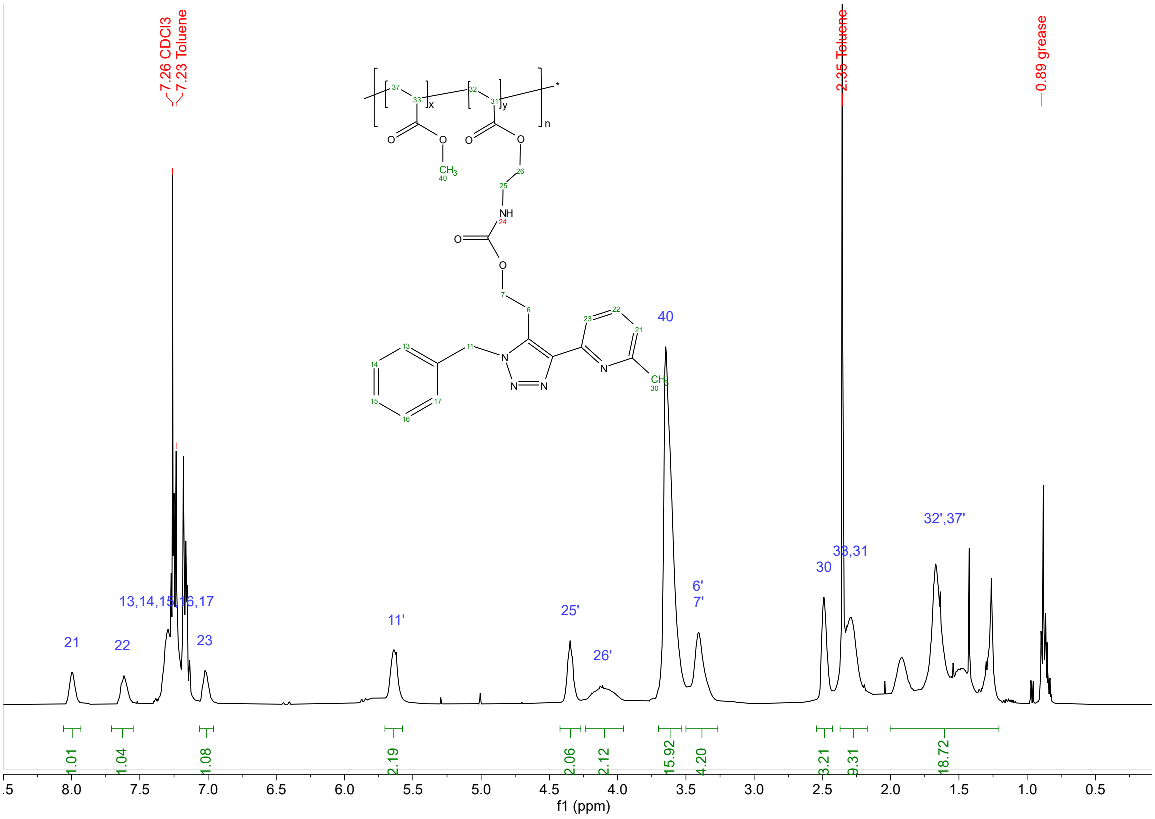

Figure S15. ^{1}H NMR spectrum of **6d** in $CDCl_3$ (400 MHz).

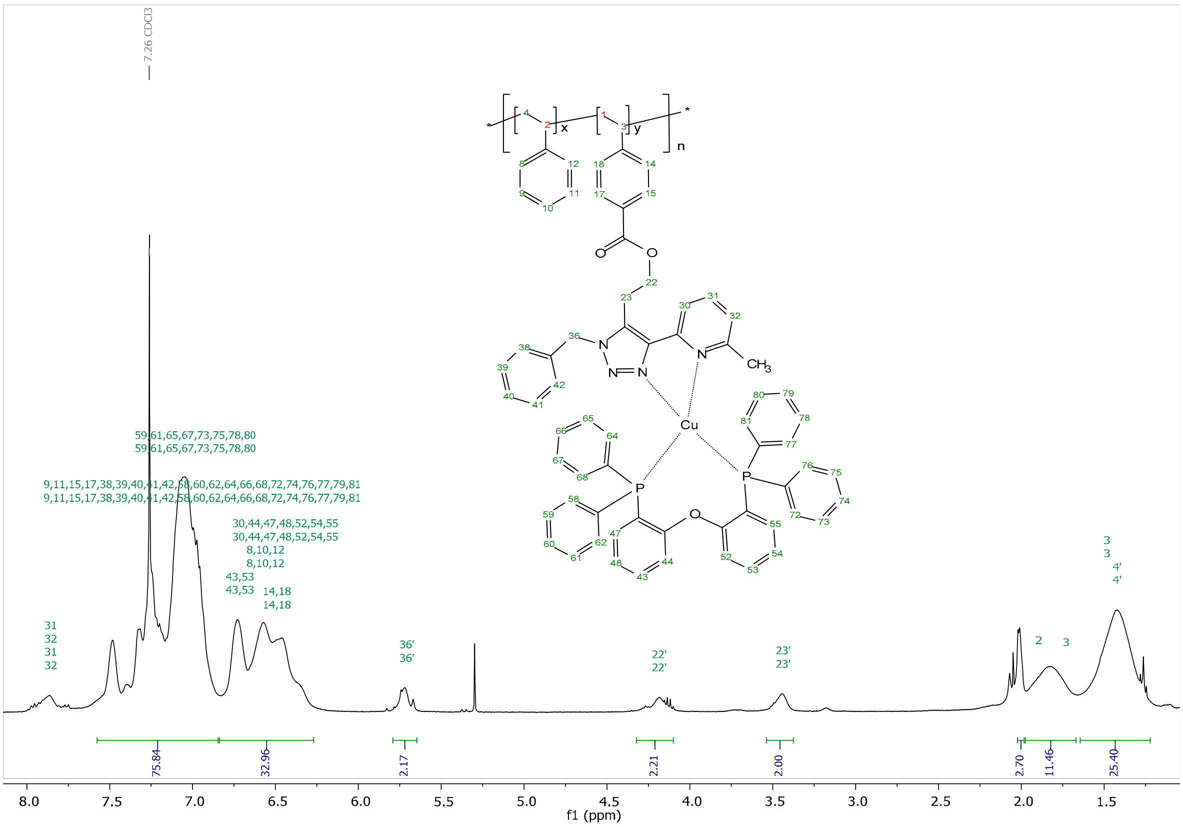

Figure S16. ^{1}H NMR spectrum of **7c** in $CDCl_3$ (400 MHz).

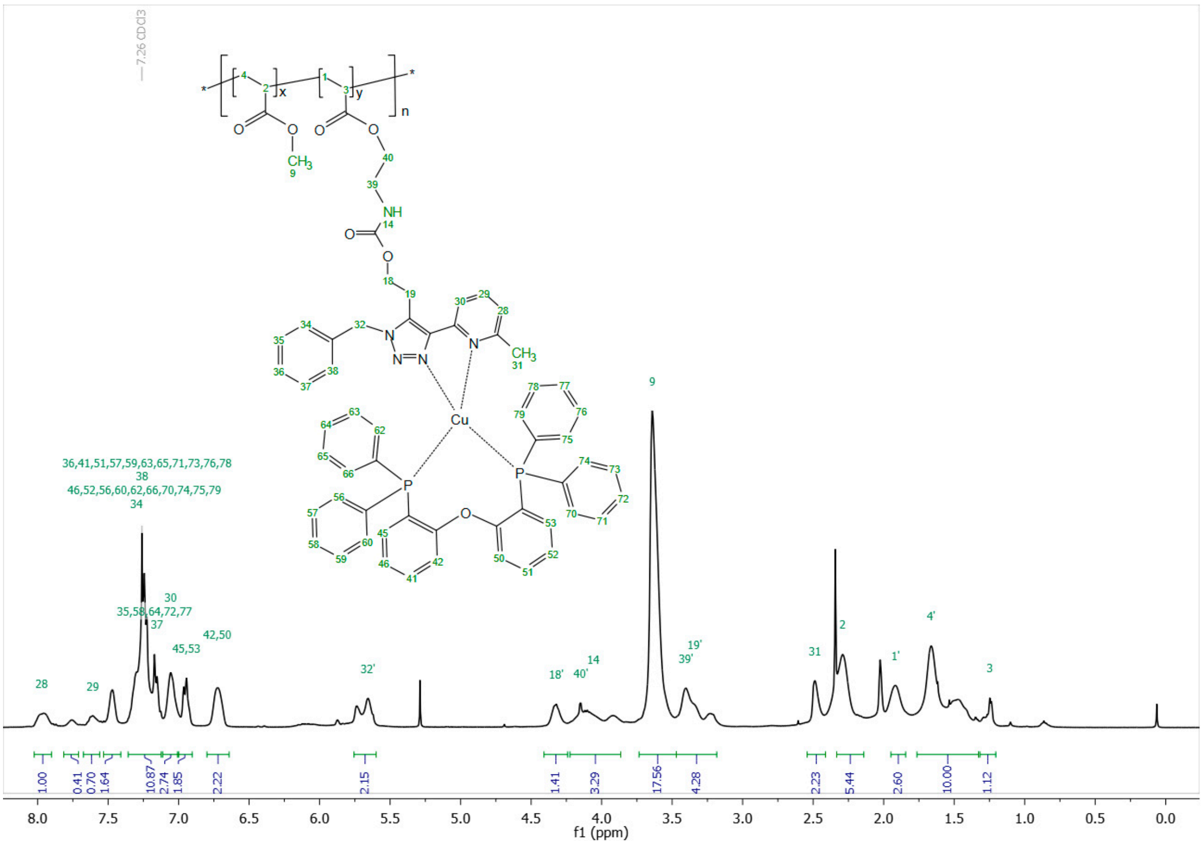

Figure S17. ^{1}H NMR spectrum of **7d** in $CDCl_3$ (400 MHz).

^{13}C NMR spectra

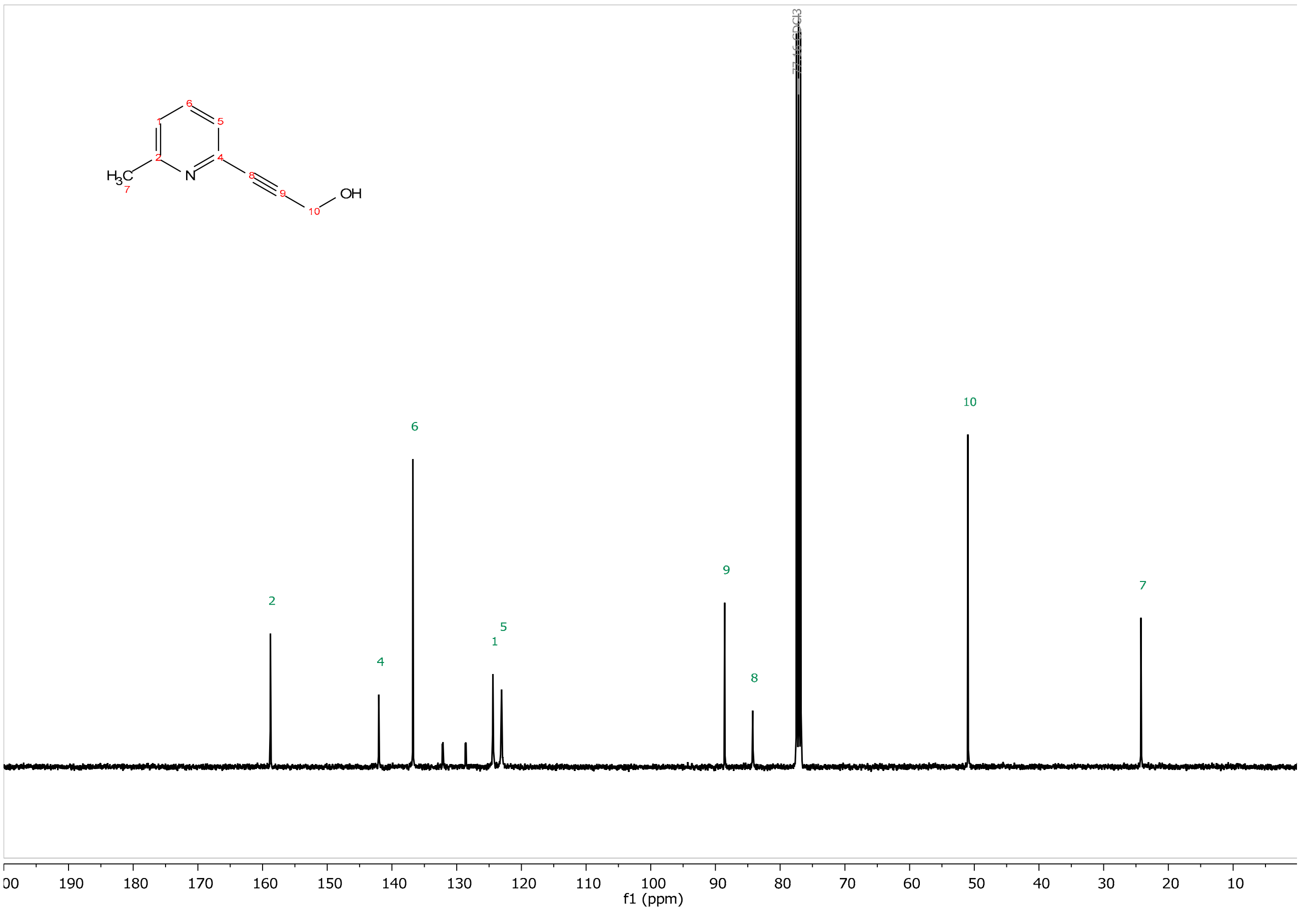

Figure S18. ^{13}C NMR spectrum of **2a** in $CDCl_3$ (101 MHz).

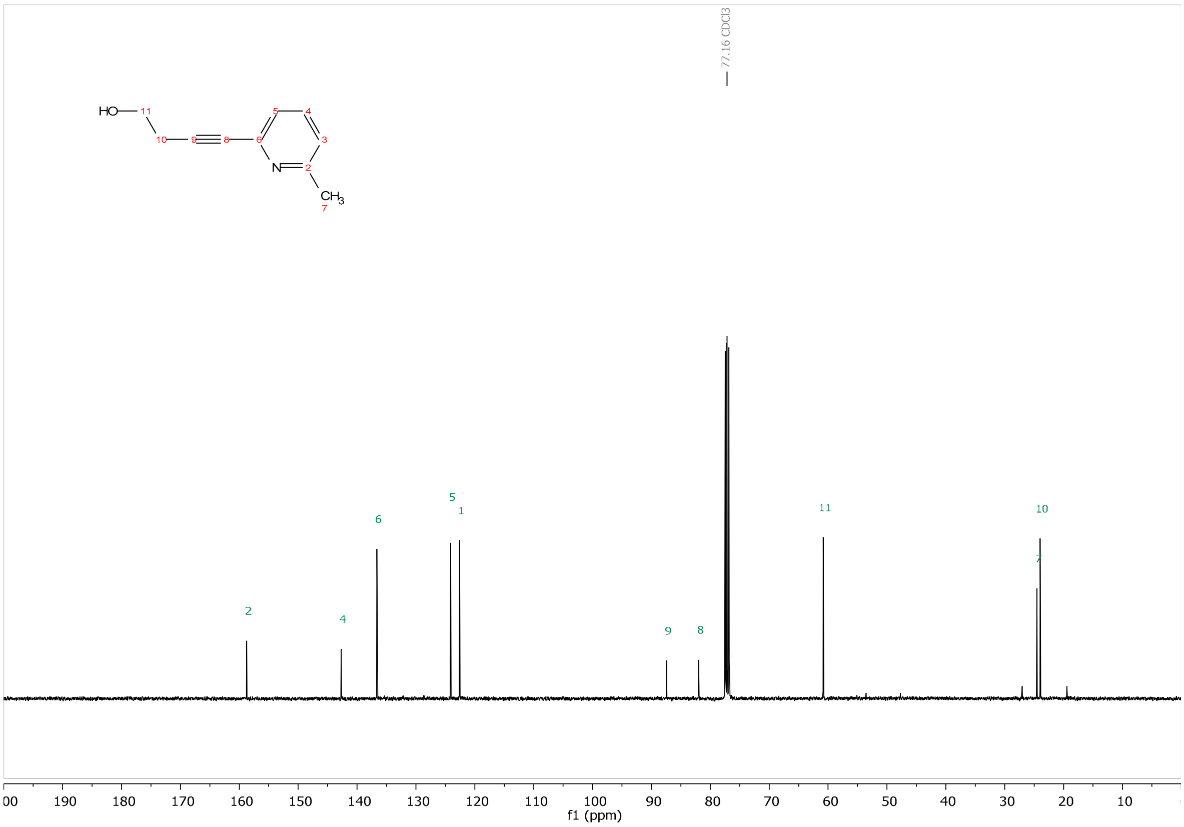

Figure S19. ^{13}C NMR spectrum of **2b** in $CDCl_3$ (101 MHz).

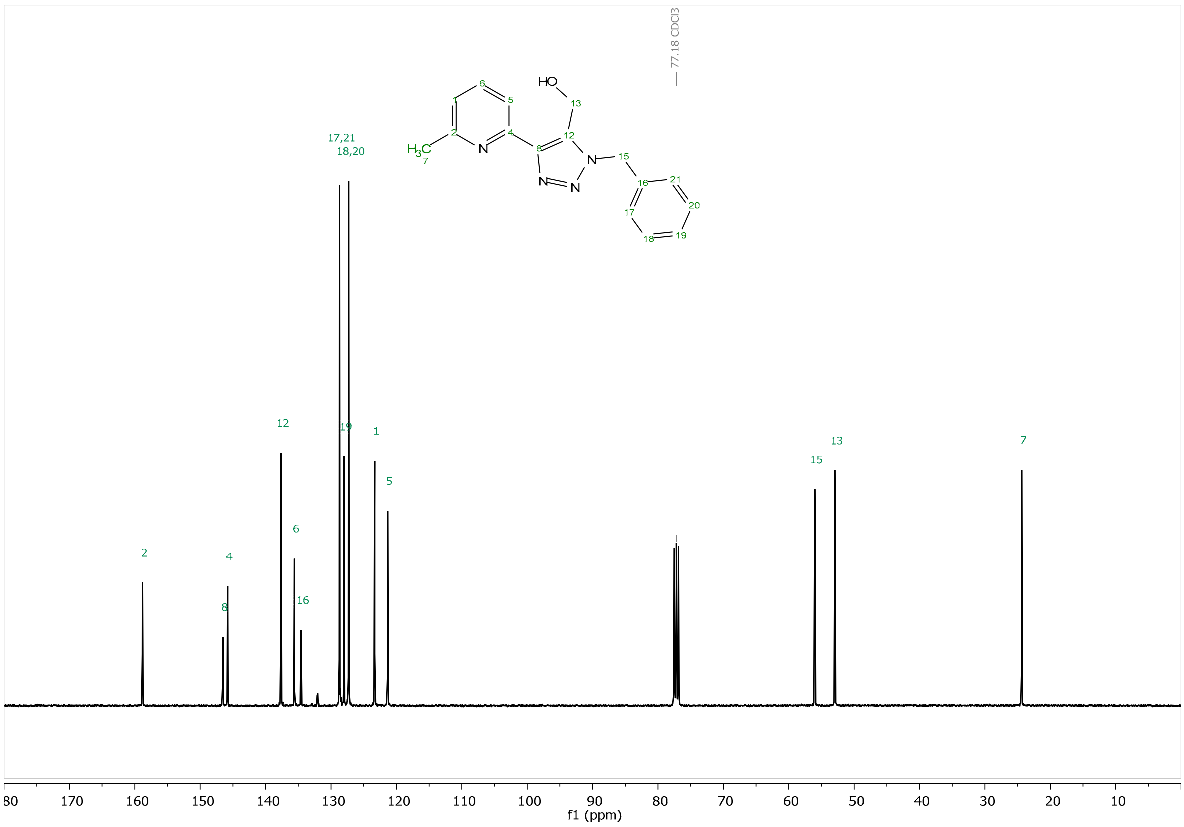

Figure S20. ^{13}C NMR spectrum of **3a** in $CDCl_3$ (101 MHz).

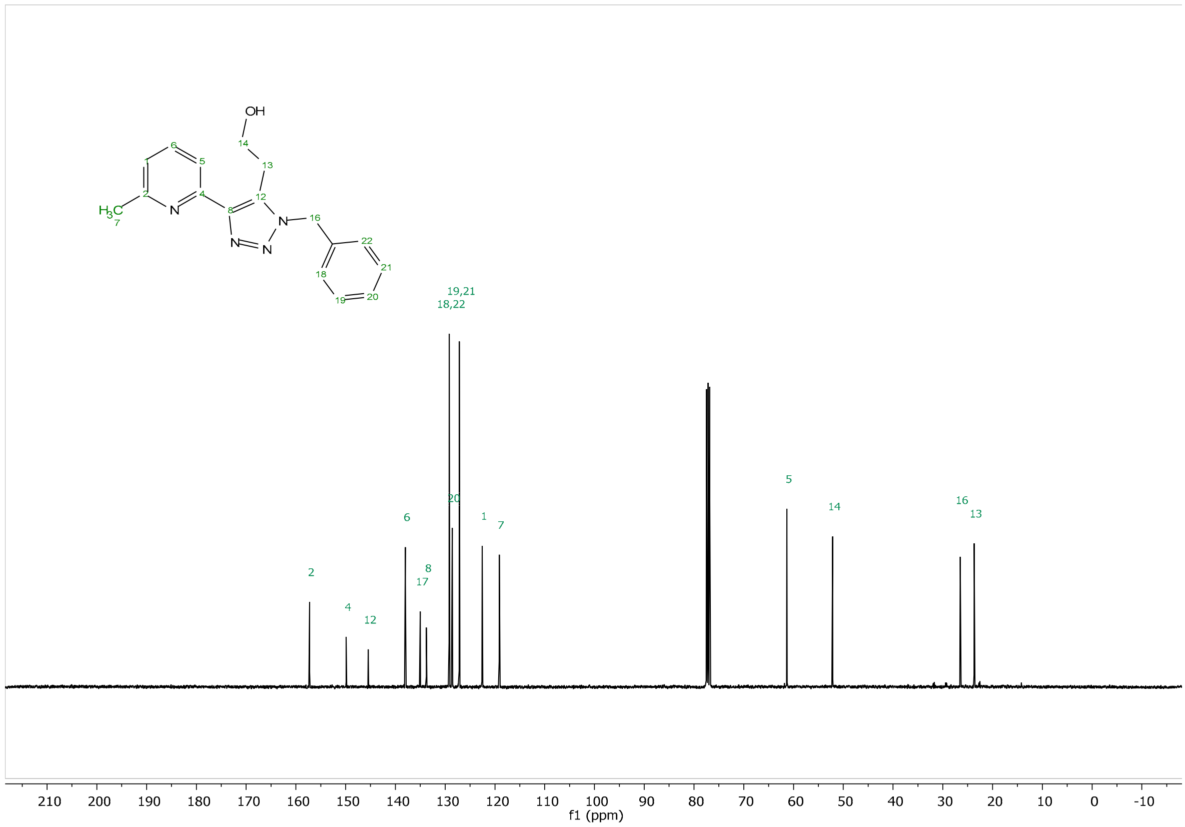

Figure S21. ^{13}C NMR spectrum of **3b** in $CDCl_3$ (101 MHz).

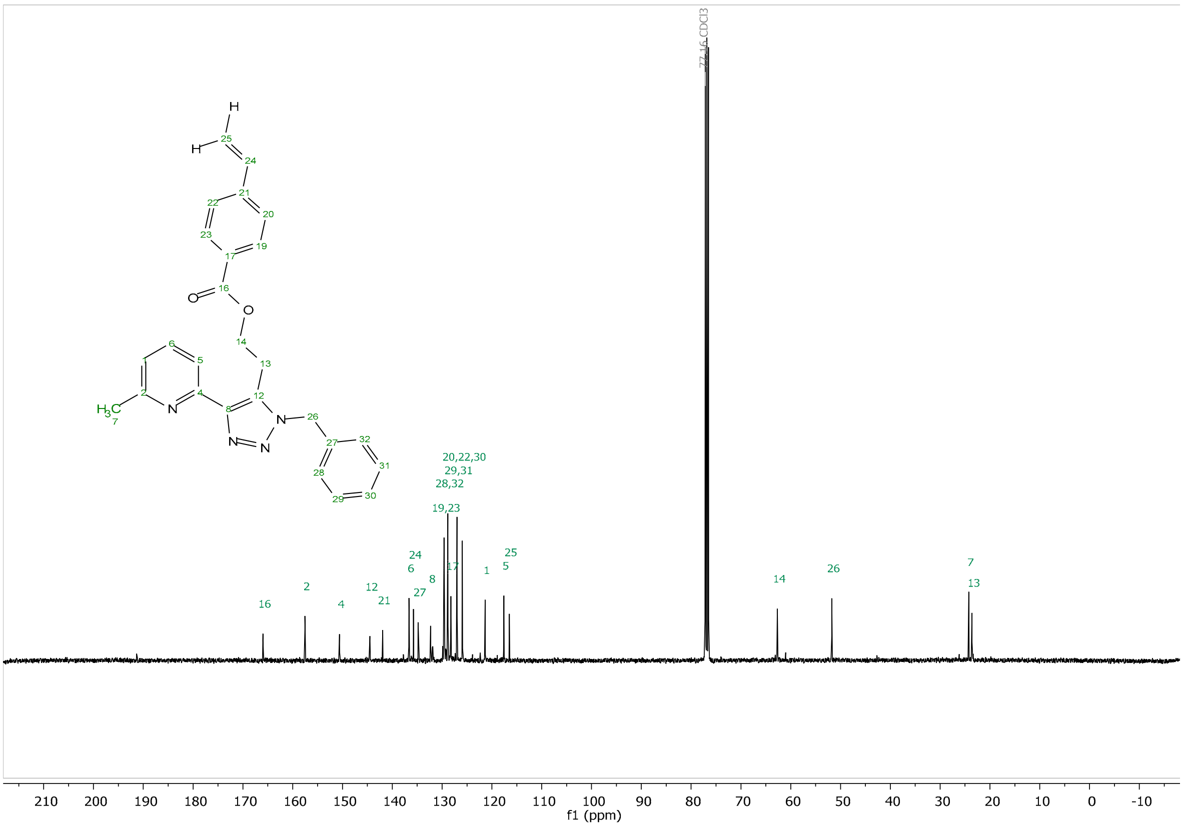

Figure S22. ^{13}C NMR spectrum of **4c** in $CDCl_3$ (101 MHz).

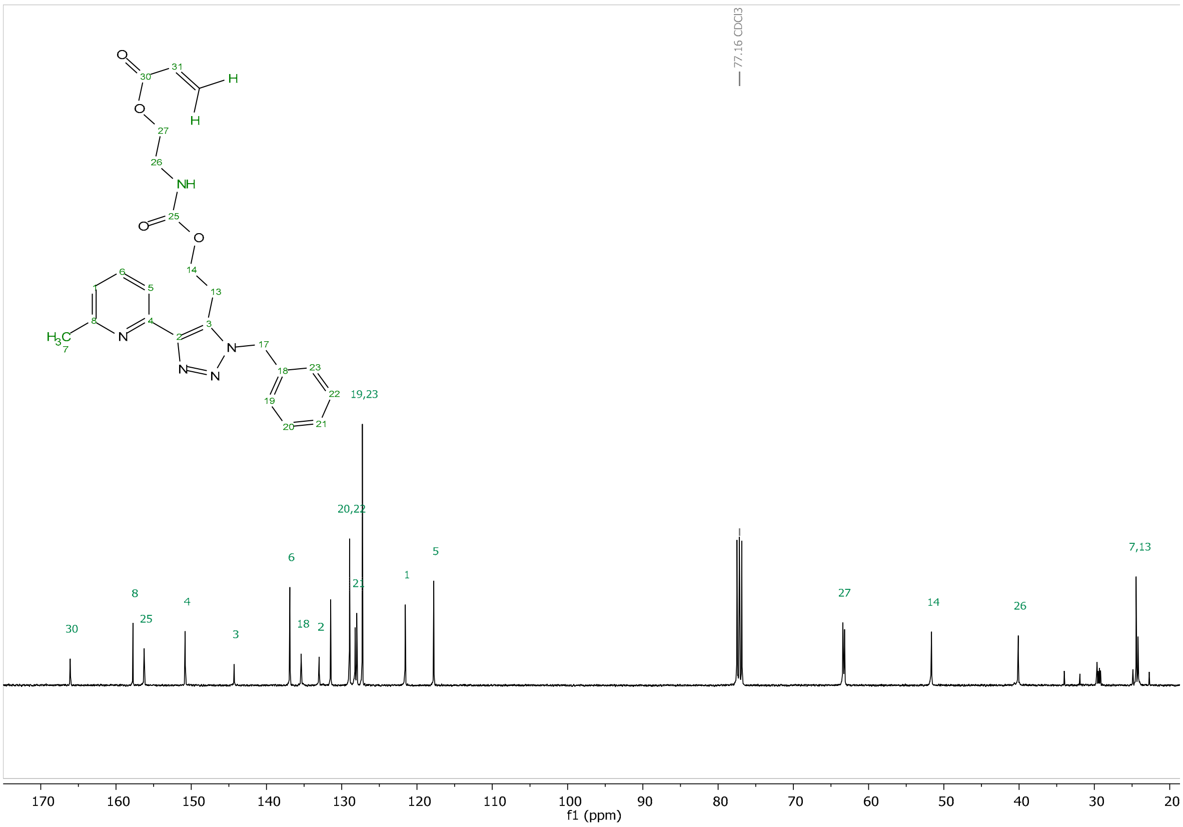

Figure S23. ^{13}C NMR spectrum of **4d** in $CDCl_3$ (101 MHz).

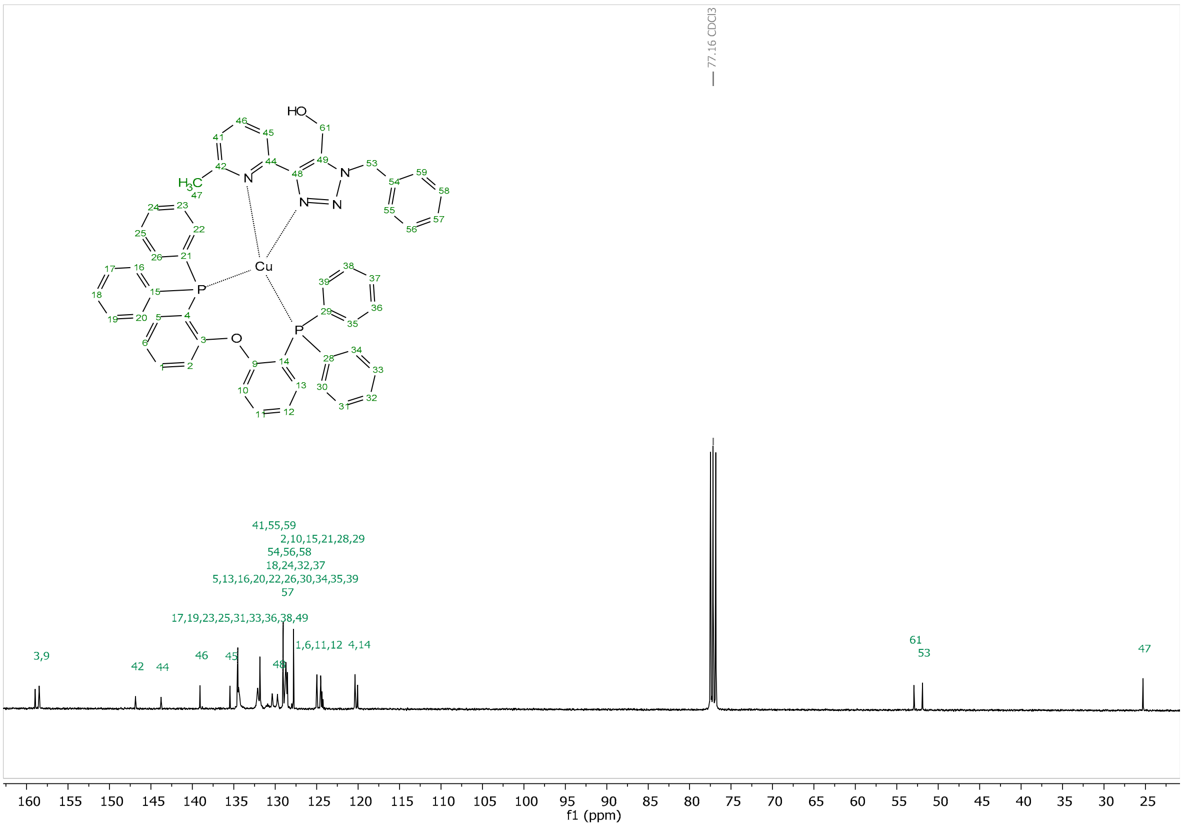

Figure S24. ^{13}C NMR spectrum of **5a** in $CDCl_3$ (101 MHz).

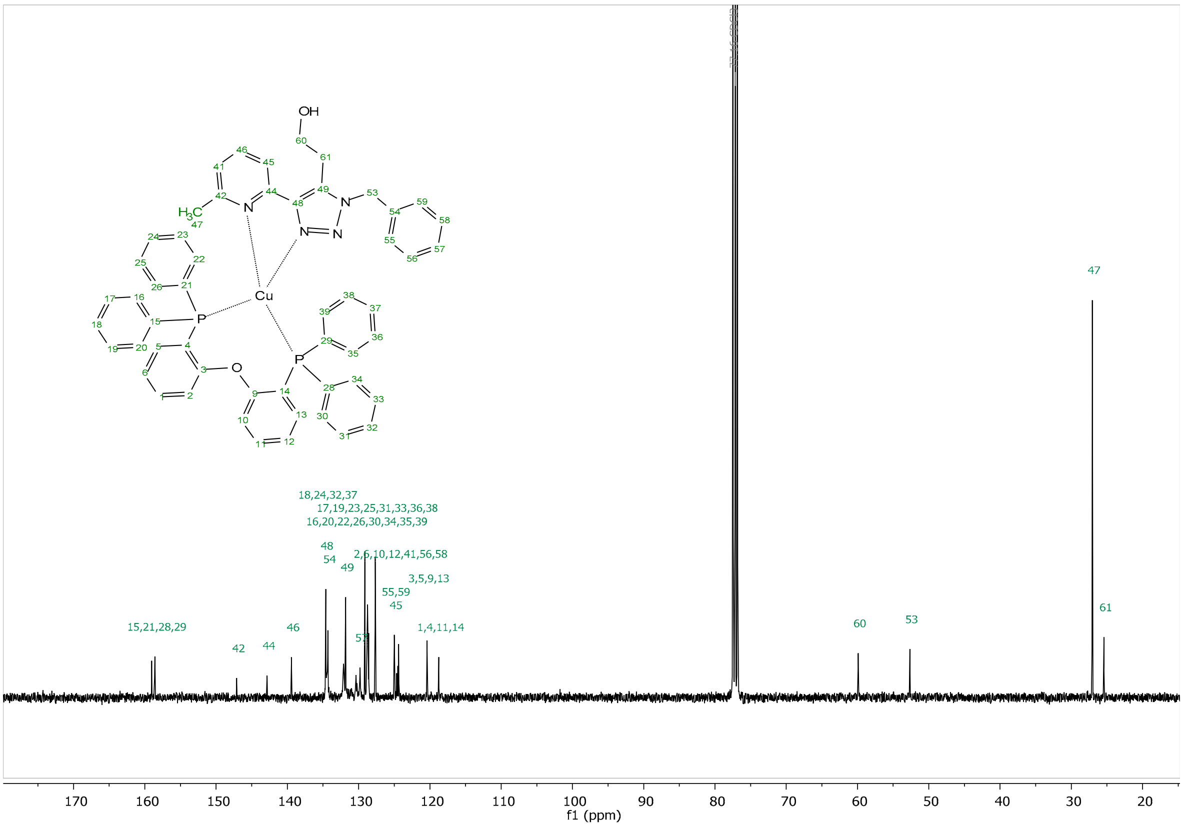

Figure S25. ^{13}C NMR spectrum of **5b** in $CDCl_3$ (101 MHz).

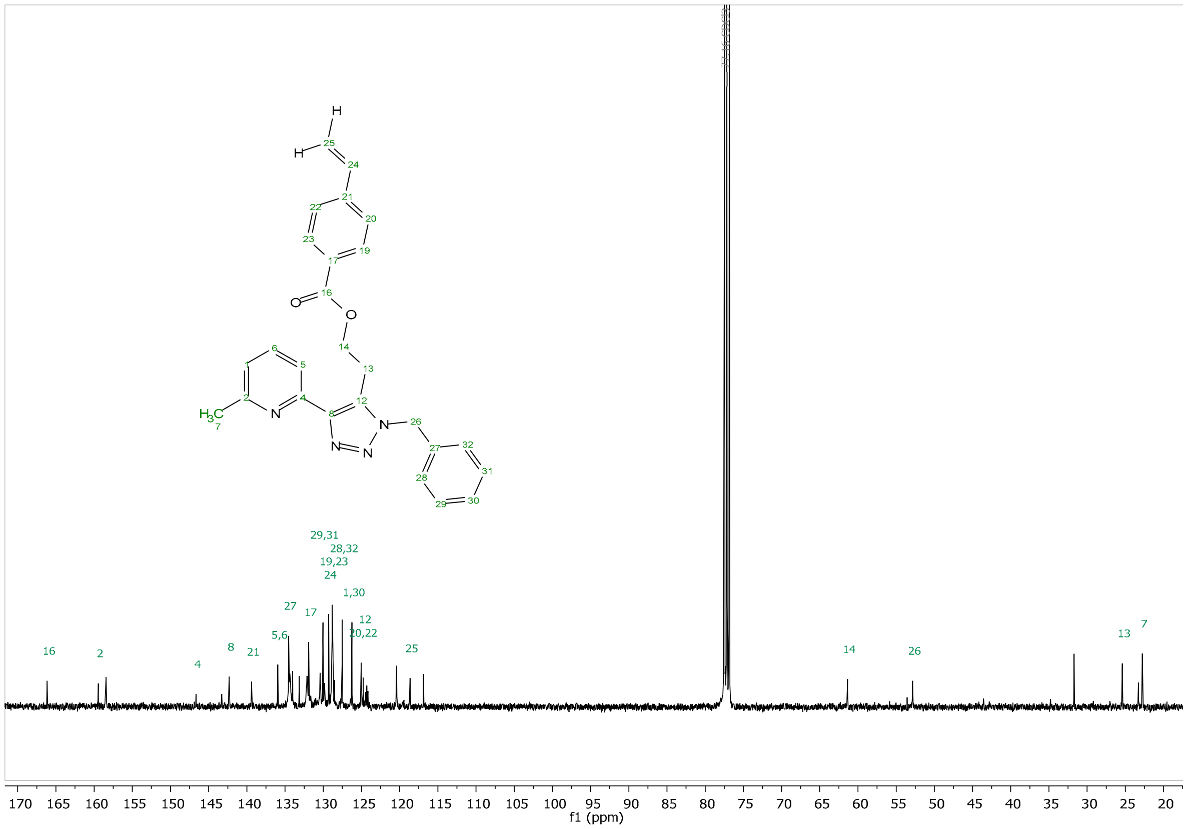

Figure S26. ^{13}C NMR spectrum of **5c** in CD_2Cl_2 (101 MHz).

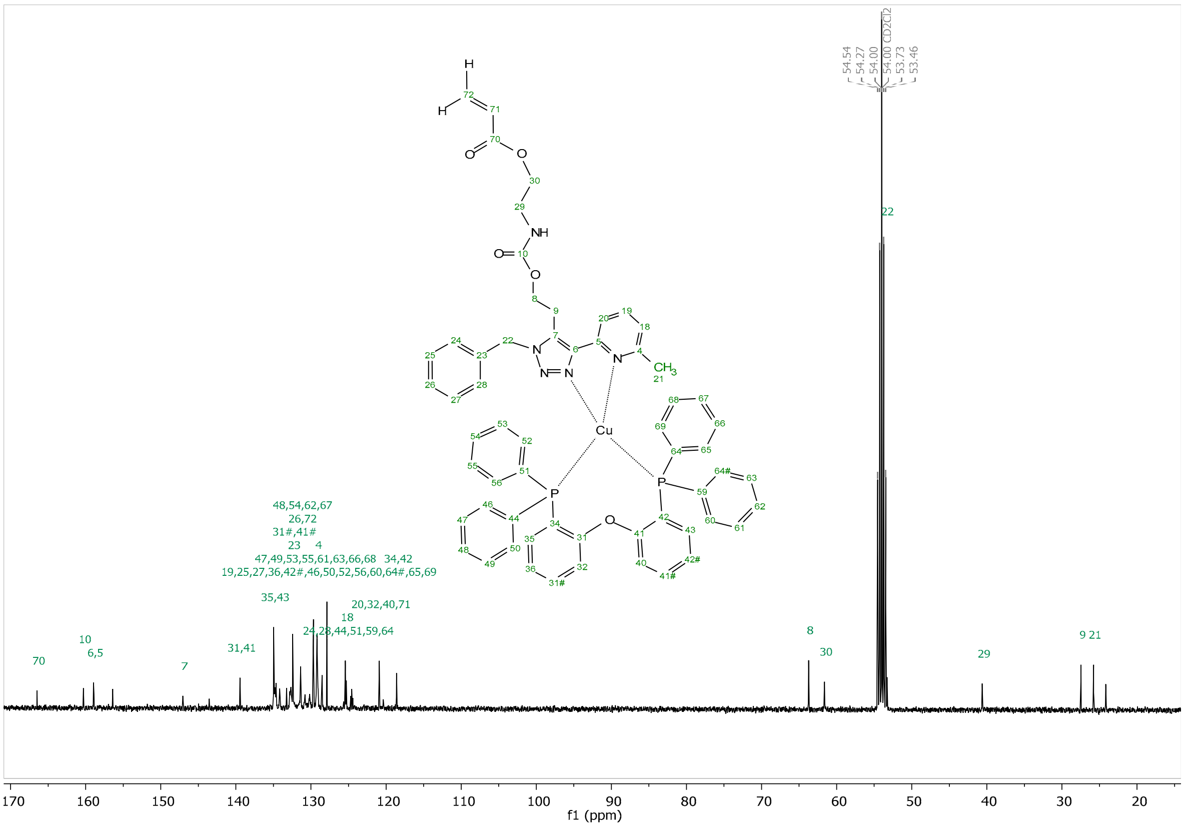

Figure S27. ^{13}C NMR spectrum of **5d** in CD_2Cl_2 (101 MHz).

FTIR spectra

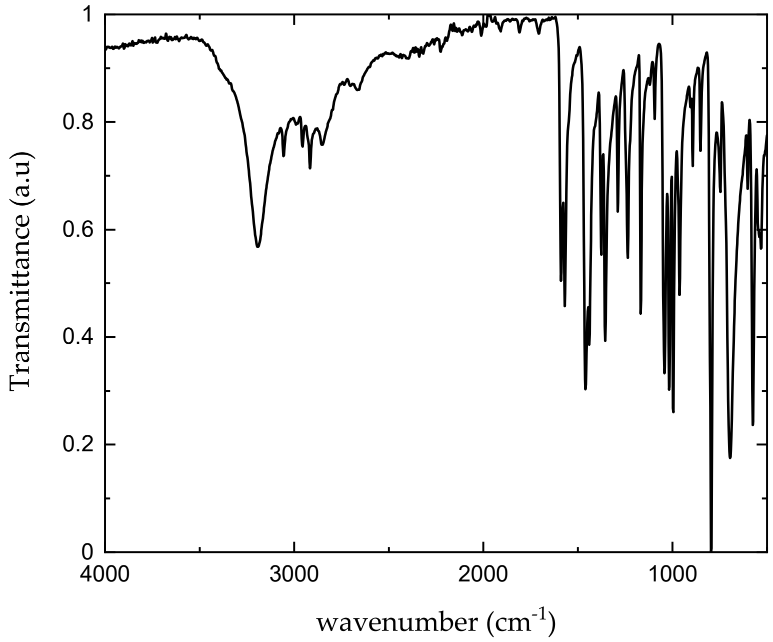

Figure S28. FTIR spectrum of **2a**.

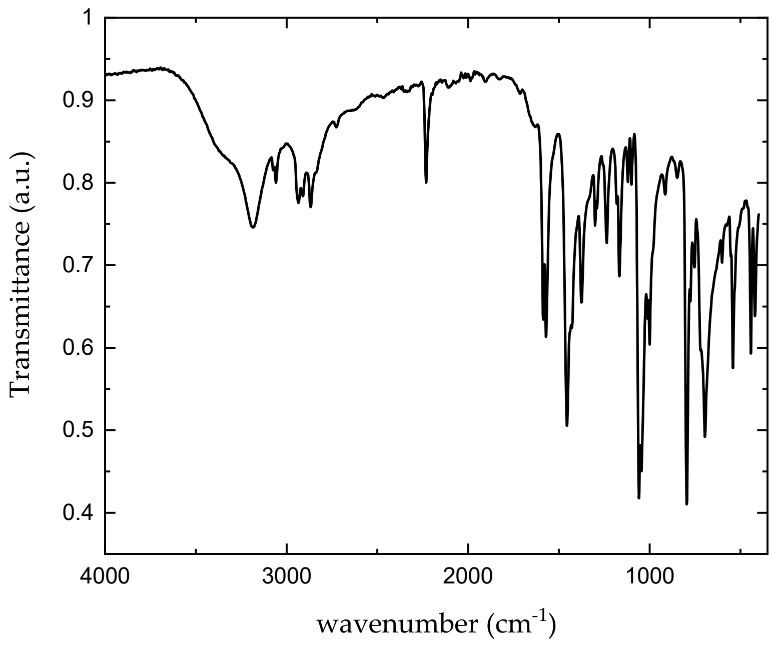

Figure S29. FTIR spectrum of **2b**.

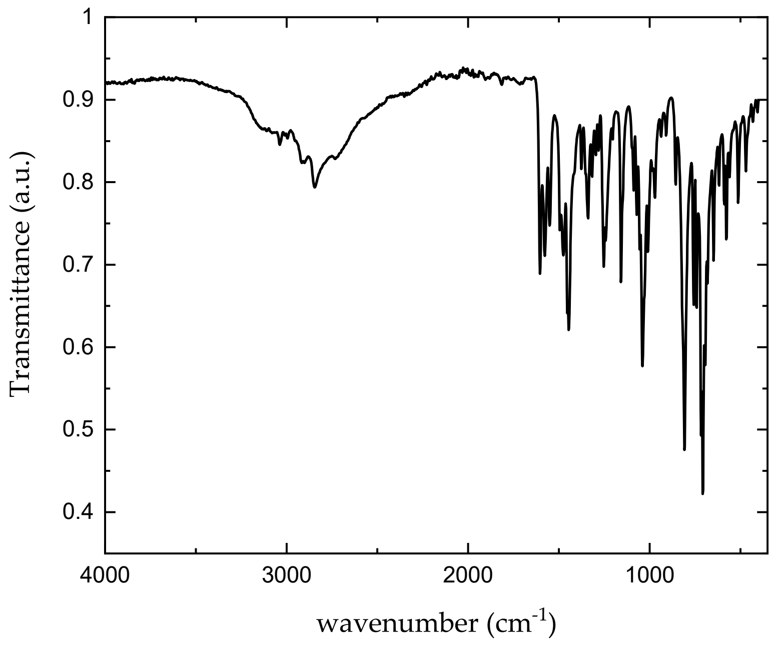

Figure S30. FTIR spectrum of **3a**.

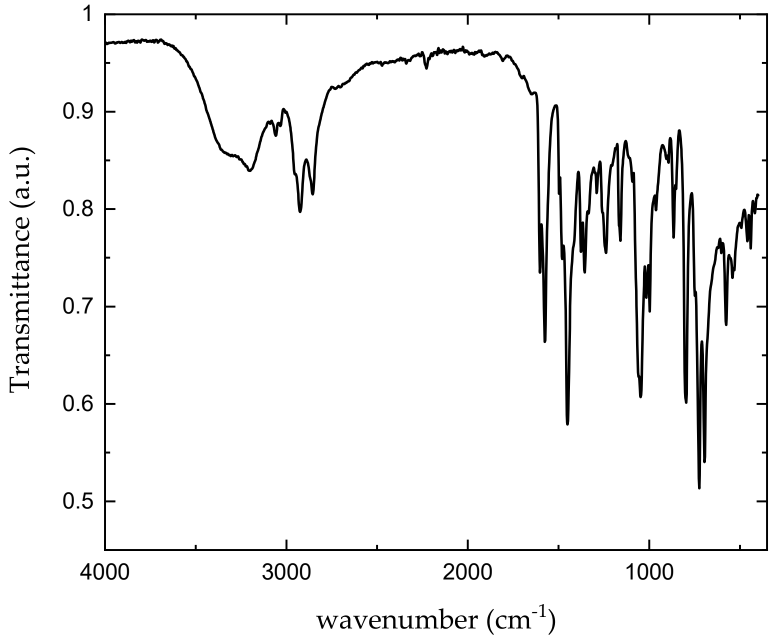

Figure S31. FTIR spectrum of **3b**.

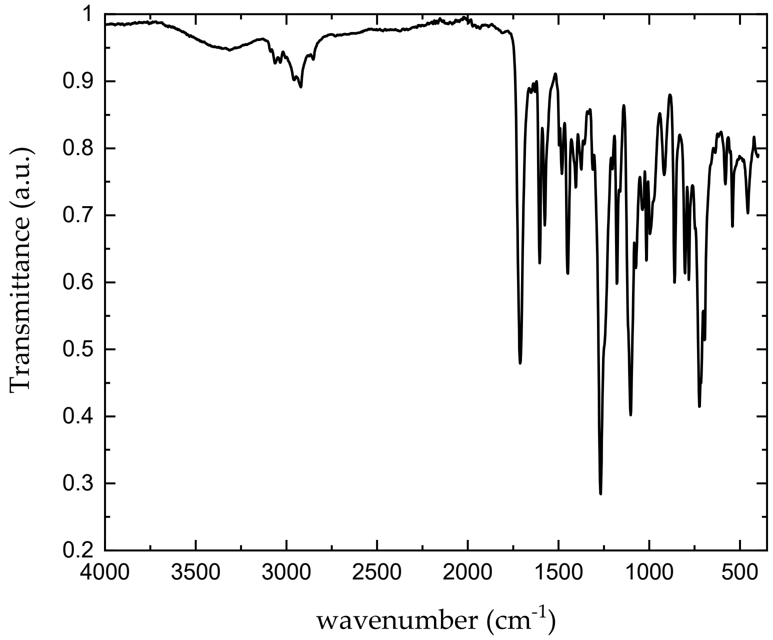

Figure S32. FTIR spectrum of **4c**.

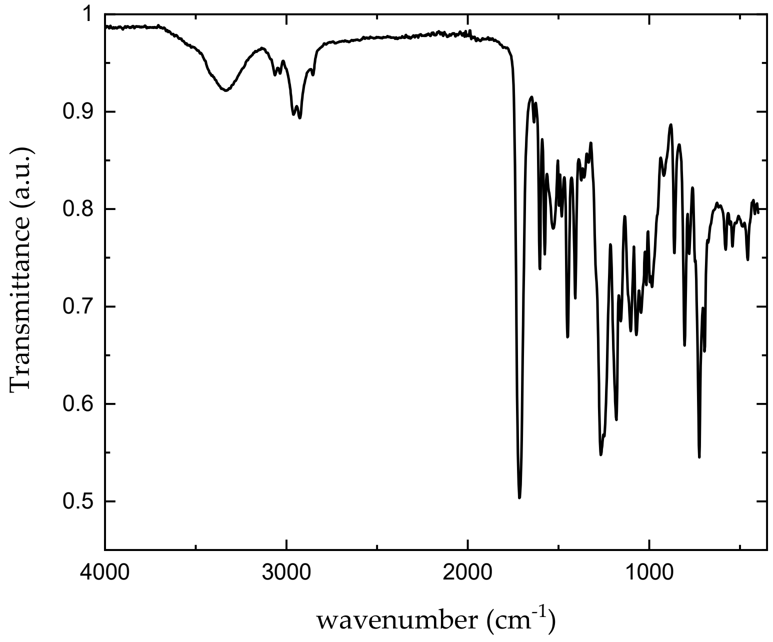

Figure S33. FTIR spectrum of **4d**.

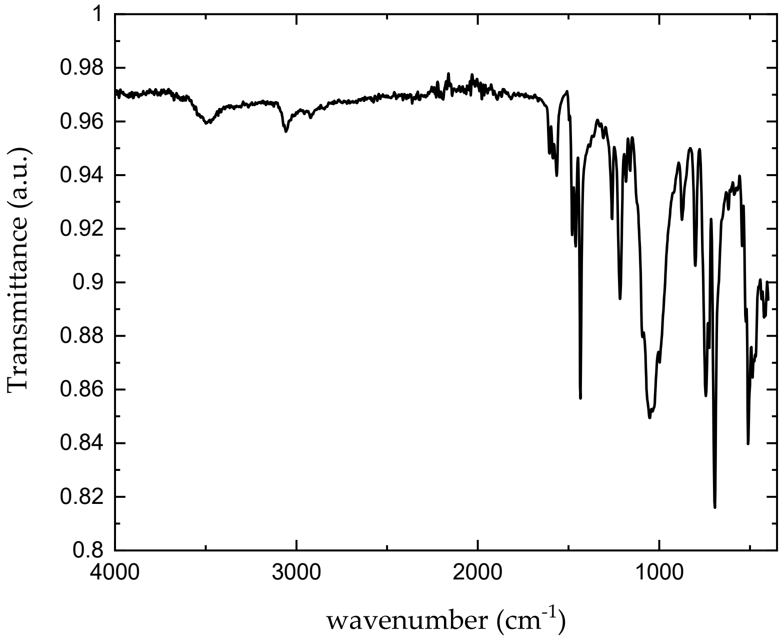

Figure S34. FTIR spectrum of **5a**.

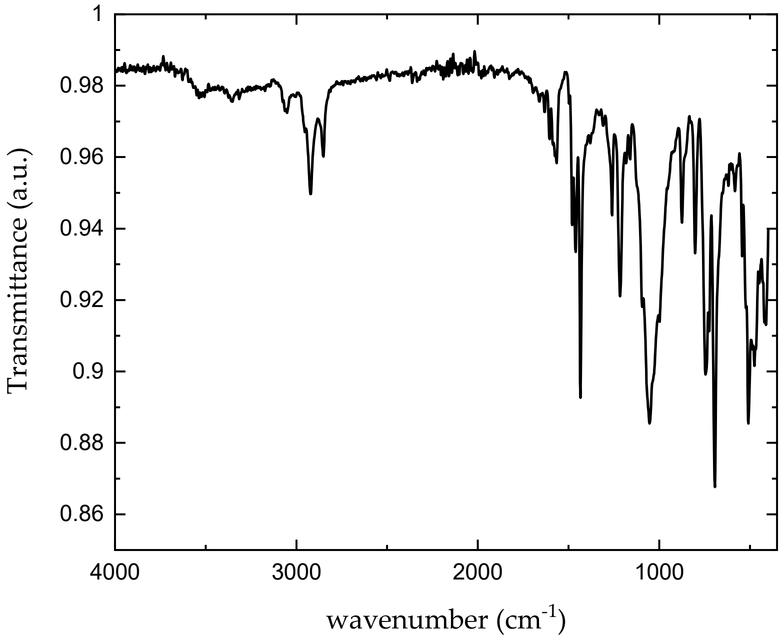

Figure S35. FTIR spectrum of **5b**.

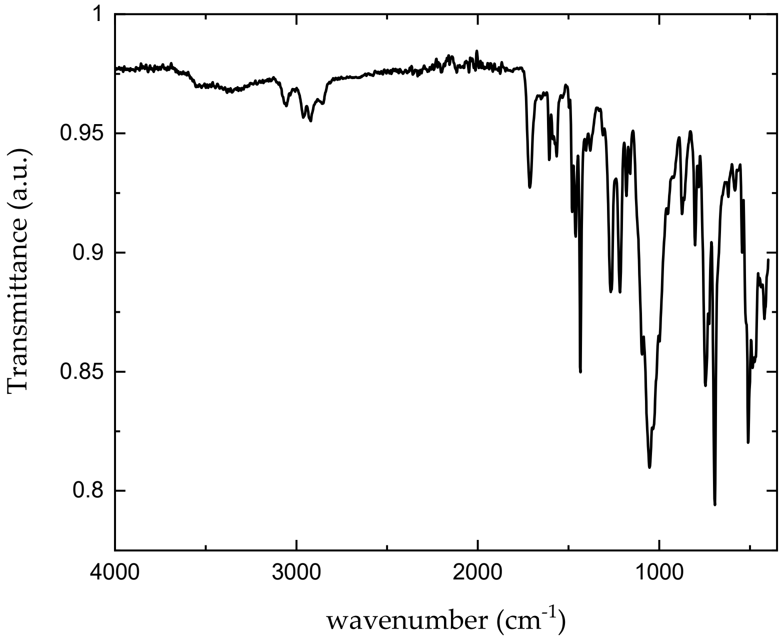

Figure S36. FTIR spectrum of **5c**.

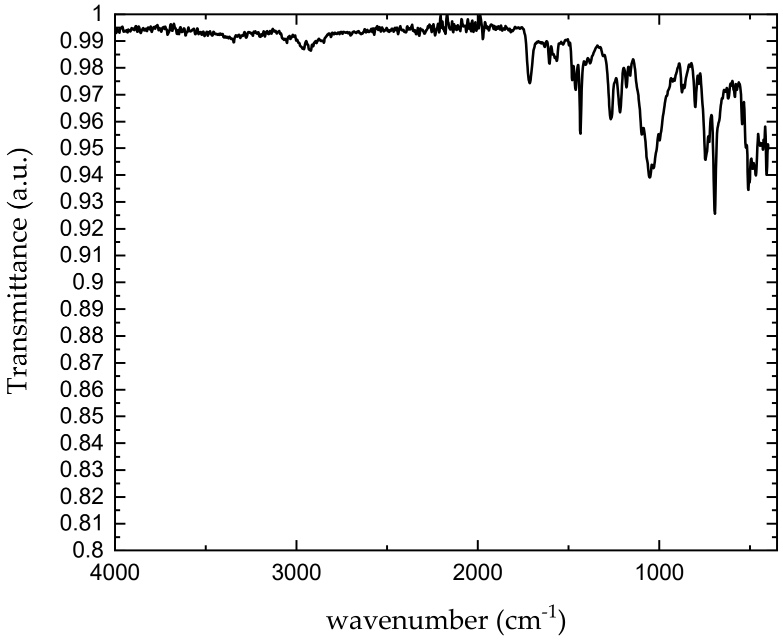

Figure S37. FTIR spectrum of **5d**.

SEC Chromatograms

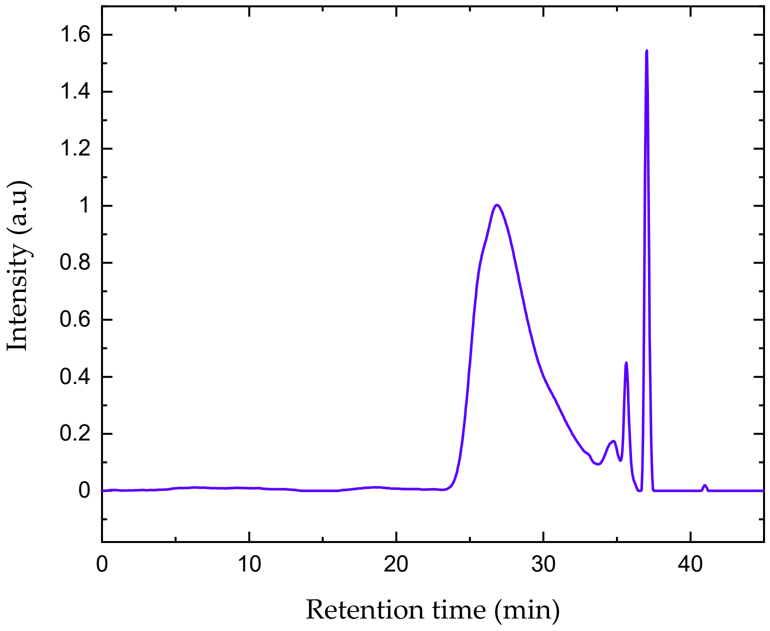

Figure S38. SEC of **PMA** in THF.

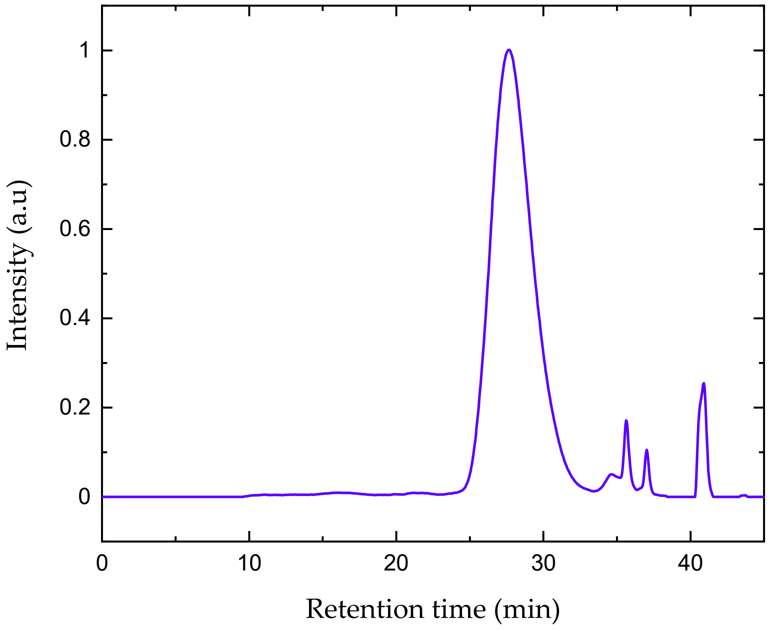

Figure S39. SEC of **PS** in THF.

Photophysical data

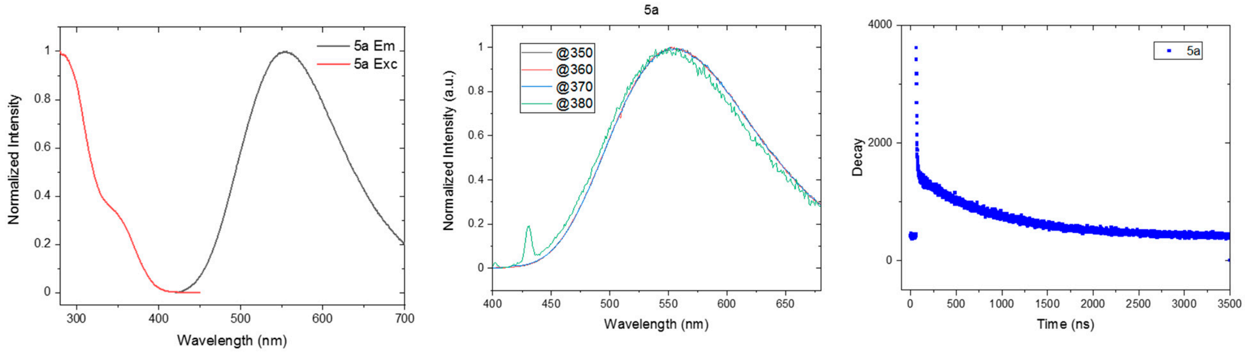

Figure S40. Left: Emission (black curve) and Excitation (red curve) spectra of the compound **5a** in DCM, Ar-saturated solution. Excitation wavelength= 370 nm. Emission maximum: 552 nm. Centre: Emission spectra of **5a** at different excitation wavelength. Right: Photoluminescence lifetime decay of **5a**, with TCSPC excitation NanoLED370 and measurement range 3.2μs

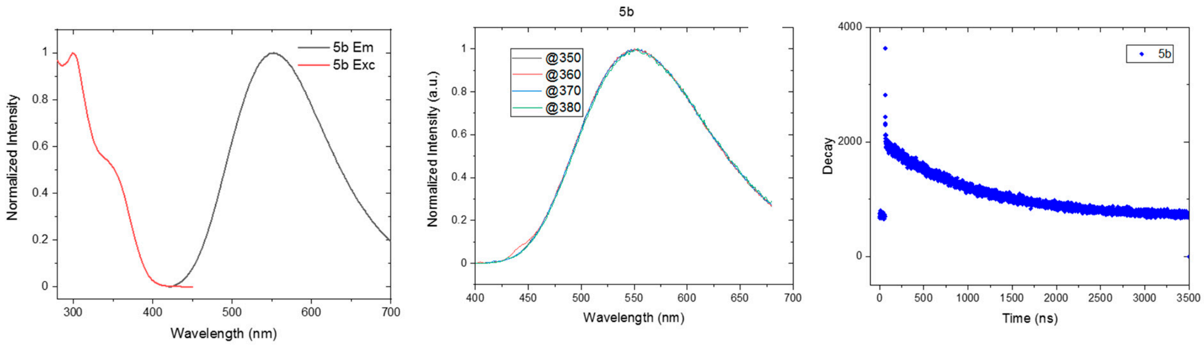

Figure S41.Left: Emission (black curve) and Excitation (red curve) spectra of the compound **5b** in DCM, Ar-saturated solution. Excitation wavelength= 370 nm. Emission maximum: 552 nm. . Centre: Emission spectra of **5b** at different excitation wavelength. Right: Photoluminescence lifetime decay of 5b, with TCSPC excitation NanoLED370 and measurement range 3.2μs

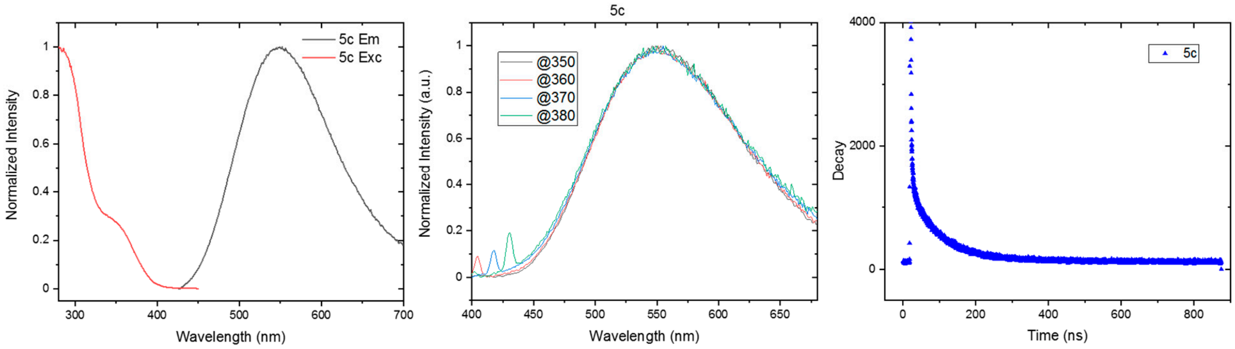

Figure S42. Left: Emission (black curve) and Excitation (red curve) spectra of the compound **5c** in DCM, Ar-saturated solution. Excitation wavelength = 370 nm. Emission maximum: 548 nm. Centre: Emission spectra of **5c** at different excitation wavelength. Right: Photoluminescence lifetime decay of **5c**, with TCSPC excitation NanoLED370 and measurement range 1.6 μs

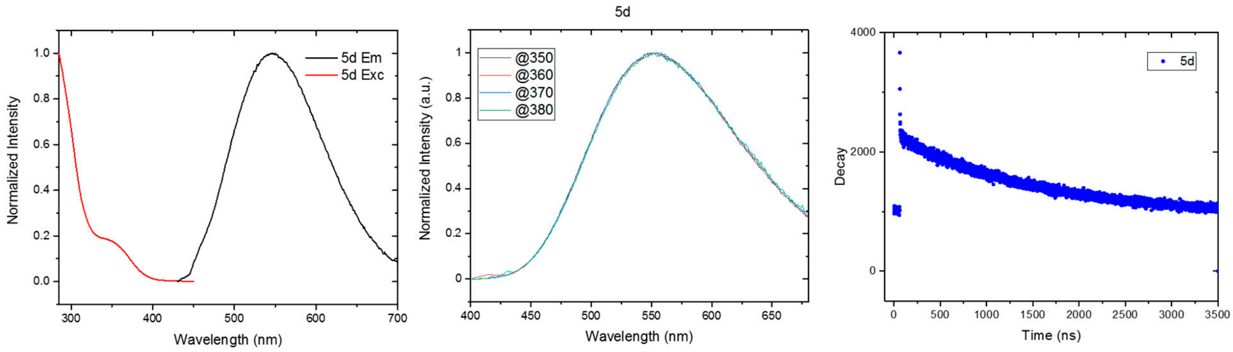

Figure S43.Left: Emission (black curve) and Excitation (red curve) spectra of the compound **5d** in DCM, Ar-saturated solution. Excitation wavelength = 370 nm. Emission maximum: 550 nm. Centre: Emission spectra of **5d** at different excitation wavelength. Right: Photoluminescence lifetime decay of **5d**, with TCSPC excitation NanoLED370 and measurement range 3.2μs

Computational data

Table S1. HOMO and LUMO energies of complexes in vacuum.

Sample	HOMO / eV	LUMO/ eV	ΔHOMO-LUMO /eV
**5b**	-7.82	-3.47	4.35
**5c**	-7.72	-3.60	4.17
**5d**	-7.92	-3.55	4.37

[a] optimised at PBE0/6-31G**/LANL2DZ/Grimme dispersion level (vacuum).

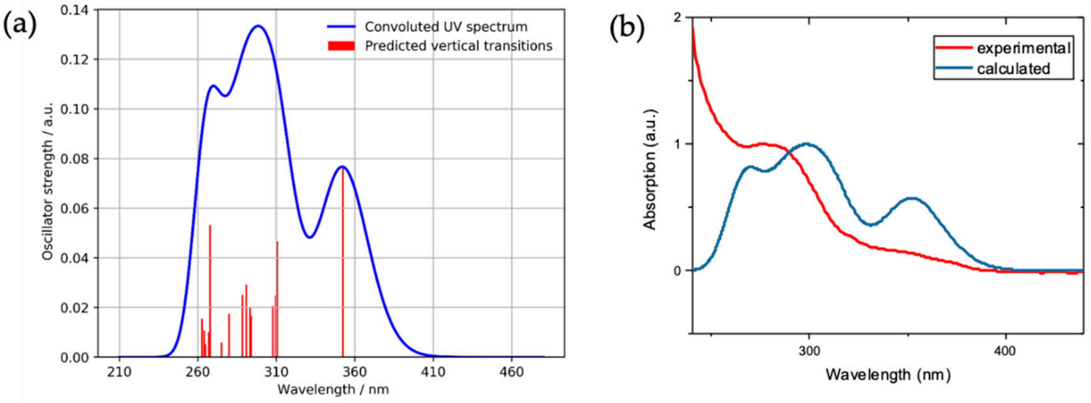

Figure S44. (a) Convoluted UV-vis spectrum of **5b** from predicted vertical transitions. (b) Agreement of the experimental UV-vis absorption spectrum (red curve) of 5b and the theoretical spectrum (blue curve).

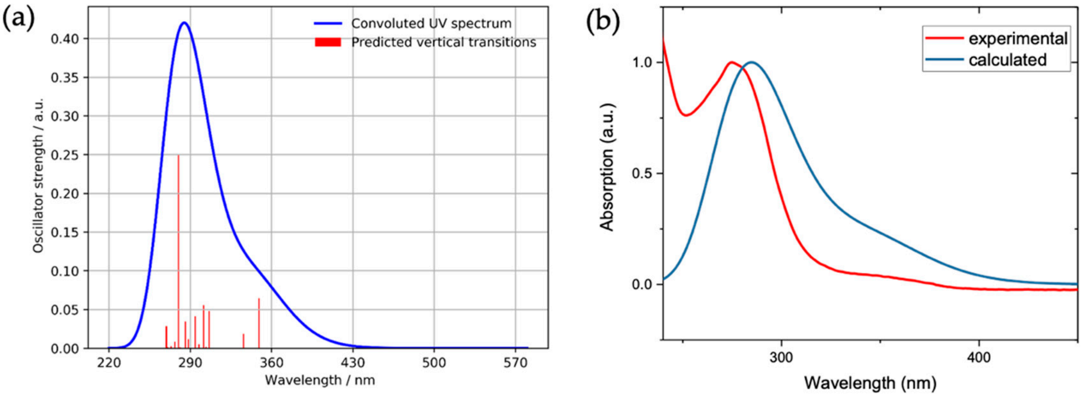

Figure S45. (a) Convoluted UV-vis spectrum of **5c** from predicted vertical transitions. (b) Agreement of the experimental UV-vis absorption spectrum of **5c** (red curve) and the theoretical spectrum (blue curve).

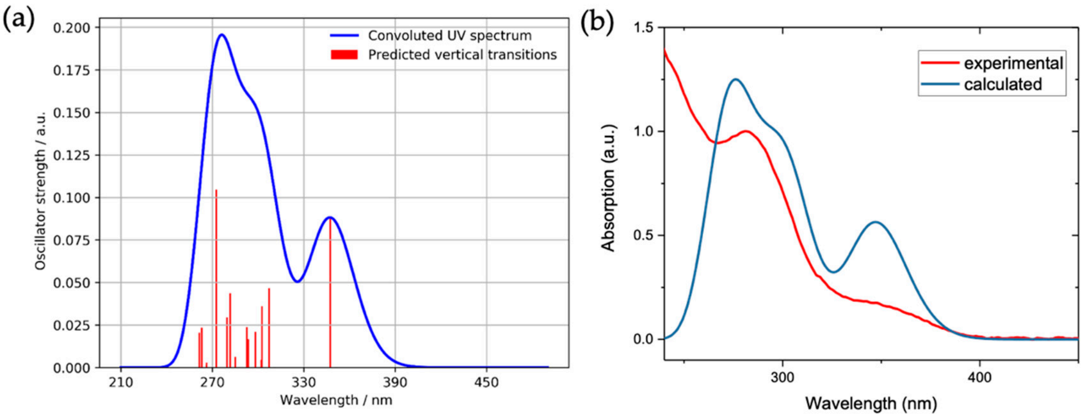

Figure S46. (a) Convoluted UV-vis spectrum of **5d** from predicted vertical transitions. (b) Agreement of the experimental UV-vis absorption spectrum of **5d** (red curve) and the theoretical spectrum (blue curve).

Electrochemical data

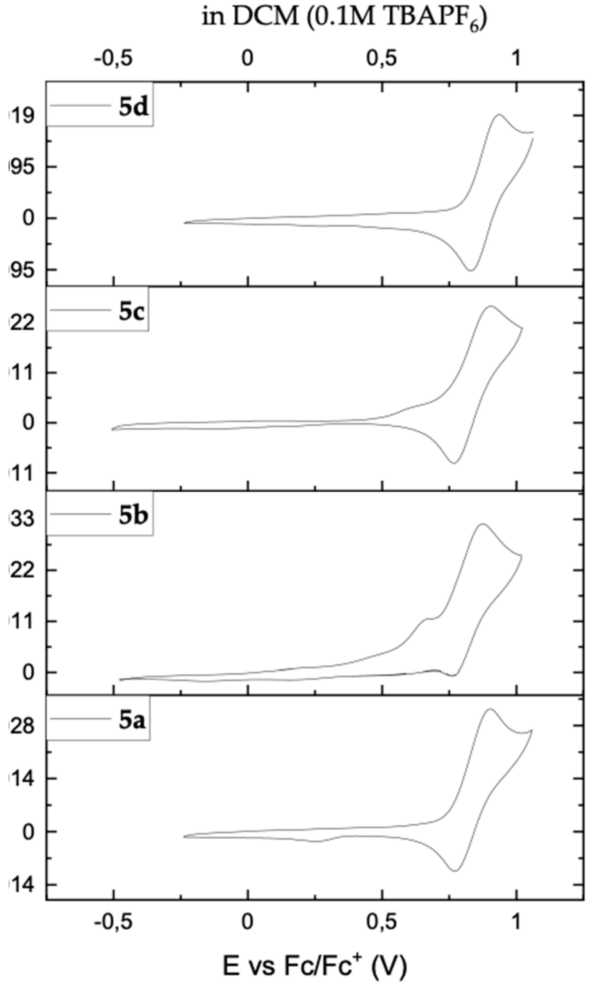

Figure S47. Cyclic Voltammetry of Cu(I) complexes **5a**, **5b**, **5c** and **5d** in dichloromethane (0,1 M $TBAPF_6$) at a scan rate of 100 mV/s. Y-axes: Current in mA.

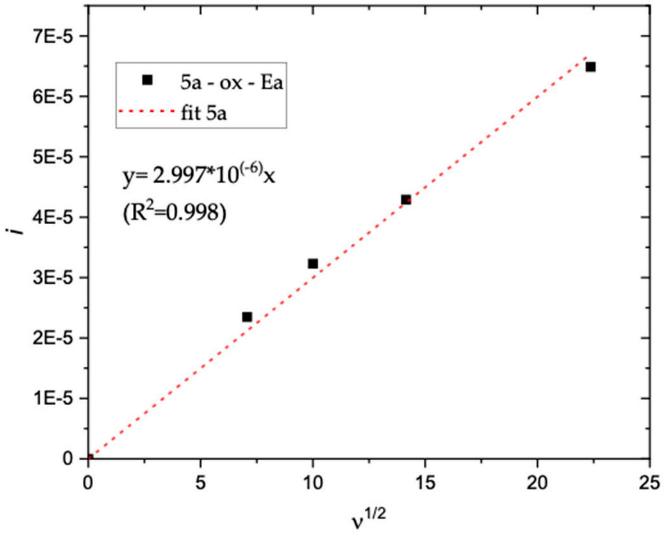

Figure S48. Randles-Sevcik Fit with the anodic peak current of **5a**, at a scan rate of 50, 100, 200 and 500 mV/s.

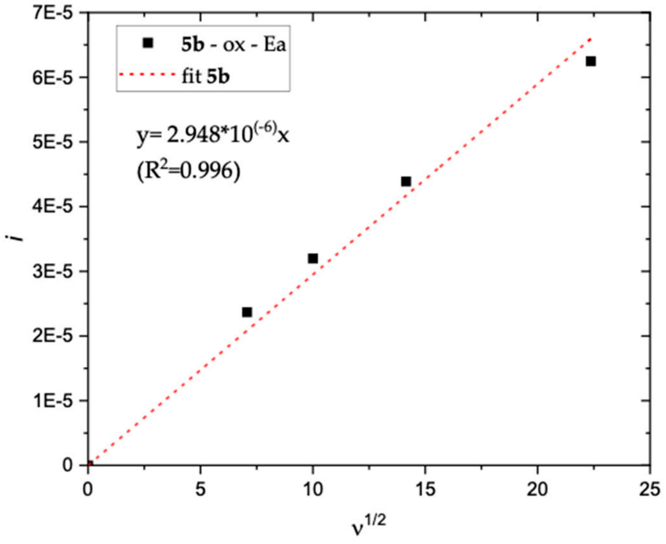

Figure S49. Randles-Sevcik Fit with the anodic peak current of **5b**, at a scan rate of 50, 100, 200 and 500 mV/s.

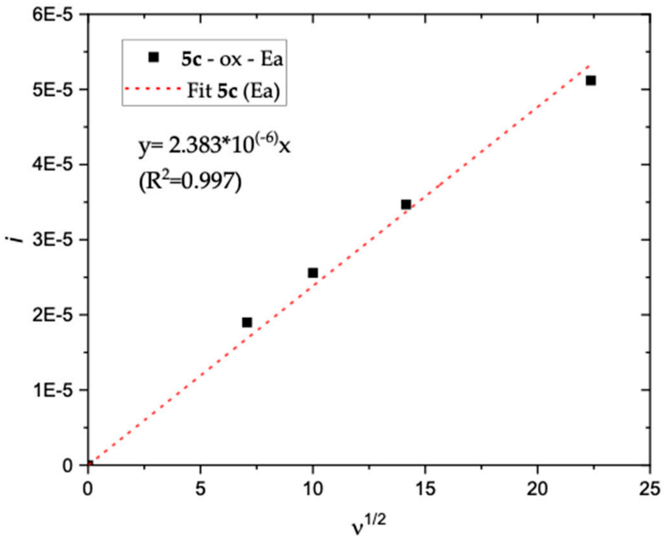

Figure S50. Randles-Sevcik Fit with the anodic peak current of **5c**, at a scan rate of 50, 100, 200 and 500 mV/s.

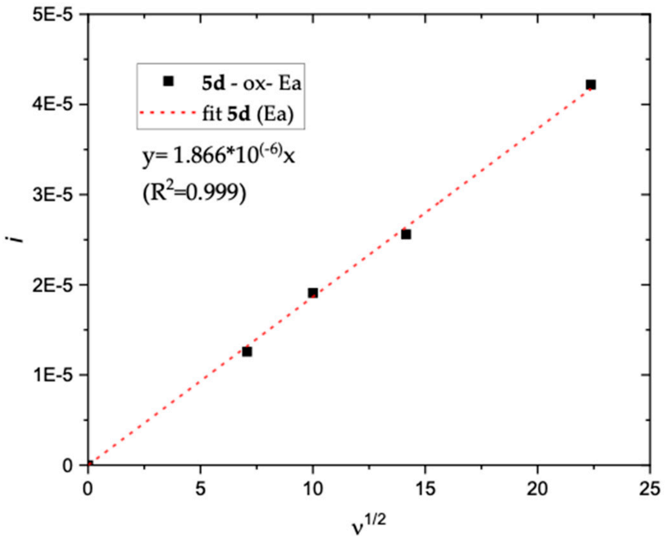

Figure S51. Randles-Sevcik Fit with the anodic peak current of **5d**, at a scan rate of 50, 100, 200 and 500 mV/s.

Photophysical analysis in solid state

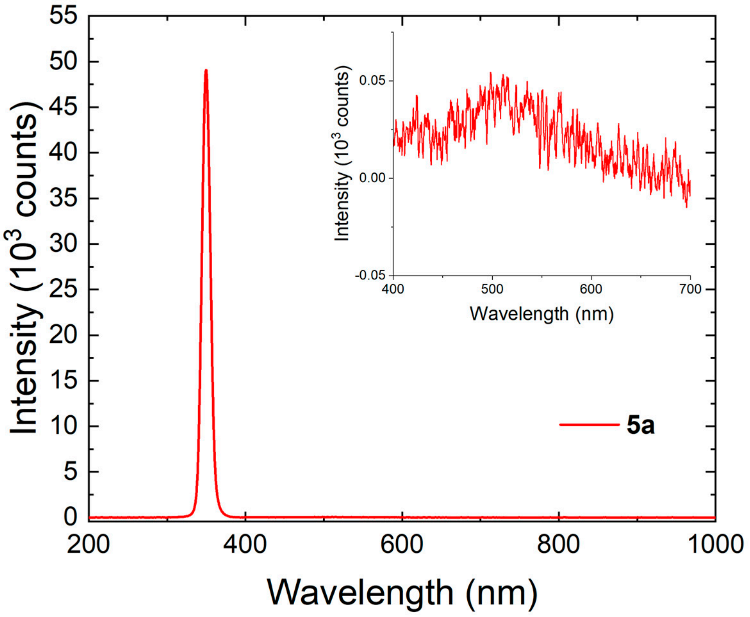

Figure S52. Emission spectrum of **5a** with incidence light of 350 ± 10 nm

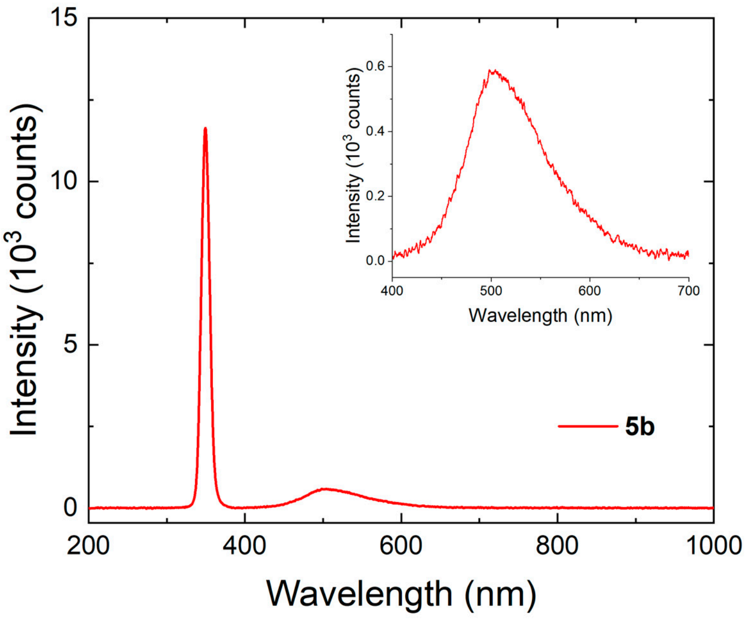

Figure S53. Emission spectrum of **5b** with incidence light of 350 ± 10 nm

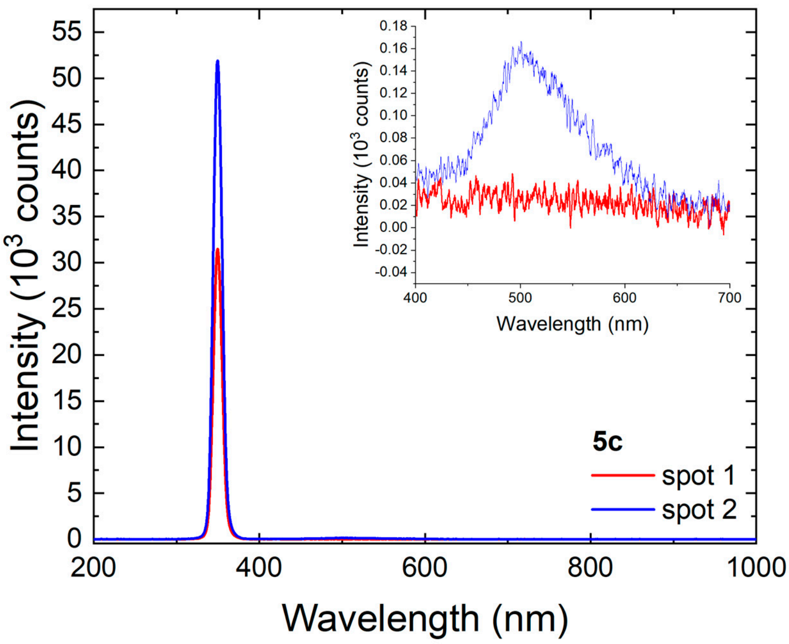

Figure S54. Emission spectrum of **5c** with incidence light of 350 ± 10 nm.

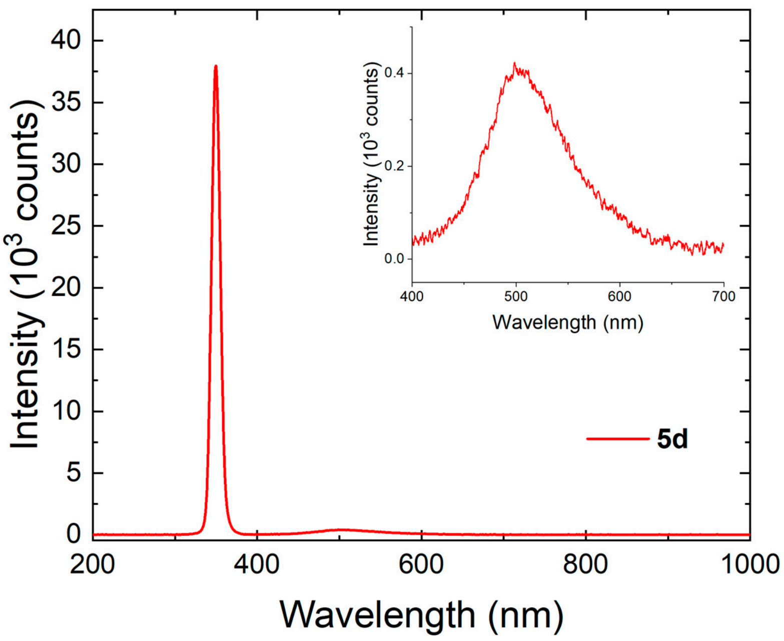

Figure S55. Emission spectrum of **5d** with incidence light of 350 ± 10 nm.

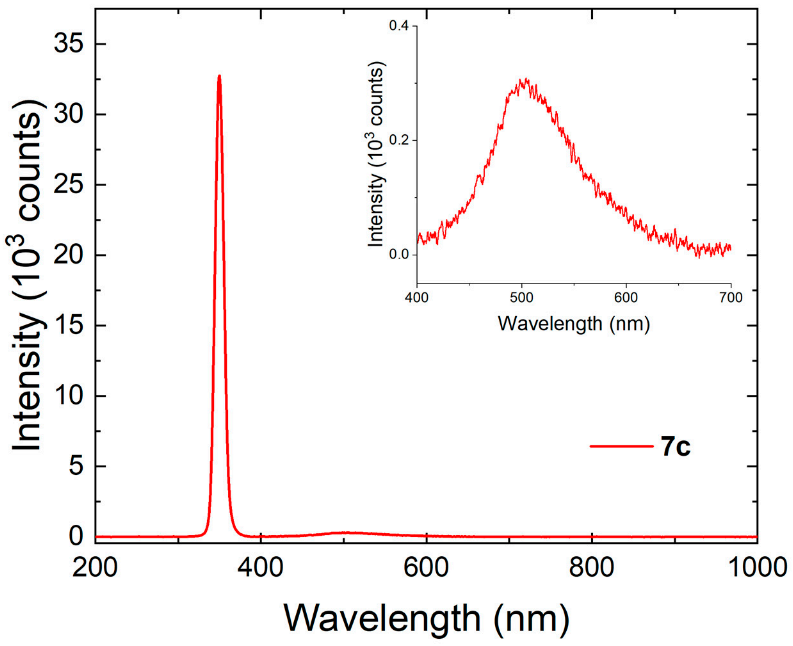

Figure S56. Emission spectrum of **7c** with incidence light of 350 ± 10 nm.

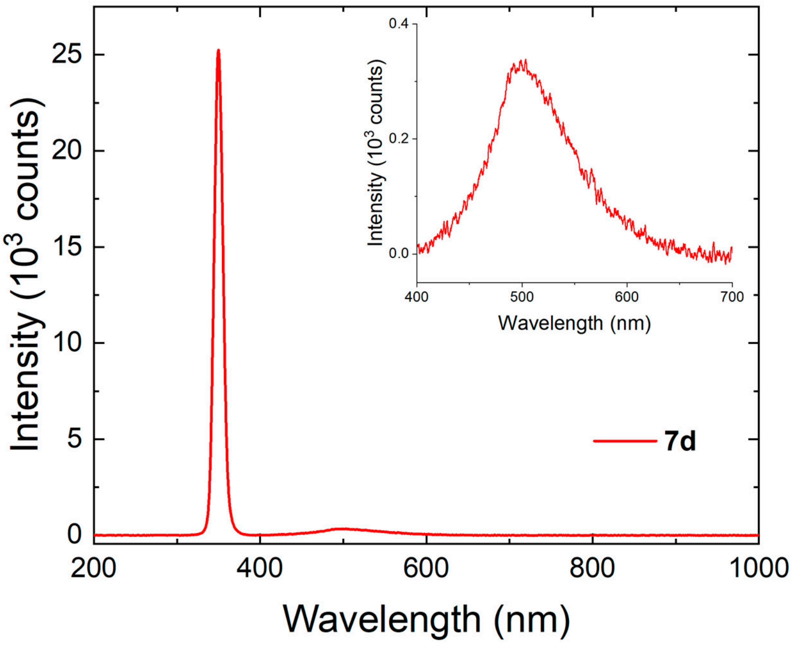

Figure S57. Emission spectrum of **7d** with incidence light of 350 ± 10 nm.

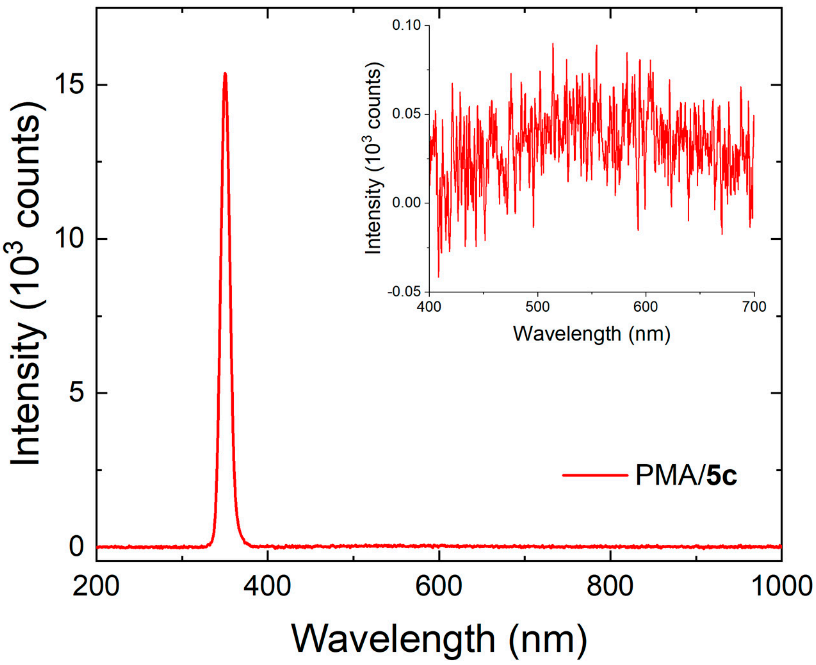

Figure S58. Emission spectrum of PMA/**5c** with incidence light of 350 ± 10 nm.

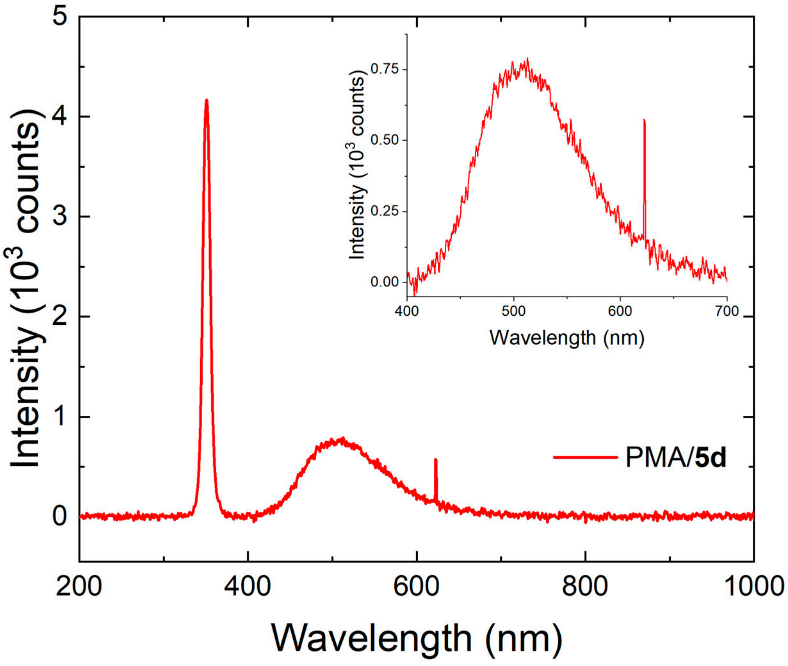

Figure S59. Emission spectrum of PMA/**5d** with incidence light of 350 ± 10 nm.

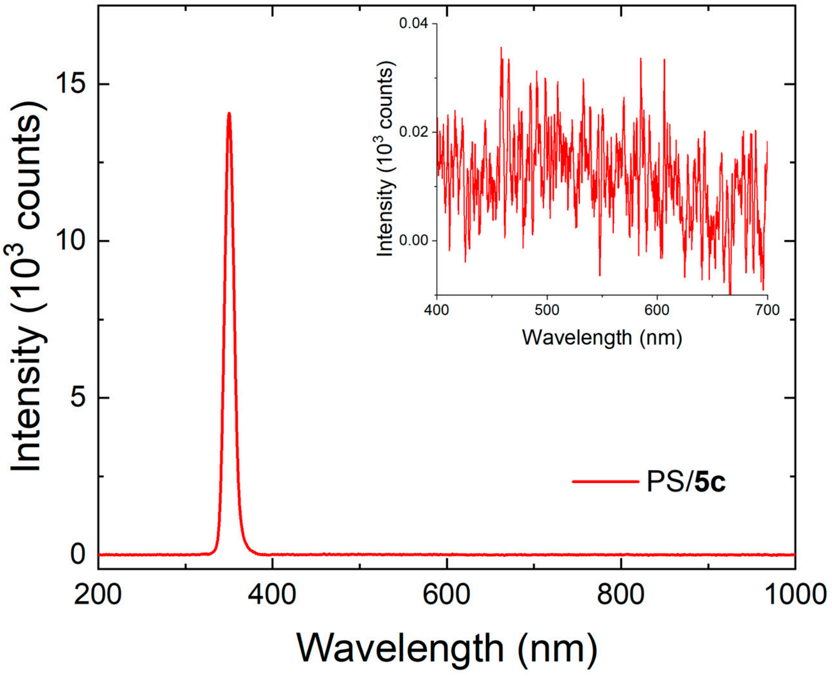

Figure S60. Emission spectrum of PS/**5c** with incidence light of 350 ± 10 nm.

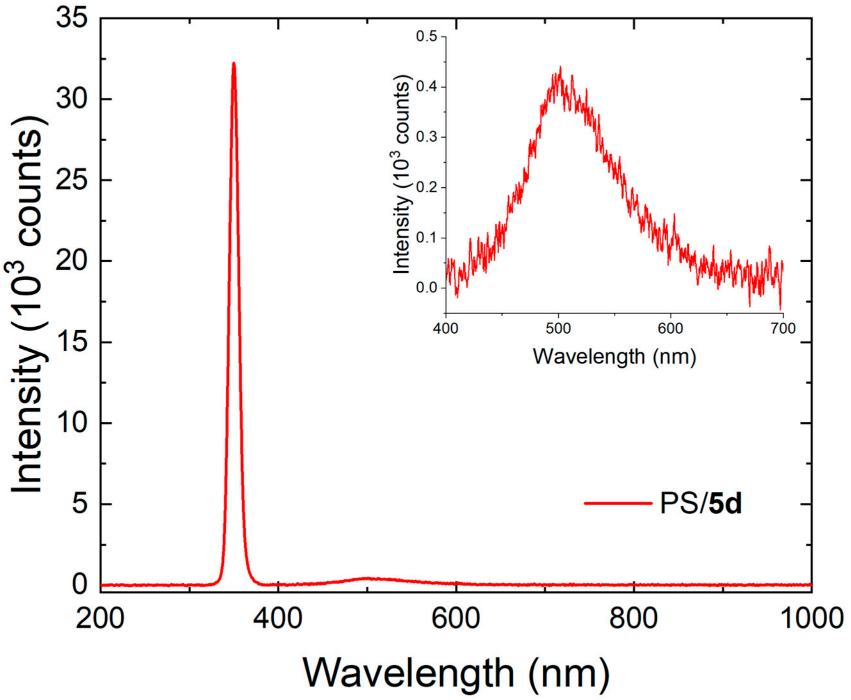

Figure S61. Emission spectrum of PS/**5d** with incidence light of 350 ± 10 nm.

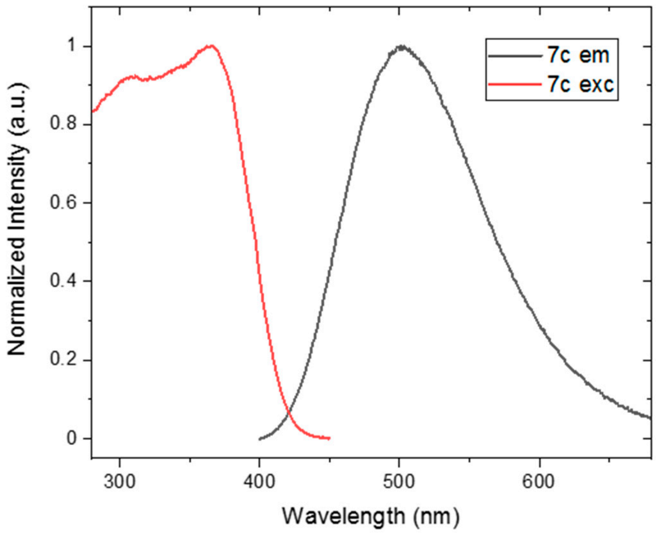

Figure S62. Emission (black) and excitation (red) spectra of **7c**. (Excitation at 350nm and emission at 500nm).

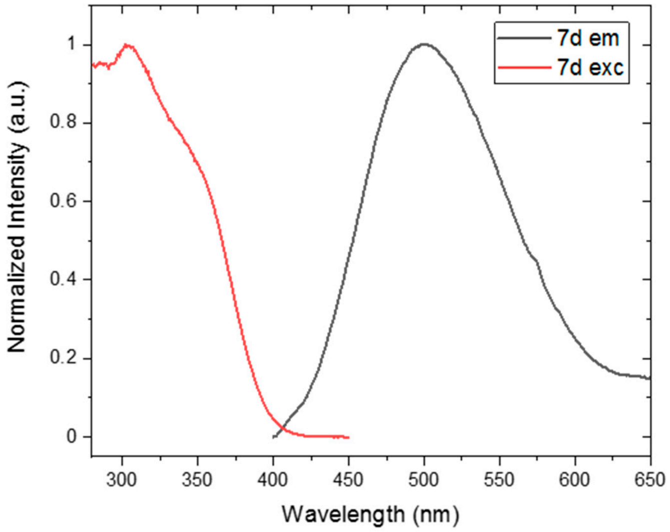

Figure S63. Emission (black) and excitation (red) spectra of **7d**. (Excitation at 350nm and emission at 500nm).

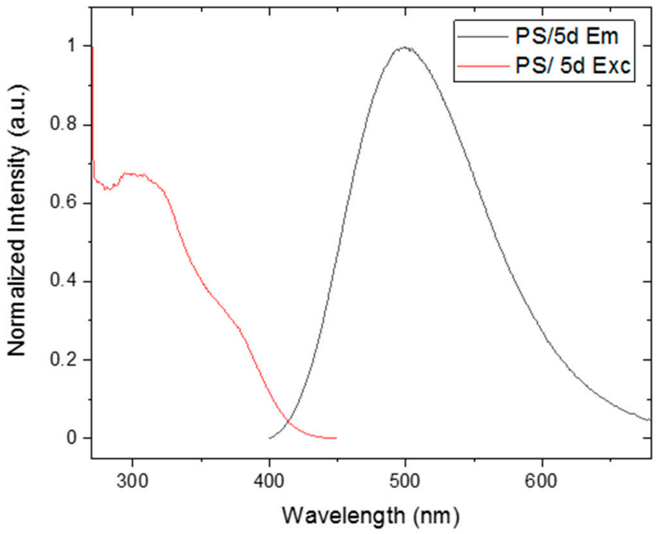

Figure S64. Emission (black) and excitation (red) spectra of PS/**5d**. (Excitation at 350nm and emission at 500nm).

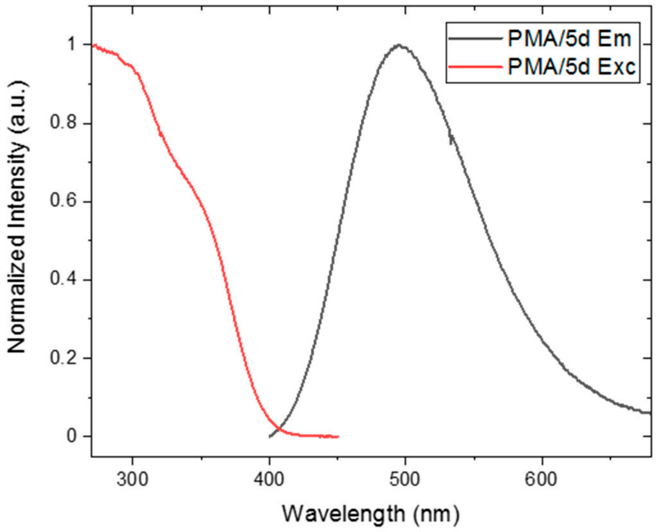

Figure S65. Emission (black) and excitation (red) spectra of PMA/**5d**. (Excitation at 350nm and emission at 500nm).

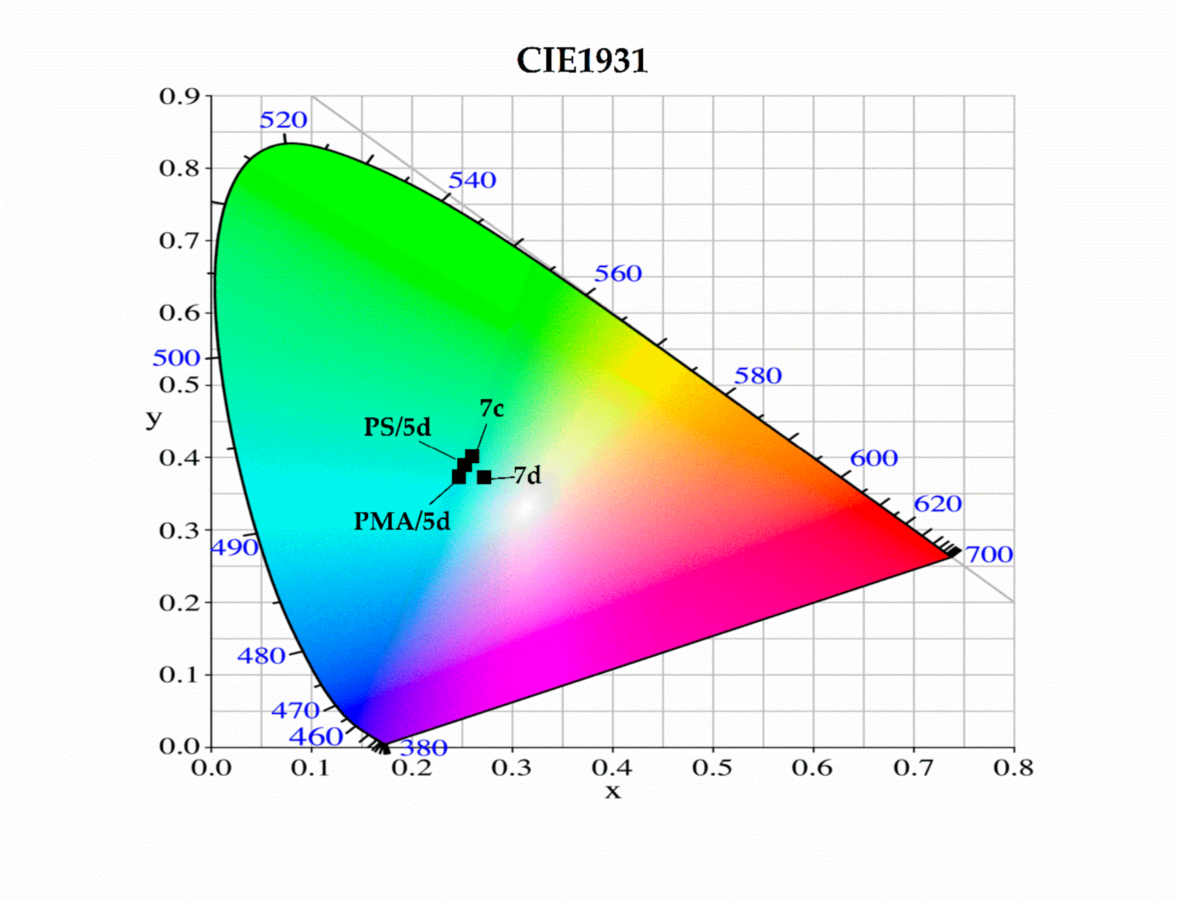

Figure S66. CIE 1931 chromaticity diagram for the copolymers **7c** and **7d** and the polymeric blends of PMA and PS with **5d**.

Atomic Force Microscopy measurements

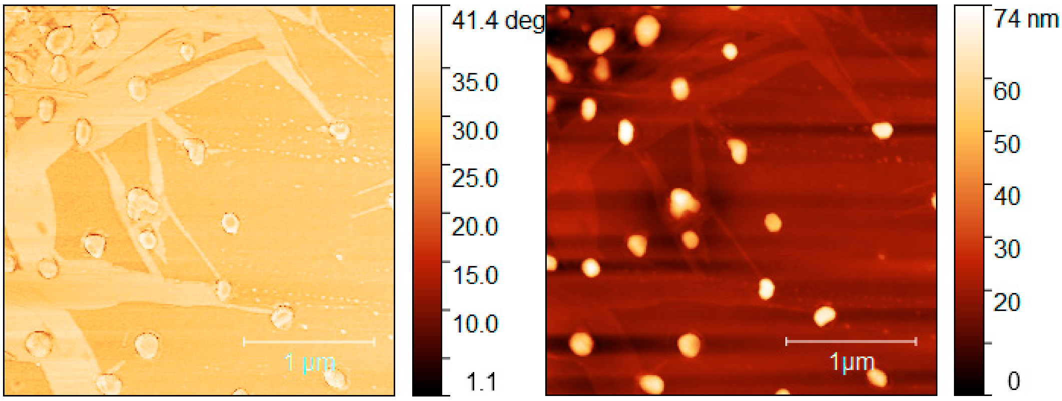

Figure S67. AFM phase (left) and height (right) images of PMA/**5c** which was drop-cast onto a glass slide. Some phase separation is observed and is attributed to the de-mixing of the polymer from the complex.

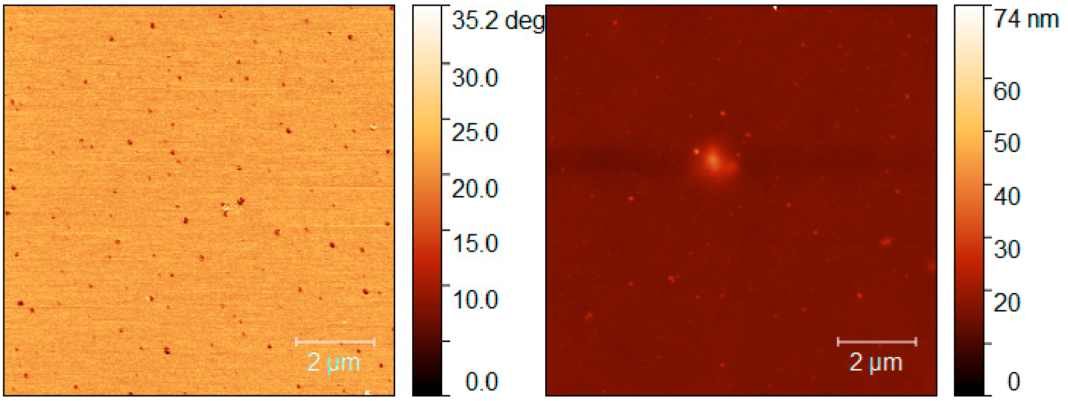

Figure S68. AFM phase (left) and height (right) images of PMA/**5d** which was drop-cast onto a glass slide. Some holes are present which are due to the non-optimized sample preparation method.

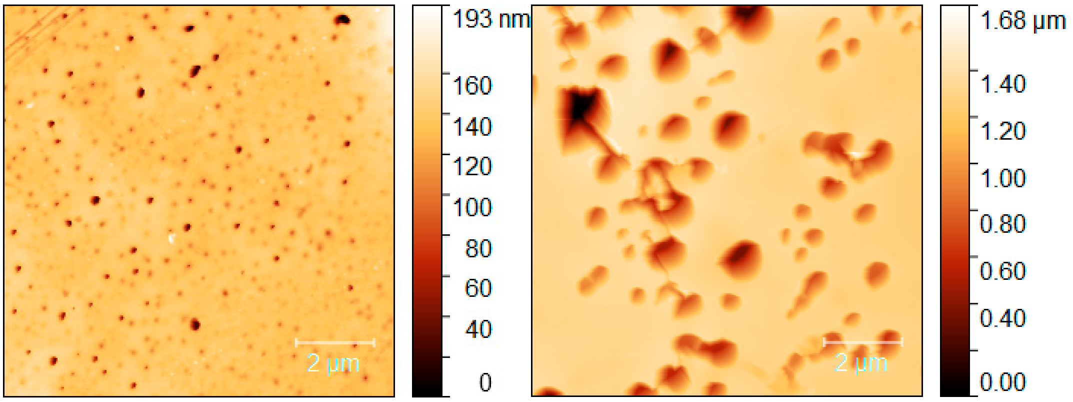

Figure S69. AFM phase (left) and height (right) images of **7c** which was drop-cast onto a glass slide. Some holes are present which are due to the non-optimized sample preparation method.

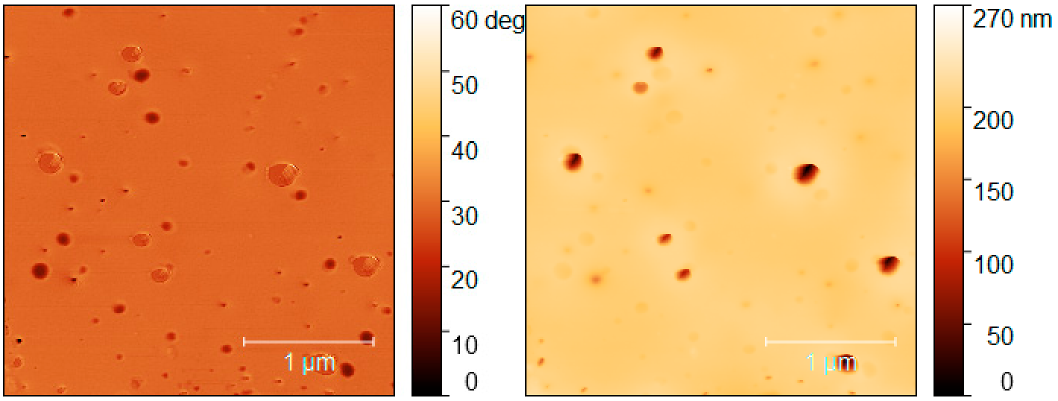

Figure S70. AFM phase (left) and height (right) images of **7d** which was drop-cast onto a glass slide. Some holes are present which are due to the non-optimized sample preparation method.

Optical microscopy

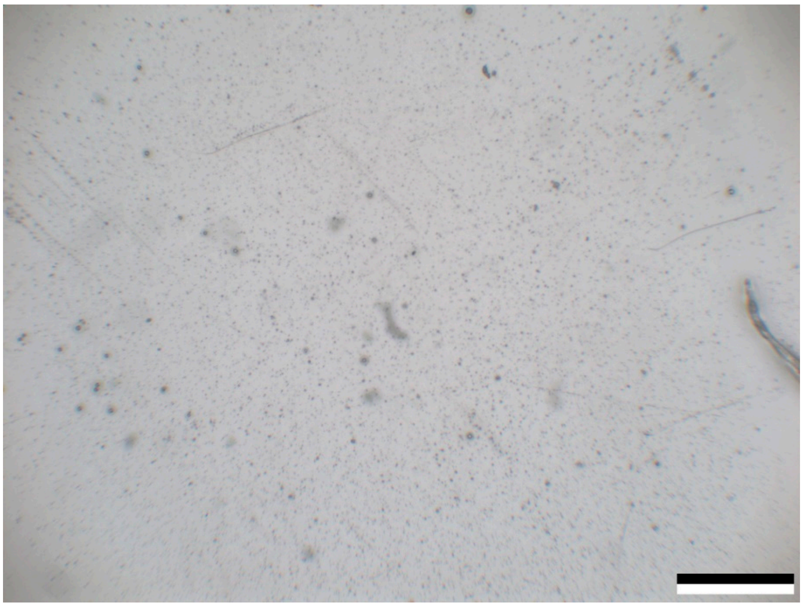

Figure S71. Optical microscopy image of PMA/**5c**. Scale bar 200 μm.

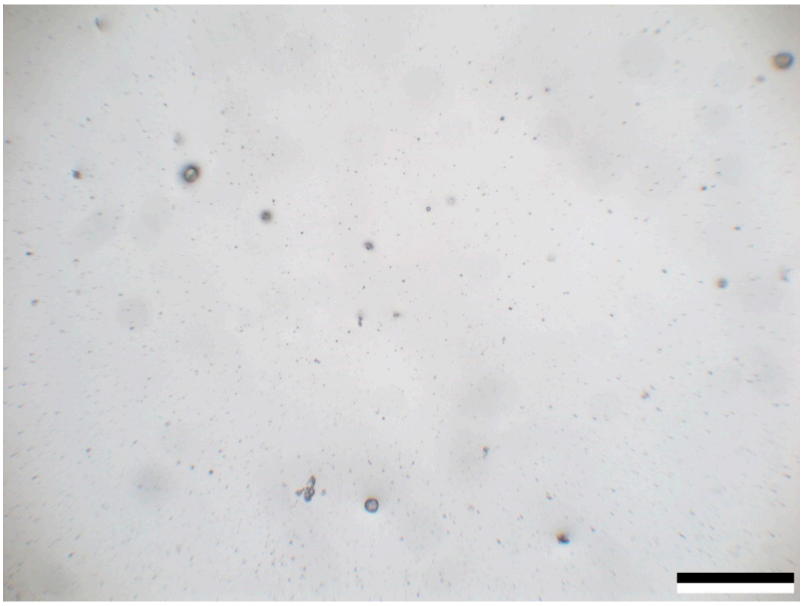

Figure S72. Optical microscopy image of PMA/**5d**. Scale bar 200 μm.

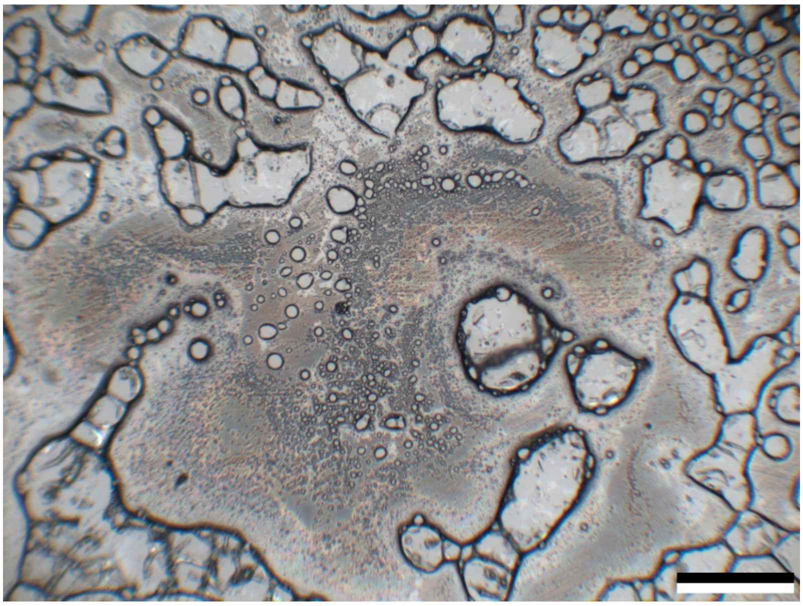

Figure S73. Optical microscopy image of **7c**. Scale bar 200 μm.

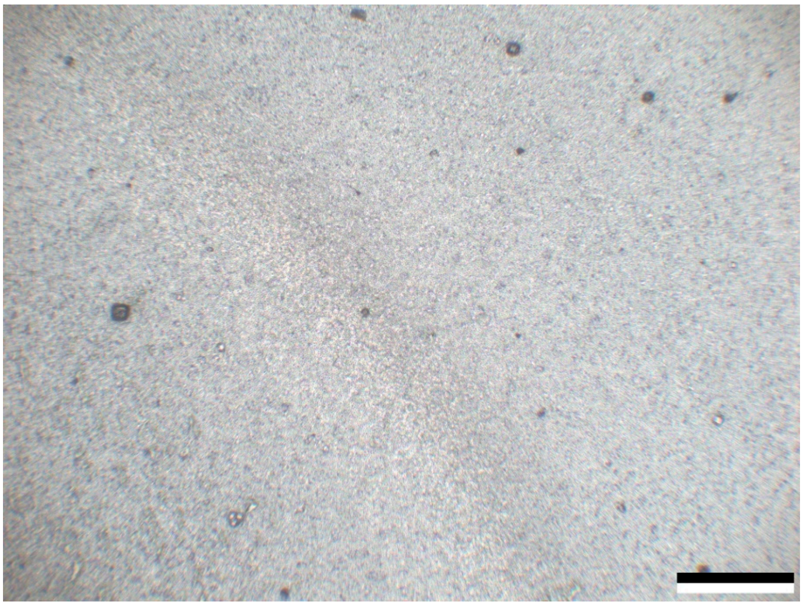

Figure S74. Optical microscopy image of **7d**. Scale bar 200 μm.

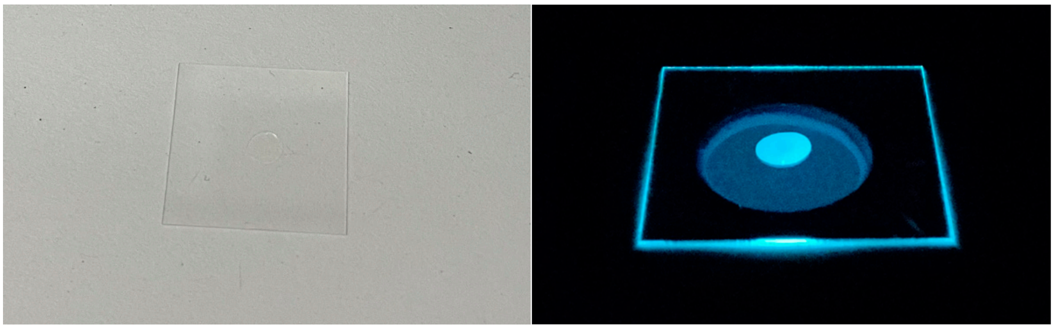

Figure S75. Photograph of drop-cast sample PMA/**5d** under ambient light (left) and UV irradiation at λ=350 nm (right).
